# Creative Arts Therapy for Anxiety, Depression, and Quality of Life in Cancer Patients: A Systematic Review and Meta‐Analysis of Randomized Controlled Trials

**DOI:** 10.1002/pon.70425

**Published:** 2026-03-19

**Authors:** Ashlyn S. L. Chou, Tyler McKechnie, Vikram Arora, Brianna DePestel, Austine Wang, Sameer Parpia, Goran Calic, Phillip Staibano, Alexandra Derus, Akanksha Guleria, Mohit Bhandari, Alex Thabane

**Affiliations:** ^1^ Michael G. DeGroote School of Medicine McMaster University Hamilton Ontario Canada; ^2^ Department of Surgery McMaster University Hamilton Ontario Canada; ^3^ Temerty Faculty of Medicine University of Toronto Toronto Ontario Canada; ^4^ Department of Health Research Methods, Evidence, and Impact McMaster University Hamilton Ontario Canada; ^5^ Department of Oncology McMaster University Hamilton Ontario Canada; ^6^ Michael G. DeGroote School of Business McMaster University Hamilton Ontario Canada

**Keywords:** anxiety, art, art therapy, cancer, depression, music therapy, oncology, psychosocial intervention, quality of life

## Abstract

**Background:**

Cancer is a significant psychological burden for patients. Previous evidence syntheses suggest creative arts therapies (CATs) may improve psychological outcomes, but are limited by heterogeneity in intervention types, study designs, and outcomes, and the lack of a certainty of evidence assessment.

**Aims:**

We conducted a robust systematic review and meta‐analysis of current randomized trial literature to explore the efficacy of CATs in improving anxiety, depression, and quality of life in cancer patients.

**Methods:**

We searched PubMed, Embase, and PsycInfo databases for peer‐reviewed randomized trials evaluating the effectiveness of CATs against a control in patients with current cancer diagnoses. We performed pairwise random‐effects meta‐analyses of standardized mean differences (SMD) for anxiety, depression, and quality of life, stratified by time‐interval. We conducted subgroup analyses by session frequency, intervention type, treatment setting, and region. For studies not pooled quantitatively, results were qualitatively summarized.

**Results:**

67 randomized trials with 6259 patients were included. The majority of interventions were music‐based (80.6%), multi‐session (59.7%), inpatient‐based (73.1%), and conducted in North America (29.9%). Meta‐analyses demonstrated positive effects of CATs on anxiety at < 7 days (SMD = −0.62 [95% CI −1.01, −0.24]), 4–6 weeks (−1.21 [−2.08, −0.34]), and 2–3 months (−1.19 [−2.14, −0.24]); depression at 1–3 weeks (−0.44 [−0.87, −0.00]) and 4–6 weeks (−1.14 [−1.76, −0.52]); and quality of life at 1–3 weeks (0.65 [0.05, 1.25]), 4–6 weeks (1.17 [0.02, 2.32]), 2–3 months (1.42 [0.55, 2.29]), and 4–6 months (0.42 [0.04, 0.80]. Qualitative results corroborate these findings. GRADE assessment revealed low‐to‐very‐low certainty of evidence.

**Conclusion:**

Creative arts therapies may improve anxiety, depression, and quality of life among cancer patients.

## 1 Introduction

The creative arts, which include dance‐movement, drama, film, music, and art [1], are typically pursued for the purposes of personal expression and enjoyment. But their psychological benefits are well established: engagement in the creative arts has been reported to improve well‐being, enhance life satisfaction, and reduce stress [2, 3]. Given such benefits, there has naturally been great interest in the use of the creative arts as a therapeutic intervention.

The use of the creative arts for therapeutic purposes has a long and rich history. The ancient Chinese are known to have used music to treat the sick – in fact, the Chinese character for medicine, *yào*, is derived from the character for music, *yuè* [4]. Similarly, the Egyptians performed the traditional *Zar* dance for the purposes of healing [5]. This tradition of using the creative arts for healing persists today through what we now term ‘creative arts therapies’ (CATs). The American Psychological Association (APA) defines CATs as “therapeutic interventions that use artistic endeavors or mediums, such as music, poetry, dance, and drama, to facilitate communication and emotional expression, enhance self‐awareness, and foster health and change.” [6] CATs have been utilized in a range of patient populations, and to great effect: meta‐analyses have found CATs to significantly improve psychological outcomes for patients with depression [7, 8], post‐traumatic stress disorder [9], and cognitive decline [10, 11]. Given the evidence of efficacy in improving outcomes in these populations, CATs have also been trialled in patients with another psychologically taxing condition: cancer.

In more ways than one, cancer is a considerable trial for patients. The strain of such a life‐threatening illness often leads to many experiencing symptoms of psychological distress: anxiety and depression prevalence rates over 20% have been reported [12, 13]. These psychological disorders are particularly worrisome as they can reduce adherence to treatment [14] and even increase the risk of mortality [15]. Thus, improving the psychological state of cancer patients could have a meaningful impact on prognosis. CATs provide a promising intervention to do so.

Over the last 15 years, substantial research has been conducted on the use of CATs in cancer patients, facilitating in the performance and publication of several systematic reviews and meta‐analyses [16, 17, 18, 19, 20, 21, 22]. While these evidence syntheses in general suggest a benefit of CATs, the findings across these studies are often conflicting. More, there is substantial heterogeneity across reviews in the types of study designs included and outcomes assessed, and most do not explore any clinically relevant subgroup effects (i.e., frequency of sessions, type of CAT, setting, region), which limits the ability to conclude which types of CATs are most effective for which patients, in which outcomes and settings. Thus, there is a clear need for a new meta‐analytic synthesis of the literature, particularly one focused on randomized controlled trials and exploring the differential effectiveness of CATs by session frequency, type of CAT, intervention setting, and region. We conducted the present study to fill this gap. We hypothesize, based on the existing literature [17, 18], that CATs will be effective in reducing anxiety and depression and improving quality of life.

## 2 Methods

This systematic review and meta‐analysis is reported in accordance with the Preferred Reporting Items for Systematic Reviews and Meta‐Analyses (PRISMA) [23]. We registered this review on PROSPERO (CRD420251087592). As an analysis of secondary aggregate data, we did not require ethics approval for this study.

### 2.1 Objectives

The primary objective of this systematic review and meta‐analysis was to evaluate the efficacy of CATs in improving anxiety, depression, and quality of life in cancer patients. As secondary objectives, we sought to explore whether there were subgroup effects from the following characteristics: session frequency (single vs. multi‐session), intervention type (art vs. music vs. other types of CAT), treatment setting (inpatient vs. outpatient), and region (continent).

### 2.2 Eligibility Criteria

We considered studies that met the following criteria eligible for this review: (1) Randomized controlled trials; (2) exploring a CAT intervention of any kind (i.e., music, visual art, dance/movement, writing); (3) conducted in adult subjects (i.e., 18 years of age or older) with a current diagnosis of cancer; (4) measured one or more of anxiety, depression, and quality of life; and (5) published in a peer‐reviewed journal. Studies were excluded if they were published as abstracts or conference proceedings, were secondary analyses, included patients in remission from cancer, or were not published in English.

### 2.3 Search Strategy

We searched the MEDLINE, Embase, and PsycInfo databases from inception through February 2025 for eligible articles. We combined Medical Subject Heading (MeSH) terms (e.g., neoplasms, Oncology) and key words (e.g., creative arts therapy, movement therapy, art therapy, music therapy), using the Boolean operators AND/OR. Our search strategy can be found in Supporting Information S1: Appendix A1.

### 2.4 Study Selection Process

Four reviewers (VA, BD, AC, AW) performed title and abstract screening, and subsequently full‐text review, independently and in duplicate using the Covidence online platform. Any discrepancies were resolved by discussion until consensus, and if necessary, adjudication by the senior author (AT).

#### 2.4.1 Data Collection Process

Similar to the study selection process, a team of three reviewers (AC, VA, BD) performed data extraction independently and in duplicate using a custom data collection form developed using Microsoft Excel. Any discrepancies were resolved by the senior author (AT). Extracted data included study characteristics (i.e., author, year of publication, study design, location of study, number of included centers, study inclusion/exclusion criteria, primary outcomes), patient demographics (e.g., age, sex), disease characteristics (e.g., type of cancer, stage of malignancy), intervention characteristics (e.g., type, number of sessions, duration of treatment), and outcome data at all available time‐points for the outcomes of interest.

#### 2.4.2 Outcomes

The primary outcomes of interest in this review were anxiety, depression, and quality of life. We defined anxiety as an “uncontrollable, diffuse, unpleasant, and persistent state of negative affect, characterized by apprehensive anticipation regarding unpredictable and unavoidable future danger, and accompanied by physiological symptoms of tension and a constant state of heightened vigilance” [24, 25]. We defined depression as a mood disorder characterized by sadness, emptiness, or irritable mood, accompanied by somatic and cognitive changes that significantly affect the individual's capacity to function [26]. We defined quality of life using the World Health Organizations definition of “individual's perception of their position in life in the context of the culture and value systems in which they live and in relation to their goals, expectations, standards and concerns” [27].

#### 2.4.3 Risk of Bias & Certainty of Evidence

To assess the risk of bias in the included studies, we used the Cochrane Risk of Bias Tool for Randomized Controlled Trials 2.0, which consists of five domains: (1) bias arising from the randomization process, (2) bias due to deviations from intended interventions, (3) bias due to missing outcome data, (4) bias in measurement of the outcome, and (5) bias in selection of the reported result [28]. Two reviewers (AC, VA) assessed the risk of bias of each study independently and in duplicate, with discrepancies resolved by a third reviewer (AT). For each outcome, the senior author (AT) assessed the certainty of the evidence using the Grading of Recommendations Assessment, Development and Evaluation (GRADE) framework [29]. We reported the results of the risk of bias and GRADE assessments in tables.

### 2.5 Statistical Analysis

To meet our primary objective, we conducted pairwise, DerSimonian and Laird random‐effects meta‐analyses of standardized mean differences (SMD) for each of the outcomes of interest. To perform these analyses, we utilized mean scores with the associated standard deviation (SD). If standard errors were reported, we converted them to standard deviations by multiplying the value by the square root of the sample size.

As each study reported data at variable time‐points, we stratified the analyses for each outcome into time‐point intervals based on the most frequently reported follow‐up timings across the included studies. This resulted in the following time‐points: immediate (0–6 days post‐intervention), 1–3 weeks post‐intervention, 4–6 weeks post‐intervention, 2–3 months post‐intervention, and 4–6 months post‐intervention. In the case of randomized trials with more than two experimental groups (i.e., CATs), we included the pairwise comparison of both experimental groups with the control group as separate studies (e.g., Arruda 2016a and Arruda 2016b). We assessed the statistical heterogeneity within each meta‐analyses via the *I* ^2^ statistic, considering an *I* ^2^ greater than 40% to represent substantial heterogeneity [30]. Finally, where more than 10 studies were included in a meta‐analysis, we assessed the risk of publication bias via visual inspection of a funnel plot. The pooled SMD from each analysis, with the associated 95% confidence interval (95% CI) are reported in forest plots and tables.

To meet our secondary objective, we conducted four exploratory subgroup meta‐analyses for each outcome (and time‐point) based on the following baseline characteristics: intervention type (i.e., art therapy; music therapy; other), session frequency (i.e., single session; multi‐session), setting (i.e., inpatient, outpatient), and region (i.e., North America; South America; East Asia; West Asia; Europe; Africa). The results of the subgroup analyses are presented narratively and in forest plots. Due to the large number of planned subgroup comparisons across multiple outcomes, subgroups, and time‐points, we felt that formal hypothesis testing using *p*‐values would substantially increase the risk of false findings and reduce interpretability. We therefore focused on estimation rather than significance testing, with a subgroup considered to show evidence of benefit when the confidence interval of the effect size estimate did not cross zero.

We performed all analyses independently and in duplicate to minimize the risk of human error. The meta‐analyses were conducted in STATA Version 15 (TM) and R Software (version 4.3.2) via RStudio (AT) using the *metagen* and *forest* packages.

### 2.6 Qualitative Analysis

For studies that were unable to be included in the meta‐analyses due to lack of reporting of the necessary statistics, we qualitatively analyzed their results to determine whether a significant improvement was observed in each of the three outcomes of interest. We categorized each result in one of three categories: a significant positive benefit with CATs (green label), no significant effect with CATs (gray label), or a significant negative effect with CATs (red label). The results are summarized narratively and reported in a table.

## 3 Results

### 3.1 Literature Search

**FIGURE 1:**
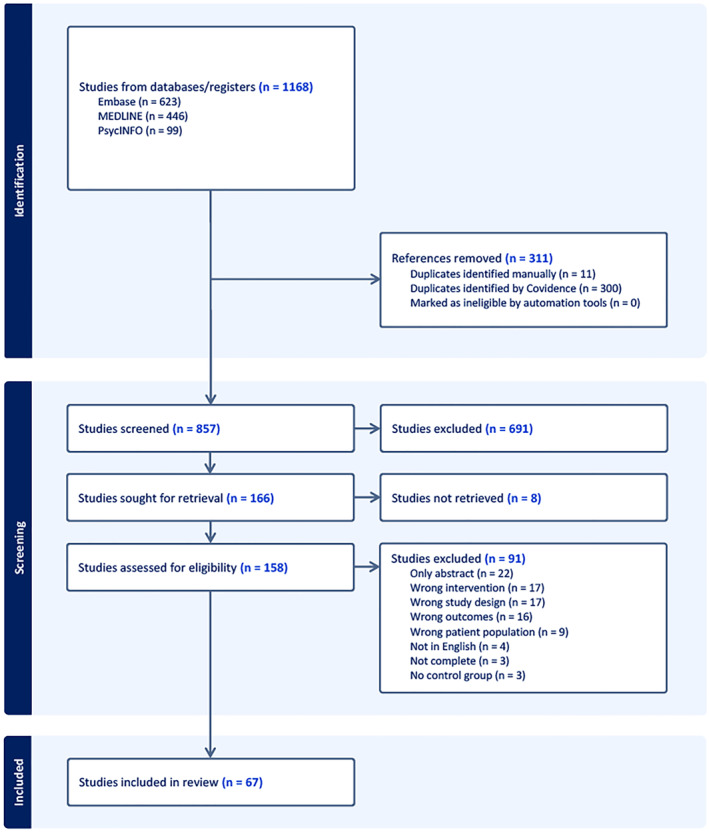
Prisma flow diagram.

We identified 1168 studies in our database search. After removal of duplicates and title and abstract screening, 166 studies progressed to full‐text review. Upon completion of full text review, we included a total of 67 studies in the systematic review. The full literature search details can be found in Figure 1.

#### 3.1.1 Study Characteristics

The 67 randomized trials, which were published between 2003 and 2024, included a total of 6259 cancer patients. Most were simple, two‐arm parallel randomized trials (*n* = 56), with some three‐arm designs (*n* = 10), and one four‐arm design. A modest proportion were conducted in the United States (*n* = 20), followed by Turkey (*n* = 10) and China (*n* = 10). Studies most commonly recruited breast cancer patients (*n* = 25) or included all types of cancer patients (*n* = 18). The most common CAT type studied was music therapy (i.e., music listening or music‐making interventions; *n* = 54). The majority of interventions were multi‐session (*n* = 40) and took place in an inpatient setting (*n* = 49). In almost every study, the control group was simply the standard of care (*n* = 62); however, one study utilized a sham intervention [31], one intravenous midazolam [32], one oral escitalopram [33], and two meditation [34, 35]. Duration of follow‐up ranged from immediately post‐intervention to 12 months post‐intervention. A complete table of study details can be found in Table 1.

**TABLE 1:** Study characteristics.

First author Year Country	Setting	Design	Outcomes	Cancer	Stage	Arm	N	Mean age (SD)	% female	Duration, # of sessions
Alcântara‐silva 2018 Brazil [36]	Cancer center	Parallel group	Depression (BDI), QoL (FACT‐G)	Breast or gynecological	NR	Music therapy	53	51.85 (10.6)	100	5+ weeks, 10+ sessions
						Control	63	52.9 (10.26)	100	
Akbulak 2023 Turkey [37]	Outpatient chemotherapy unit	Parallel group	Anxiety (STAI)	Breast	I‐III	Mandala coloring	41	46.1 (7.7)	100	1 day, 1 session
						Control	43	50.8 (9.2)	100	
Alam 2016 United States [38]	Hospital	Parallel group	Anxiety (STAI)	Basal or cutaneous squamous cell carcinoma of the face	NR	Guided imagery	50	62.7 (NR)	46.00	1 day, 1 session
						Pre‐recorded music listening	54	62.4 (NR)	40.74	
						Control	51	64.2 (NR)	39.22	
An 2022 China [33]	Hospital	Parallel group	Depression (HAMD‐24), QoL (QLQ‐C30)	Any	NR	Chaihu plus longgu muli decoction with five‐element music therapy	60	56.05 (8.73)	38.33	20 days, 40 sessions
						Control (escitalopram 10 mg/d)	60	55.76 (8.81)	41.67	
Arruda 2016 Brazil [39]	Hospital	Parallel group	Depression (BDI)	Any	NR	Pre‐recorded music listening	22	NR (NR)	72.73	3 days, 3 sessions
						Pre‐recorded recited poem listening	22	NR (NR)	72.73	
						Control	21	NR (NR)	71.43	
Bates 2017 United States [32]	Hospital	Parallel group	Anxiety (POMS‐tension), depression (POMS‐depression)	Multiple myeloma or lymphoma	NR	Music therapy	37	58^a^ (NR)	51.35	6 days, 2 sessions
						Control	45	58^a^ (NR)	40.00	
Binns‐turner 2011 United States [40]	Hospital	Parallel group	Anxiety (SAI)	Breast	NR	Pre‐recorded music listening	15	56.63^b^ (NR)	100	1 day, 1 session
						Control	15	56.63^b^ (NR)	100	
Bradt 2024 United States [41]	Hospital	Parallel group	Anxiety (PROMIS anxiety)	Any	III‐IV	Music therapy	45	57.4 (11.54)	73.33	12 weeks, 6 sessions
						Control (social attention)	47	54.94 (11.7)	70.21	
Bro 2019 Denmark [42]	Hospital	Parallel group	Anxiety (STAI‐Y), QoL (EORTC‐QLQ‐30)	Hodgkin or non‐Hodgkin lymphoma	NR	Live music listening	47	59 (18)	51.06	NR, 5 sessions
						Pre‐recorded music listening	47	61 (15)	40.43	
						Control	49	59 (18)	40.82	
Bulfone 2009 Italy [43]	Hospital	Parallel group	Anxiety (STAI‐Y)	Breast	I‐II	Pre‐recorded music listening	30	49.2 (6.9)	100	1 day, 1 session
						Control	30	52.7 (6.1)	100	
Cassileth 2003 United States [44]	Cancer center	Parallel group	Anxiety (POMS‐tension), depression (POMS‐depression)	Hematologic	NR	Music therapy	36	53 (11)	38.89	10 days, 5 sessions
						Control	33	51 (12)	69.70	
Chen 2013 Taiwan [45]	Hospital	Parallel group	Anxiety (STAI)	Head and neck, gynecological, breast, digestive tract, lung, or prostate	I‐IV	Pre‐recorded music listening	100	55.06 (13.5)	36.00	1 day, 1 session
						Control	100	55.66 (11.41)	43.00	
Chen 2018 Taiwan [46]	Meditation room (group music therapy) or home (self‐directed music therapy)	Parallel group	Anxiety (HADS‐A), depression (HADS‐D)	Breast	NR	Group music therapy	20	NR (NR)	100	8 weeks, 8 sessions
						Self‐directed music therapy	20	NR (NR)	100	
						Control	20	NR (NR)	100	
Chen 2020 Taiwan [47]	Outpatient chemotherapy unit	Parallel group	Anxiety (HADS‐A), depression (HADS‐D)	Breast	0‐II	Music therapy	50	51.16 (9.21)	100	1 day, 1 session
						Control	50	49.56 (9.78)	100	
Cho 2024 South Korea [48]	Hospital	Parallel group	Anxiety (STAI)	Bladder	NR	Pre‐recorded music listening	80	69.3 (9.7)	0	1 day, 1 session
						Control	80	69.2 (12.6)	0	
Chuang 2024 Taiwan [49]	Hospital	Parallel group	Anxiety (STAI), QoL (WHOQOL‐BREF)	Breast	0‐IV	Pre‐recorded music listening	63	52 (10)	100	12 weeks, 12 sessions
						Control	65	53.2 (9.6)	100	
Clark 2006 United States [50]	Cancer center	Parallel group	Anxiety (HADS‐A), depression (HADS‐D)	Any	NR	Pre‐recorded music listening	35	58.61 (13.8)	37.14	1 day, 1 session
						Control	28	56.71 (11.0)	39.29	
Conrad 2016 United States [51]	Hospital	Parallel group	Anxiety (VAS)	Breast	NR	Live music listening	68	58.8 (14.4)	100	1 day, 1 session
						Pre‐recorded music listening	68	58.2 (17.6)	100	
						Control	65	61.3 (14.9)	100	
Czamanski‐Cohen 2019 Israel [31]	Cancer center	Parallel group	Depression (CES‐D)	Breast	NR	Art therapy	4	NR (NR)	100	8 weeks, 8 sessions
						Mandala coloring (sham art therapy)	5	NR (NR)	100	
Danhauer 2010 United States [52]	Cancer center	Parallel group	Anxiety (STAI)	Hematologic	NR	Pre‐recorded music listening	29	50.2 (14.1)	65.52	1 day, 1 session
						Control	30	51.6 (13.8)	53.33	
Deng 2022 China [53]	Hospital	Parallel group	Anxiety (VAS)	Breast	NR	Aromatherapy	40	54.4 (11.5)	100	1 day, 1 session
						Pre‐recorded music listening	40	54.2 (9.8)	100	
						Aromatherapy + pre‐recorded music listening	40	52.4 (12.5)	100	
						Control	40	50.2 (10.6)	100	
Du 2024 China [54]	Hospital	Parallel group	Anxiety (STAI)	Non‐small cell lung carcinoma	I‐IV	Music therapy	31	57 (NR)	22.58	1 day, 2 sessions
						Control	31	57 (NR)	25.81	
Duzgun 2024 Turkey [55]	Hospital	Parallel group	Anxiety (STAI)	Lung, pancreas, gastric, colon, or breast	NR	Music therapy	30	NR (NR)	20.00	NR, 6 sessions
						Control	30	NR (NR)	20.00	
Ferrer 2007 United States [56]	Outpatient chemotherapy unit	Parallel group	Anxiety (VAS)	Any	NR	Live music listening	25	NR (NR)	NR	1 day, 1 session
						Control	25	NR (NR)	NR	
Firmeza 2017 Brazil [57]	Outpatient clinic	Parallel group	Anxiety (STAI)	Head and neck	NR	Pre‐recorded music listening	20	NR (NR)	NR	1 day, 1 session
						Control	20	NR (NR)	NR	
Gezgin Yazici 2024 Turkey [58]	Outpatient chemotherapy unit	Parallel group	Anxiety (STAI)	Any	NR	Pre‐recorded music listening	30	60.13 (10.77)	50.00	4–6 weeks^c^, 3 sessions
						Control	30	61.07 (13.78)	40.00	
Giordano 2023 Italy [32]	Hospital	Parallel group	Anxiety (VAS)	Micro‐invasive oral cancer	I‐II	Music therapy	30	61 (12)	93.33	1 day, 1 session
						Control (IV midazolam 0.02 mg/kg)	30	61 (12)	86.67	
Hanser 2006 United States [59]	Cancer center	Parallel group	Anxiety (HADS‐A), depression (HADS‐D), QoL (FACT‐G)	Breast	IV	Music therapy	35	53^a^ (NR)	100	9–15 weeks, 3 sessions
						Control	35	50^a^ (NR)	100	
He 2022 China [60]	Hospital	Parallel group	QoL (FACT‐B)	Breast	I‐III	Dance therapy	88	47.99 (8.62)	100	16 weeks, 86 sessions
						Control (health consultation)	88	48.32 (10)	100	
Hilliard 2003 United States [61]	In‐home hospice	Parallel group	QoL (HQLI‐R)	Any	NR (terminal)	Music therapy	40	66 (NR)	50.00	NR, 2‐13 sessions
						Control (routine hospice care)	40	65 (NR)	50.00	
Ho 2016 Hong Kong [62]	Hospital and community cancer support center	Parallel group	Anxiety (HADS‐A), depression (HADS‐D), QoL (FACT‐B)	Breast	0‐III	Dance therapy	69	48.6 (7.7)	100	3 weeks, 6 sessions
						Control	70	49.1 (8.7)	100	
Jang 2016 South Korea [63]	Clinic	Parallel group	Anxiety (PAI‐A), depression (PADI‐D), QoL (EORTC‐QLQ‐30)	Breast	0‐III	Mindfulness‐based art therapy	12	51.75 (5.32)	100	12 weeks, 12 sessions
						Control	12	51.42 (6.33)	100	
Joly 2022 France [64]	Hospital	Parallel group	QoL (FACT‐G)	Breast	NR	Art therapy	125	55 (8)	100	8 weeks, 8 sessions
						Control	127	55 (9)	100	
Karadag 2019 Turkey [65]	Outpatient radiation oncology clinic	Parallel group	Anxiety (HADS‐A), depression (HADS‐D)	Breast	I‐II	Pre‐recorded music listening	30	62.2 (12.49)	100	5 weeks, 25 sessions
						Control	30	56.6 (13.66)	100	
Kwekkeboom 2003 United States [66]	Outpatient oncology clinic	Parallel group	Anxiety (STAI)	Any	NR	Distraction (listening to book on tape)	14	55.5 (14.12)	85.71	1 day, 1 session
						Pre‐recorded music listening	24	51.96 (15.21)	62.50	
						Control	20	53.3 (17.83)	65.00	
Lazaro‐Garcia 2024 Spain [67]	Hospital	Parallel group	Anxiety (HADS‐A), depression (HADS‐D), QoL (FACT‐G)	Acute myeloid leukemia	NR	Pre‐recorded music listening	40	53.01 (NR)	45.00	19–42 days, 19‐42 sessions
						Control	31	53.89 (NR)	41.94	
Li 2012 China [68]	Hospital	Parallel group	Anxiety (STAI)	Breast	NR	Pre‐recorded music listening	60	44.88 (9.37)	100	51.4 days, ∼100 sessions
						Control	60	45.13 (9.48)	100	
Liao 2013 China [69]	Hospital	Parallel group	QoL (HQLI‐R)	Any	IV	Chinese‐medicine five‐element music listening	66	63.09 (12.46)	53.03	3 weeks, 15 sessions
						Western music listening	63	63.52 (14.65)	49.21	
						Control	31	62.68 (13.87)	54.84	
Lima 2020 Brazil [34]	Hospital	Parallel group	Anxiety (Bai), depression (BDI‐II), QoL (WHOQOL‐BREF)	Breast	NR	Pre‐recorded music listening	16	49.5 (10.65)	100	4–6 weeks^c^, 3 sessions
						Control (self‐relaxation without music)	17	50.76 (9.45)	100	
Lin 2011 Taiwan [70]	Outpatient chemotherapy unit	Parallel group	Anxiety (Chinese STAI)	Any	NR	Music therapy	34	50.2 (13.5)	61.76	1 day, 1 session
						Verbal relaxation	30	54.3 (9.4)	70.00	
						Control	34	54.3 (13.6)	67.65	
McCabe 2013 Ireland [71]	Stem cell transplant center	Parallel group	Anxiety/Depression (HADS)	Hematologic	NR	Art therapy (Open Window)	96	NR (NR)	39.58	1+ week, 3+ sessions
						Control	103	NR (NR)	41.75	
Mollaoglu 2023 Turkey [72]	Hospital	Parallel group	Anxiety (Bai), QoL (FACT‐G)	Breast	I‐III	Marbling + Ney music	30	NR (NR)	100	5 weeks, 10 sessions
						Control	30	NR (NR)	100	
Mondanaro 2021 United States [73]	Hospital	Parallel group	Anxiety/Depression (HADS)	Lung, breast, or gastrointestinal	NR	Music therapy	43	55 (13.05)	76.74	1–3 months, 3 sessions
						Control	44	53.41 (11.94)	79.55	
Monti 2006 United States [74]	NR	Parallel group	Anxiety (SCL‐90‐R‐A), depression (SCL‐90‐R‐D), QoL (SF‐36‐mental)	Any	NR	Mindfulness‐based art therapy	56	53.1 (12.4)	100	8 weeks, 8 sessions
						Control	55	54.1 (10.7)	100	
Monti 2012 United States [75]	NR	Parallel group	Anxiety (SCL‐90‐R‐A)	Breast	NR	Mindfulness‐based art therapy	8	55 (5)	100	8 weeks, 8 sessions
						Control	10	56 (7)	100	
Monti 2013 United States [76]	NR	Parallel group	Anxiety (SCL‐90‐R‐A), depression (SCL‐90‐R‐D), QoL (SF‐36‐mental)	Breast	0‐IV	Mindfulness‐based art therapy	98	56.9 (NR)	100	8 weeks, 8 sessions
						Breast cancer support group	93	56.4 (NR)	100	
						Control	44	57.4 (NR)	100	
Mou 2020 China [77]	Cancer center	Parallel group	Anxiety (NVAAS)	Lung	NR	Pre‐recorded music listening	150	56.99 (9.84)	20.67	1 day, 1 session
						Control	150	57.63 (10.38)	18.67	
O'steen 2020 China [78]	University radiation therapy center	Parallel group	Anxiety (STAI)	Any	NR	Pre‐recorded + live music listening	51	63^a^ (NR)	100	1 day, 1 session
						Control	51	62^a^ (NR)	100	
Puig 2006 United States [79]	NR	Parallel group	Anxiety (POMS‐tension), depression (POMS‐depression)	Breast	I‐II	Creative arts therapy (meditation + poetry)	20	NR (NR)	100	4 weeks, 4 sessions
						Control	19	NR (NR)	100	
Rabinowitch 2023 Israel [35]	Online	Parallel group	Anxiety (STAI‐6)	Any	NR	Music therapy	14	NR (NR)	NR	1 day, 1 session
						Control (meditation)	16	NR (NR)	NR	
Raglio 2021 Italy [80]	Cancer center	Parallel group	Anxiety (STAI)	Breast	NR	Individualized pre‐recorded music listening	20	54.5^a^ (NR)	100	2 weeks, 6 sessions
						Algorithm‐based pre‐recorded music listening	20	58^a^ (NR)	100	
						Control	20	59^a^ (NR)	100	
Romito 2013 Italy [81]	Outpatients in cancer research center	Parallel group	Anxiety (emotion thermometer Tool‐A), depression (emotion thermometer Tool‐D)	Breast	NR	Music therapy and emotional expression	31	54.14 (NR)	100	1 day, 1 session
						Control	31	54.21 (NR)	100	
Rossetti 2017 United States [82]	Cancer center	Parallel group	Anxiety (STAI)	Breast, head and neck	NR	Music therapy	39	58^a^ (NR)	61.54	1 day, 1 session
						Control	39	60^a^ (NR)	69.23	
Sahan 2024 Turkey [83]	Hospital	Parallel group	Anxiety (STAI)	Hematologic	NR	Pre‐recorded music listening	32	50.7 (13.89)	53.13	1 day, 1 session
						Control	32	52.34 (14.67)	59.38	
Svensk 2009 Sweden [84]	Hospital	Parallel group	QoL (WHOQOL‐BREF)	Breast	Non‐metastatic	Art therapy	20	59.5^a^ (NR)	100	5 weeks, 5 sessions
						Control	21	55^a^ (NR)	100	
Tang 2021 China [85]	Cancer hospital	Parallel group	Anxiety (SAS)	Small cell lung carcinoma	NR	Music therapy	50	54.28 (5.59)	38.00	1 day, 1 session
						Control	50	54.28 (5.18)	40.00	
Tanriverdi 2023 Turkey [86]	Hospital	Parallel group	Anxiety (STAI), depression (BDI)	Colorectal	II‐III	Pre‐recorded music listening	32	51^a^ (NR)	40.63	2 days, 6 sessions
						Control	30	53^a^ (NR)	40.00	
Thyme 2009 Sweden [87]	Hospital	Parallel group	Anxiety (SCL‐90‐A), depression (SCL‐90‐D)	Breast	Non‐metastatic	Art therapy	20	59.5^a^ (NR)	100	5 weeks, 5 sessions
						Control	21	55^a^ (NR)	100	
Tollabzadeh 2023 Iran [88]	Hospital	Parallel group	Anxiety (Bai)	Any	NR	Pre‐recorded music listening	25	47.74 (13.96)	84.00	8 weeks, 16 sessions
						Control (counseling)	29	41.14 (9.87)	86.21	
Tuinmann 2017 Germany [89]	University cancer center	Parallel group	Anxiety (HADS‐A), depression (HADS‐D), QoL (EORTC‐QLQ‐C30)	Non‐Hodgkin and Hodgkin lymphoma, multiple myeloma, testicular, or leukemia	NR	Music therapy	33	50.7 (14.6)	39.39	4 weeks, 8 sessions
						Control	33	50.5 (15.4)	33.33	
UnalToprak 2024 Turkey [90]	Hospital	Parallel group	Anxiety (HADS‐A), depression (HADS‐D)	Gynecological	NR	Pre‐recorded music listening	30	59 (12.28)	100	1 day, 1 session
						Control	25	55.2 (10.54)	100	
Vachiramon 2013 United States [91]	Dermatologic surgery clinic	Parallel group	Anxiety (STAI, VAS)	Skin	NR	Pre‐recorded music listening	50	62.6 (14.3)	32.00	1 day, 1 session
						Control	50	66 (13.9)	34.00	
Yang 2024 Taiwan [92]	Hospital	Parallel group	Anxiety (Bai‐C)	Any	NR	Pre‐recorded music listening	50	59.9 (9.6)	50.00	10 days, 10 sessions
						Control	50	60.3 (11.3)	54.00	
Yates 2015 United States [93]	Post‐surgical oncology unit	Parallel group	Anxiety (QMS‐A), depression (QMS‐D)	Any	NR	Live music listening	13	57.73 (10.6)	84.62	1 day, 1 session
						Control	13	57.45 (14.24)	84.62	
Yildirim 2024 Turkey [94]	Hospital	Parallel group	Anxiety (STAI), depression (BDI)	Any	NR	Mindfulness‐based breathing + music therapy (listening)	60	57.85 (11.02)	36.67	10 days, 3 sessions
						Control	60	58.36 (11.09)	38.30	
Zengin 2013 Turkey [95]	Emergency department	Parallel group	Anxiety (STAI)	Any requiring port catheter placement	NR	Pre‐recorded music listening	50	49 (15.58)	44.00	1 day, 1 session
						Control	50	50.74 (14.01)	52.00	
Zhou 2011 China [96]	Hospital	Parallel group	Depression (ZSDS)	Breast	NR	Pre‐recorded music listening	60	44.88 (9.37)	100	51.4 days, ∼100 sessions
						Control	60	45.13 (9.48)	100	

^a^ Median age.

^b^ Mean age of all participants from all groups.

^c^ Estimated from 2 cycles of chemotherapy.

Abbreviations: BAI (C) = Beck Anxiety Inventory (Chinese), BDI = Beck Depression Inventory, CES‐D = Center for Epidemiologic Studies Depression Scale, EORTC‐QLQ‐C30 = European Organisation for Research and Treatment of Cancer Quality of Life Questionnaire Core 30, FACT (G/B) = Functional Assessment of Cancer Therapy (General/Breast), HADS (A/D) = Hospital Anxiety and Depression Scale (Anxiety/Depression), HAMD‐24 = Hamilton Depression Rating Scale 24‐item, HQLI‐R = Hospice Quality of Life Index‐Revised, NVAAS = Numeric Visual Analog Anxiety Scale, PAI (A/D) = Personality Assessment Inventory (Anxiety/Depression), POMS (A/D) = Profile of Mood States (Anxiety/Depression), PROMIS = Patient‐Reported Outcomes Measurement Information System, QMS (A/D) = 12‐item Quick Mood Scale (Anxiety/Depression), QoL = Quality of Life, SAI = State Anxiety Scale, SAS = Zung Self‐Rating Anxiety Scale, SCL‐90‐R (A/D) = Symptom Checklist‐90‐Revised (Anxiety/Depression), SF‐36 = 36‐item Short‐Form Health Survey, STAI (Y/6) = State‐Trait Anxiety Inventory (20‐item/6‐item scale), VAS = Visual Analogue Scale, WHOQOL‐BREF = World Health Organization Quality of Life ‐ Abbreviated, ZSDS = Zung Self‐Rating Depression Scale.

#### 3.1.2 Risk of Bias

**FIGURE 2:**
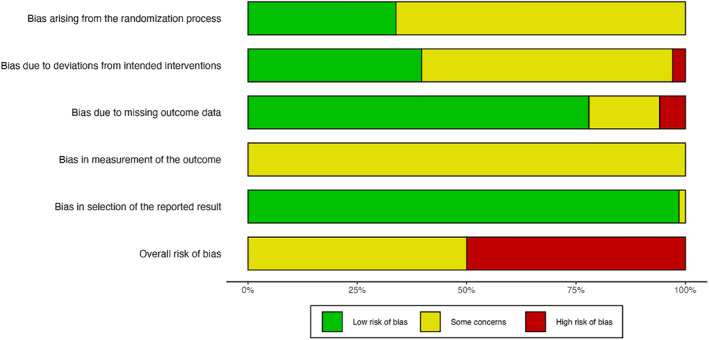
Cochrane risk of bias 2.0 summary plot.

We determined all studies to be of either moderate or high risk of bias, mainly due to the lack of participant blinding which was not possible given the nature of the intervention. This raised concerns around deviations from intended interventions and measurement of the outcomes, which were largely participant‐reported. We also found many studies to have concerns regarding the randomization process, most commonly due to lack of allocation concealment. A summary of the risk of bias assessments can be found in Figure 2, and a complete analysis of each individual study's assessments in Supporting Information S2: Appendix A2.

#### 3.1.3 Anxiety

For the primary analyses, we found CATs to have a positive effect on anxiety at the immediate (0–6 days) (SMD −0.62 [95% CI −1.01, −0.24]), 4–6 weeks (−1.21 [−2.08, −0.34]), and 2–3 months intervals (−1.19 [−2.14, −0.24]). No significant effect was observed at 1–3 weeks (−0.62 [−1.26, 0.02]) or 4–6 months (−0.85 [−1.92, 0.23]). The results of the meta‐analyses are presented in Table 2.

**TABLE 2:** Summary of standardized mean differences (SMD) from meta‐analyses.

	< 1 week			1–3 weeks			4–6 weeks			2–3 months			4–6 months		
Outcome	*k*	Pooled effect (SMD)	*I* ^2^ *(%)*	*k*	Pooled effect (SMD)	*I* ^2^ *(%)*	*k*	Pooled effect (SMD)	*I* ^2^ *(%)*	*k*	Pooled effect (SMD)	*I* ^2^ *(%)*	*k*	Pooled effect (SMD)	*I* ^2^ *(%)*
Anxiety	22	−0.62 [−1.01, −0.24]	93.6	5	−0.62 [−1.26, 0.02]	92.5	8	−1.21 [−2.08, −0.34]	94.8	7	−1.19 [−2.14, −0.24]	93.7	3	−0.85 [−1.92, 0.23]	89.0
Depression	7	−0.15 [−0.47, 0.18]	64.7	5	−0.44 [−0.87, −0.00]	85.0	5	−1.14 [−1.76, −0.52]	81.4	2	−0.95 [−3.34, 1.43]	93.6	2	−0.18 [−1.19, 0.83]	81.1
Quality of life	—	—	—	4	0.65 [0.05, 1.25]	88.7	4	1.17 [0.02, 2.32]	96.2	7	1.42 [0.55, 2.29]	94.7	4	0.42 [0.04, 0.80]	69.0

*Note:* For SMD, < 0 indicates improvement (reduction) in Anxiety and Depression and > 0 indicates improvement (increase) in Quality of Life.

Abbreviation: *k* = number of studies.

Subgroup analyses revealed a greater reduction in anxiety at 1–3 weeks for interventions performed in inpatient (−1.11 [−1.62, −0.59]) compared to outpatient settings (0.04 [−0.36, 0.45]) [47]. Additionally, we found a subgroup effect by region/continent at the 4–6 months interval, with anxiety improving more in West Asian (−1.82 [−2.43, −1.21]) than North American studies (0.04 [−0.56, 0.65]). No subgroup effects were observed for intervention type or session frequency. All forest plots can be found in Supporting Information S3: Appendix A3.

#### 3.1.4 Depression

In the primary analyses, we found CATs to have a positive effect on depression at the 1–3 weeks (−0.44 [−0.87, −0.00]) and 4–6 weeks intervals (−1.14 [−1.76, −0.05]). The results of the meta‐analyses are presented in Table 2.

We found evidence of a subgroup effect by intervention type at 4–6 weeks, with music therapy demonstrating a greater effect (−1.60 [−2.02, −1.18]) than art therapy (−0.19 [−0.80, 0.42]). We also observed an effect of intervention setting, with inpatient interventions demonstrating a superior effect to outpatient interventions at the immediate time interval (−0.30 [−0.54, −0.06] vs. 0.43 [0.03, 0.82]) and at 1–3 weeks (−0.77 [−1.12, −0.41] vs. 0.13 [−0.27, 0.54]). Subgroup analyses by region/continent also revealed a subgroup effect at 4–6 weeks with greater improvements observed among East Asian studies (−1.93 [−2.40, −1.47]) compared to those conducted in Europe (−0.19 [−0.80, 0.42]) and North America (−0.82 [−1.47, −0.16]). No subgroup effects were observed for session frequency. Forest plots can be found in Supporting Information S3: Appendix A3.

#### 3.1.5 Quality of Life

In the primary analyses, we found CATs to have a positive effect on quality of life at all four reported time‐intervals: 1–3 weeks (0.65 [0.05, 1.25]), 4–6 weeks (1.17 [0.02, 2.32]), 2–3 months (1.42 [0.55, 2.29]), and 4–6 months (0.42 [0.04, 0.80]). The results of the meta‐analyses are presented in Table 2.

We observed a subgroup effect of region/continent at 4–6 weeks and 2–3 months, favoring West Asian studies at both timepoints. At 4–6 weeks, studies conducted in West Asia (4.28 [3.34, 5.22]) produced a greater effect than East Asian (−0.11 [−0.46, 0.23]), European (0.12 [−0.13, 0.38]), and South American studies (0.79 [0.08, 1.50]). At 2–3 months, West Asian studies (1.42 [0.55, 2.29]) produced a greater effect than East Asian (0.74 [0.01, 1.46]), European (0.61 [−0.01, 1.24]), and North American studies (0.38 [−0.87, 1.64]). No subgroup effects were observed for intervention type or setting. We were unable to perform subgroup analyses by session frequency because there were insufficient studies within each subgroup that reported quality of life at comparable time points. Forest plots can be found in Appendix A3.

#### 3.1.6 Publication Bias

The only meta‐analysis with more than 10 studies was anxiety at the immediate time interval, in which we found no evidence of publication bias upon visual inspection of the funnel plot. Funnel plot can be found in Appendix A3.

#### 3.1.7 Qualitative Results

Twenty‐two studies were not included in the meta‐analyses due to lack of reporting of the necessary statistics (i.e., sample sizes, central tendencies, and/or measure of dispersion). Of these, 21 reported on anxiety, 11 on depression, and 5 on quality of life. CATs were associated with a significant reduction in anxiety in 66.7% of studies (*n* = 14); for depression, CATs were associated with a significant reduction in 45.5% of studies (*n* = 5); for quality of life, CATs were associated with a significant improvement in quality of life in 40% of studies (*n* = 2). A table summarizing the results of the qualitative analysis can be found in Table 3.

**TABLE 3:** Summary of qualitative results.

Study	Anxiety	Depression	Quality of life
Alam 2016	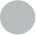	—	—
Alcântara‐silva 2018	—	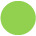	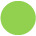
Bradt 2024	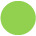	—	—
Bro 2019	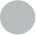	—	—
Bulfone 2009	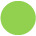	—	—
Cassileth 2003	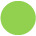	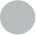	—
Chen 2013	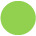	—	—
Chen 2018	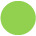	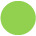	—
Clark 2006	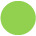	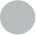	—
Du 2024	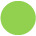	—	—
Firmeza 2017	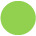	—	—
Giordano 2023	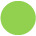	—	—
Lazaro‐Garcia 2024	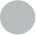	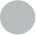	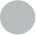
McCabe 2013	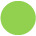	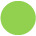	—
Mondanaro 2021	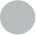	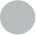	—
Monti 2006	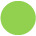	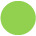	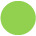
Monti 2013	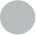	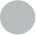	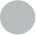
O'steen 2020	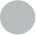	—	—
Raglio 2021	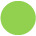	—	—
Romito 2013	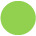	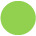	—
Rossetti 2017	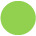	—	—
Tuinmann 2017	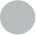	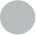	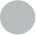

Abbreviations: 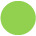 = significant improvement (*p* < 0.05) with CAT, 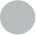 = no change (*p* > 0.05) with CAT, 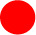 = significant negative effect (*p* < 0.05) with CAT.

#### 3.1.8 GRADE Assessment

The GRADE assessment of the certainty of the evidence revealed low‐to‐very low certainty evidence for all of the outcomes, mostly due to the substantial risk of bias among included studies and unexplained heterogeneity. The GRADE assessments are summarized in Table 4.

**TABLE 4:** GRADE evidence profile.

Outcome	Timeframe	Studies (*n*)	Certainty assessment					Certainty of evidence rating & relative effect size
			Risk of bias	Inconsistency	Indirectness	Imprecision	Publication bias	
Anxiety	Immediate	22 (1967)	Some limitations	Some limitations	No limitations	No limitations	No limitations	Low
								SMD −0.62 [−1.01, −0.24]
	1–3 weeks	5 (549)	Some limitations	Some limitations	No limitations	Some limitations	No limitations	Very low
								SMD −0.62 [−1.26, 0.02]
	4–6 weeks	8 (526)	Some limitations	Some limitations	No limitations	No limitations	No limitations	Low
								SMD −1.21 [−2.08, −0.34]
	2–3 months	7 (386)	Some limitations	Some limitations	No limitations	No limitations	No limitations	Low
								SMD −1.19 [−2.14, −0.24]
	4–6 months	3 (143)	Some limitations	Some limitations	No limitations	Some limitations	No limitations	Very low
								SMD −0.85 [−1.92, 0.23]
Depression	Immediate	7 (465)	Some limitations	Some limitations	No limitations	Some limitations	No limitations	Very low
								SMD −0.15 [−0.47, 0.18]
	1–3 weeks	5 (569)	Some limitations	Some limitations	No limitations	No limitations	No limitations	Low
								SMD −0.44 [−0.87, 0.00]
	4–6 weeks	5 (278)	Some limitations	Some limitations	No limitations	No limitations	No limitations	Low
								SMD −1.14 [−1.76, −0.52]
	2–3 months	2 (66)	Some limitations	Some limitations	No limitations	Some limitations	No limitations	Very low
								SMD −0.95 [−3.34, 1.43]
	4–6 months	2 (83)	Some limitations	Some limitations	No limitations	Some limitations	No limitations	Very low
								SMD −0.18 [−1.19, 0.83]
Quality of life	1–3 weeks	4 (430)	Some limitations	Some limitations	No limitations	No limitations	No limitations	Low
								SMD 0.65 [0.05, 1.25]
	4–6 weeks	4 (460)	Some limitations	Some limitations	No limitations	No limitations	No limitations	Low
								SMD 1.17 [0.02, 2.32]
	2–3 months	7 (540)	Some limitations	Some limitations	No limitations	No limitations	No limitations	Low
								SMD 1.42 [0.55, 2.29]
	4–6 months	4 (440)	Some limitations	Some limitations	No limitations	No limitations	No limitations	Low
								SMD 0.42 [0.04, 0.80]

*Note:* For SMD, < 0 indicates improvement (reduction) in Anxiety and Depression and > 0 indicates improvement (increase) in Quality of Life.

Abbreviation: SMD = standardized mean difference.

## 4 Discussion

The results of this systematic review and meta‐analyses of 67 randomized controlled trials suggest that creative arts therapies may be effective in improving psychological outcomes in cancer patients. In particular, our meta‐analyses found that creative arts therapies may reduce depression and anxiety for up to 6 weeks and 3 months post‐treatment, respectively, and improve quality of life up to 6 months post‐treatment. The qualitative analyses support these findings, particularly in the outcome of anxiety.

Our results, which found CATs to reduce anxiety and depression among cancer patients, are in alignment with previous evidence syntheses on the topic [17, 18, 20, 21, 97]. This effect is likely driven by the ability of CATs to induce relaxation and regulate the cognitive and emotional systems [98]. While the exact mechanism behind this remains unclear, preliminary evidence suggests that creative arts consistently activate neural circuits involved in emotional regulation, including the medial prefrontal cortex and amygdala; this may indicate a shared cognitive pathway between creative expression and emotional processing [99]. We also found that CATs were most effective in treating anxiety and depression when applied in an inpatient setting compared to an outpatient setting. Given that hospitalized cancer patients are likely to have a higher baseline level of anxiety and depression [100], it may be that psychological relief is simply most needed in the hospital.

Interestingly, we found that music therapy had the largest effect on depression, although it is important to note that this was observed only at the 4–6 weeks timepoint. There is substantial evidence supporting the ability of music to regulate mood [101, 102]. These benefits are likely due to music's ability to engage interconnected brain regions involved in emotion and reward processing, including the amygdala, hippocampus, nucleus accumbens, and prefrontal cortex; ultimately, this activation is thought to improve emotional regulation, reduce stress, and enhance feelings of pleasure [103]. Moreover, music is intrinsically enjoyable and facilitates verbal and non‐verbal expression of emotion [104], which likely further contributes to its benefits. While CATs broadly appear to reduce depression, music may be the most effective way of providing system regulation in cancer patients.

We found CATs to improve quality of life up to 6 months post‐treatment. Previous reviews report mixed results: while most report an improvement in overall quality of life [17, 19, 20, 21, 97], some report no benefit at all or only in specific sub‐dimensions like social or physical quality of life [18, 19, 22]. In addition to the psychophysiological benefits described previously, CATs have been said to promote a sense of personal agency, hope, personal connection, emotional awareness, and flow state [104] that may not only induce improvements in clinical outcomes, but also provide existential relief and a sense of meaning for cancer patients.

The only subgroup effect consistently observed across all three outcomes was the effect of region. We found that studies conducted in Asian countries, and thus Asian populations, were associated with a higher effect size. It is first important to acknowledge the risk of bias and large effect sizes observed in some of the included studies; effect estimates should be interpreted cautiously. That being said, these geographic differences may be a result of cultural differences between Western and Asian societies: Asian cultures tend to be more collectively oriented, have traditional roots in mindfulness and acceptance‐based philosophies, and place less emphasis on emotional expression [105]. CATs typically encourage creative expression and self‐engagement, both of which also contribute to their therapeutic effect [104]. CATs may therefore be particularly effective in populations where emotional expression is not the norm and there is an established amenability to artistic or mindfulness interventions.

### 4.1 Clinical and Research Implications

Both our meta‐analyses and qualitative findings support the use of creative arts therapies as a potentially effective psychosocial intervention for improving anxiety, depression, and quality of life in cancer patients.

There may be greater clinical benefits of implementing CATs in the hospital compared to outpatient settings, particularly for anxiety and depression. However, this may owe to the higher baseline levels of anxiety and depression observed in hospitalized cancer patients [100], such that they are more likely to reap greater benefits from CATs. To explore this further, studies comparing the effect of CATs in hospitalized versus non‐hospitalized patients and controlling for baseline psychological symptom levels are of interest.

Compared to other intervention types, music‐based internventions may be the most effective in improving depression for cancer patients. These include music therapy sessions led by a registered music therapist (e.g., active “music‐making” interventions), but can also encompass lower‐cost alternatives such as listening to pre‐recorded music. For patients facing complex socioeconomic challenges, the latter may offer an affordable and accessible way to support mood regulation.

Across anxiety, depression, and quality of life, CATs appear to offer the greatest benefit in Asian countries. Clinicians working in Asian settings, or with Asian populations in other regions, may consider prioritizing CATs as a culturally responsive intervention. Additionally, this highlights the importance of tailoring psychosocial interventions to specific cultural contexts rather than adopting a one‐size‐fits‐all approach.

### 4.2 Strengths and Limitations

This study has many strengths. We conducted a quantitative and qualitative synthesis of 67 randomized trials on three clinically important psychological outcomes for cancer patients, utilizing the best practices in evidence synthesis (i.e., independent and in duplicate screening and extraction; risk of bias assessment; certainty of the evidence assessment). Our analyses were stratified by time‐point to reduce heterogeneity and provide important context on the longevity of effects from CATs in cancer patients. We also conducted several subgroup analyses of important factors that influence treatment effect, such as intervention type, session frequency, intervention setting, and region. These subgroup analyses help to identify optimal protocols.

This study was limited by our inclusion of English studies only; however, we identified 67 randomized trials and only excluded four studies due to non‐English language, which gives us confidence in the representativeness of our sample. Additionally, despite multiple subgroup analyses, we were unable to explain much of the heterogeneity observed in the primary analyses. Heterogeneity may also have been introduced by differences in measurement tools across studies. Although all tools used have established validity in oncology or psychosocial research, differences in sensitivity, specificity, scaling, and psychometric properties may have influenced the comparability of observed effects. Lastly, many studies were rated as having high risk of bias, which may have contributed to inflated effect estimates. Nevertheless, the observed effects of CATs were generally consistent in direction across studies and outcomes, suggesting an overall benefit despite limitations in study quality.In conjunction with the certainty of evidence ratings, the results of these meta‐analyses should be interpreted judiciously.

## 5 Conclusion

Creative arts therapies may reduce anxiety and depression and improve quality of life among cancer patients. Meta‐analyses suggest that reductions in depression may persist for up to 6 weeks post‐intervention, anxiety up to 3 months, and quality‐of‐life improvements up to 6 months. Music‐based interventions, including both therapist‐led sessions and lower‐cost listening interventions, may be particularly effective for improving depressive symptoms. Greater effects were observed in inpatient settings and in Asian countries, highlighting the importance of contextual and cultural considerations. Collectively, these findings support the integration of creative arts therapies into comprehensive cancer care to address anxiety, depression, and quality of life.

This review advances prior evidence syntheses by providing methodological elements that have been largely absent from earlier reviews, include a focused meta‐analysis of randomized controlled trials with stratification by follow‐up time points, subgroup analyses of potential sources of heterogeneity, and a certainty of evidence assessment using GRADE. At the same time, the findings highlight important limitations in the existing literature and underscore the need for high‐quality randomized controlled trials to strengthen the evidence base and inform clinical practice.

## Author Contributions

**Ashlyn S. L. Chou:** conceptualization, formal analysis, methodology, project administration, software, validation, visualization, writing – original draft, writing – review and editing. **Tyler McKechnie:** conceptualization, formal analysis, methodology, validation, visualization. **Vikram Arora:** formal analysis, software, validation, visualization. **Brianna DePestel:** conceptualization, methodology, software, validation. **Austine Wang:** conceptualization, methodology, software, validation. **Sameer Parpia:** methodology, supervision, validation, writing – review and editing. **Goran Calic:** supervision, validation, writing – review and editing. **Phillip Staibano:** supervision, validation, writing – review and editing. **Alexandra Derus:** writing – review and editing. **Akanksha Guleria:** writing – review and editing. **Mohit Bhandari:** conceptualization, formal analysis, methodology, project administration, supervision, validation, writing – review and editing. **Alex Thabane:** conceptualization, formal analysis, methodology, project administration, software, supervision, validation, visualization, writing – original draft, writing – review and editing.

## Ethics Statement

This review was exempt from ethical approval as it involved the analysis of published literature.

## Consent

This review has been registered under PROSPERO as “Effectiveness of creative arts therapies in improving anxiety, depression and quality of life in adult cancer patients: A systematic review and meta‐analysis” and registration number CRD420251087592. A protocol was not prepared.

## Conflicts of Interest

The authors declare no conflicts of interest.

## Supporting information


Supporting Information S1

Supporting Information S2

Supporting Information S3

## Data Availability

The data that support the findings of this study are available from the corresponding author upon reasonable request.
