# Supplementary material for: Creative Arts Therapy for Anxiety, Depression, and Quality of Life in Cancer Patients: A Systematic Review and Meta‐Analysis of Randomized Controlled Trials
Source: Psychooncology. 2026 Mar 19;35(3):e70425. doi: 10.1002/pon.70425 (PMC13000673; doi:10.1002/pon.70425)
Supplement: Supplementary file 1 — Supporting Information S1 [file PON-35-e70425-s003.docx]

**Appendix A1: Search strategies.**

APA PsycInfo <1806 to February Week 4 2025>

1 exp Neoplasms/ 70588

2 exp Oncology/ 7030

3 (cancer or tumor or tumour or oncolog* or malignan* or carcinoma or sarcoma or neoplasm).ti,ab. 100426

4 (visual art or art therapy or creative arts therapy or dance or drama or music therapy or movement therapy or dance therapy or drama therapy or creative arts or creative express*).ti,ab. 25999

5 exp creative arts therapy/ or movement therapy/ 15596

6 drama/ or music therapy/ 8920

7 randomized controlled trial.pt. or randomized.mp. or placebo.mp. 144089

8 exp Randomized Controlled Trials/ 1886

9 movement therapy/ 1685

10 1 or 2 or 3 105834

11 4 or 5 or 6 31051

12 7 or 8 144089

13 10 and 11 and 12 99

Ovid MEDLINE(R) <1996 to February 28, 2025>

1 exp Neoplasms/ 3002654

2 (cancer or tumor or tumour or oncolog* or malignan* or carcinoma or sarcoma).ti,ab. 3591959

3 (visual art or art therapy or creative arts therapy or dance or drama or music therapy or movement therapy or dance therapy or drama therapy or creative arts or creative express*).ti,ab. 14799

4 drama/ 1539

5 ((randomized controlled trial or controlled clinical trial).pt. or randomized.ab. or randomised.ab. or placebo.ab. or drug therapy.fs. or randomly.ab. or trial.ab. or groups.ab.) not (exp animals/ not humans.sh.) 4902676

6 exp Randomized Controlled Trials/ 190238

7 art therapy/ or dance therapy/ or music therapy/ 6361

8 exp Medical Oncology/ 31475

9 1 or 2 or 8 4316836

10 3 or 4 or 7 19243

11 5 or 6 4937641

12 9 and 10 and 11 446

Embase <1974 to 2025 February 28>

1 exp malignant neoplasm/ 4328168

2 oncology/ 160849

3 (cancer or tumor or tumour or oncolog* or malignan* or carcinoma or sarcoma).ti,ab. 5186784

4 (visual art or art therapy or creative arts therapy or dance or drama or music therapy or movement therapy or drama therapy or creative arts or creative express*).ti,ab. 19575

5 exp art therapy/ 5030

6 exp music therapy/ 9326

7 exp drama therapy/ 98

8 exp dance therapy/ 828

9 exp randomized controlled trial/ 808861

10 (Randomized controlled trial/ or Controlled clinical study/ or random$.ti,ab. or randomization/ or intermethod comparison/ or placebo.ti,ab. or (compare or compared or comparison).ti. or ((evaluated or evaluate or evaluating or assessed or assess) and (compare or compared or comparing or comparison)).ab. or (open adj label).ti,ab. or ((double or single or doubly or singly) adj (blind or blinded or blindly)).ti,ab. or double blind procedure/ or parallel group$1.ti,ab. or (crossover or cross over).ti,ab. or ((assign$ or match or matched or allocation) adj5 (alternate or group$1 or intervention$1 or patient$1 or subject$1 or participant$1)).ti,ab. or (assigned or allocated).ti,ab. or (controlled adj7 (study or design or trial)).ti,ab. or (volunteer or volunteers).ti,ab. or human experiment/ or trial.ti.) not (((random$ adj sampl$ adj7 ("cross section$" or questionnaire$1 or survey$ or database$1)).ti,ab. not (comparative study/ or controlled study/ or randomi?ed controlled.ti,ab. or randomly assigned.ti,ab.)) or (Cross-sectional study/ not (randomized controlled trial/ or controlled clinical study/ or controlled study/ or randomi?ed controlled.ti,ab. or control group$1.ti,ab.)) or (((case adj control$) and random$) not randomi?ed controlled).ti,ab. or (Systematic review not (trial or study)).ti. or (nonrandom$ not random$).ti,ab. or "Random field$".ti,ab. or (random cluster adj3 sampl$).ti,ab. or ((review.ab. and review.pt.) not trial.ti.) or ("we searched".ab. and (review.ti. or review.pt.)) or "update review".ab. or (databases adj4 searched).ab.) 6112796

11 1 or 2 or 3 6155642

12 4 or 5 or 6 or 7 or 8 26895

13 9 or 10 6130837

14 11 and 12 and 13 623
