# Supplementary figures and images for: Creative Arts Therapy for Anxiety, Depression, and Quality of Life in Cancer Patients: A Systematic Review and Meta‐Analysis of Randomized Controlled Trials
Source: Psychooncology. 2026 Mar 19;35(3):e70425. doi: 10.1002/pon.70425 (PMC13000673; doi:10.1002/pon.70425)

**Appendix A2: Complete risk of bias assessment.**
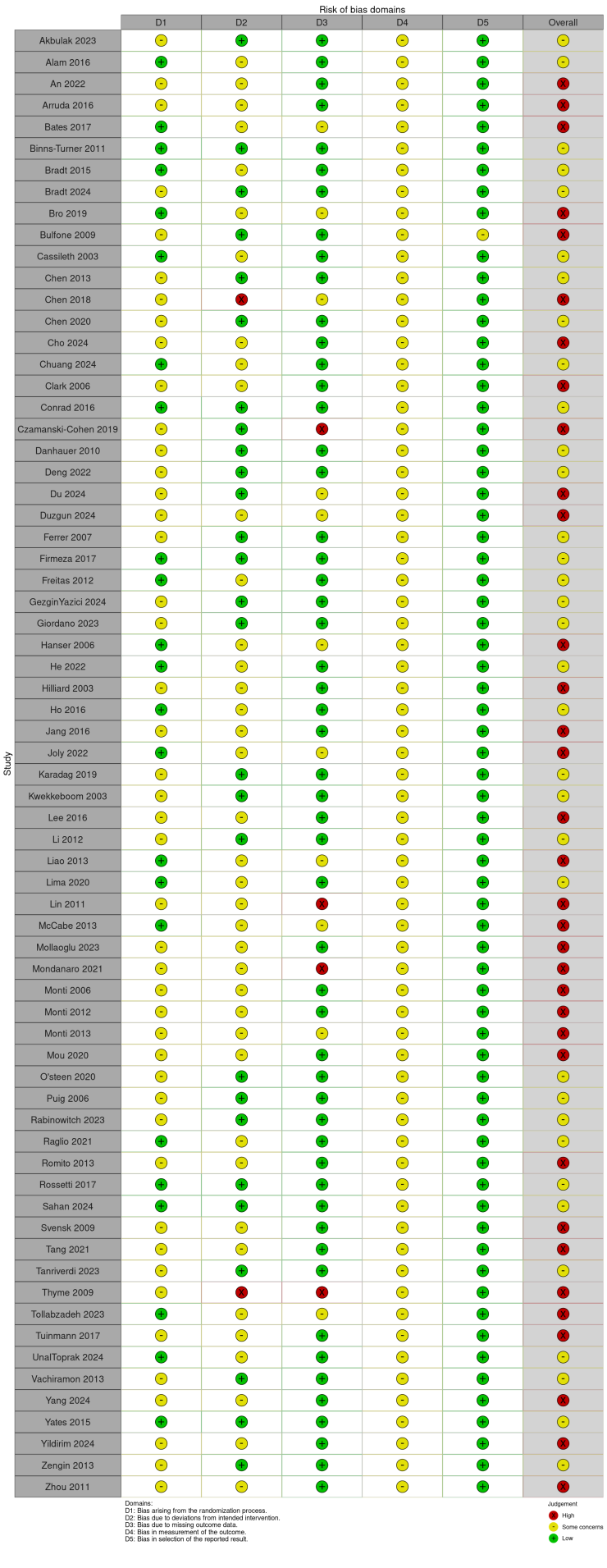

Supplement: Supplementary file 2 — Supporting Information S2 [file PON-35-e70425-s002.docx]
