# Supplementary material for: Creative Arts Therapy for Anxiety, Depression, and Quality of Life in Cancer Patients: A Systematic Review and Meta‐Analysis of Randomized Controlled Trials
Source: Psychooncology. 2026 Mar 19;35(3):e70425. doi: 10.1002/pon.70425 (PMC13000673; doi:10.1002/pon.70425)
Supplement: Supplementary file 3 — Supporting Information S3 [file PON-35-e70425-s001.docx]

**Appendix A3: Forest and funnel plots for primary and secondary meta-analyses.**

**PRIMARY ANALYSIS – ANXIETY**

*Anxiety*


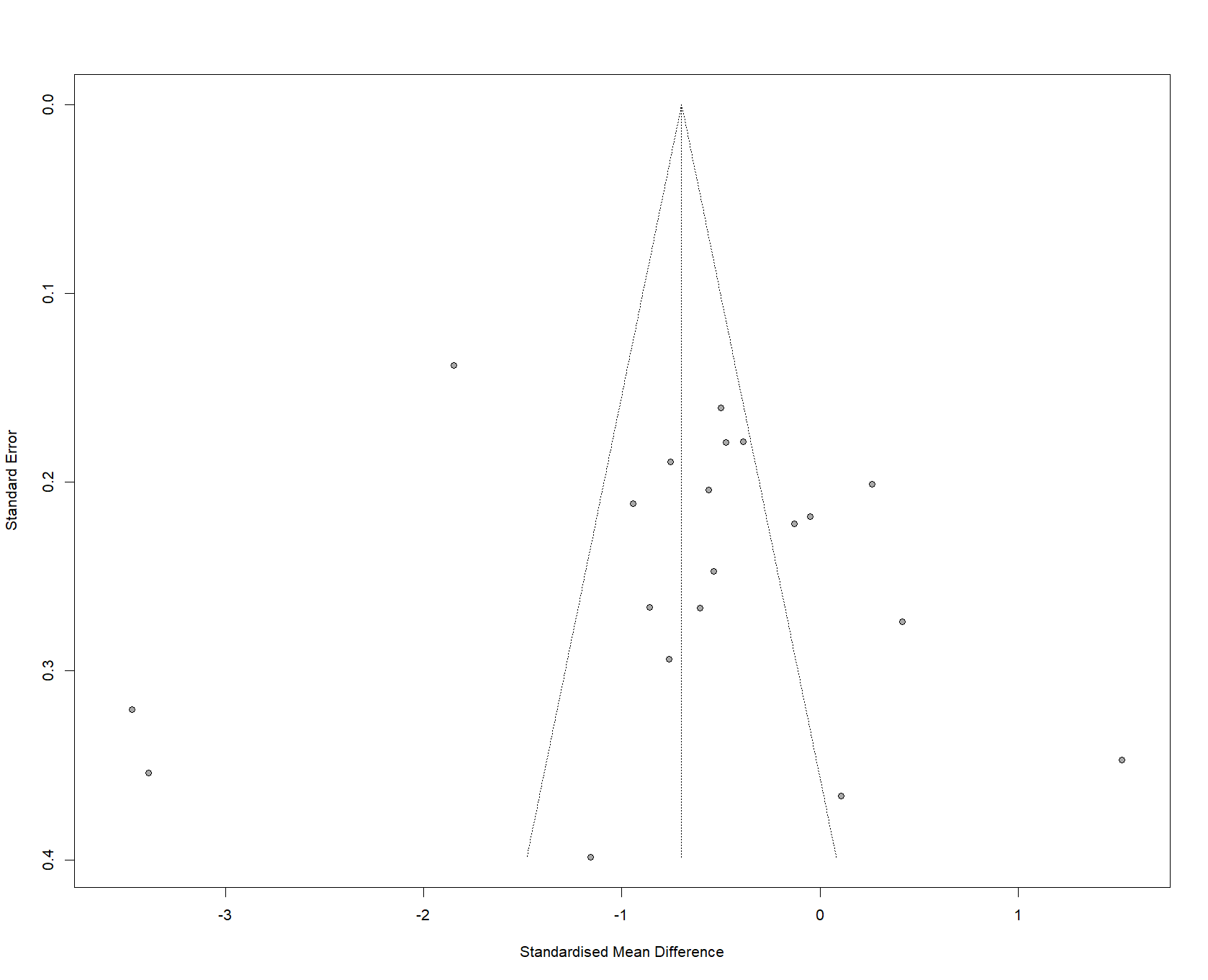
**Fig 1:** Funnel plot of studies reporting effect of CATs on anxiety within 1 week.

**
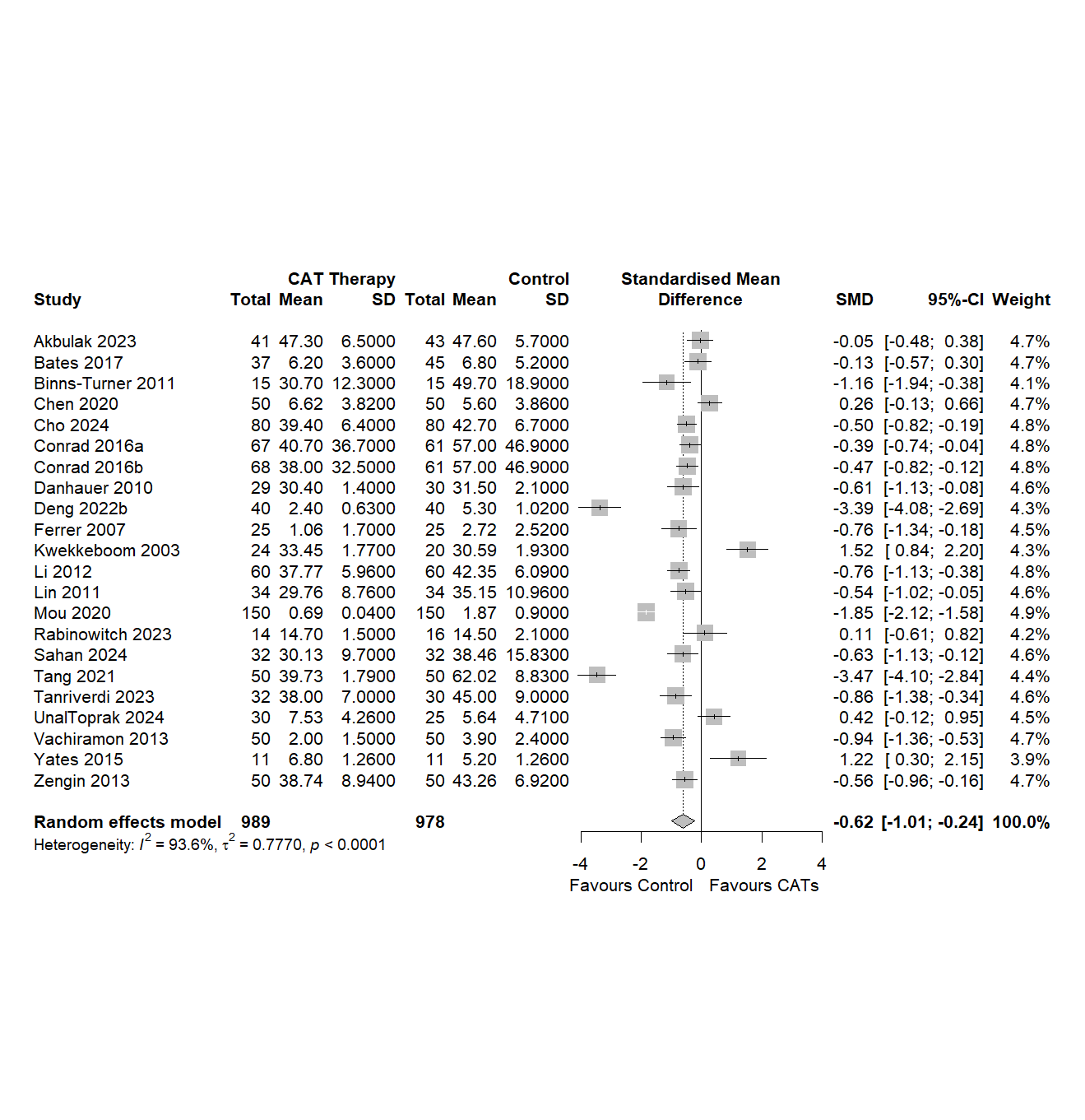
Fig 2:** Forest plot for effect of CATs on anxiety within 1 week.


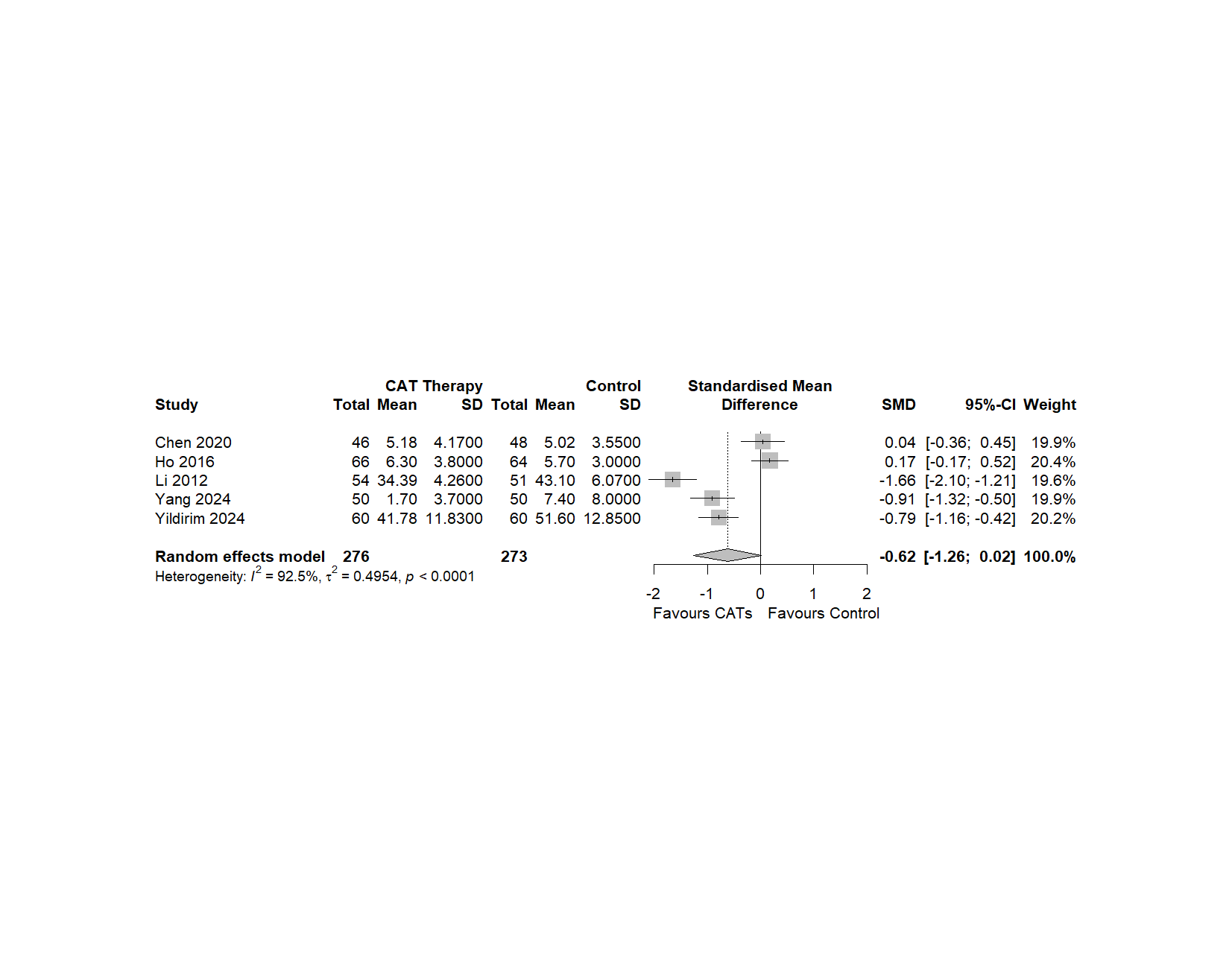
**Fig 3:** Forest plot for effect of CATs on anxiety between 1 to 3 weeks.


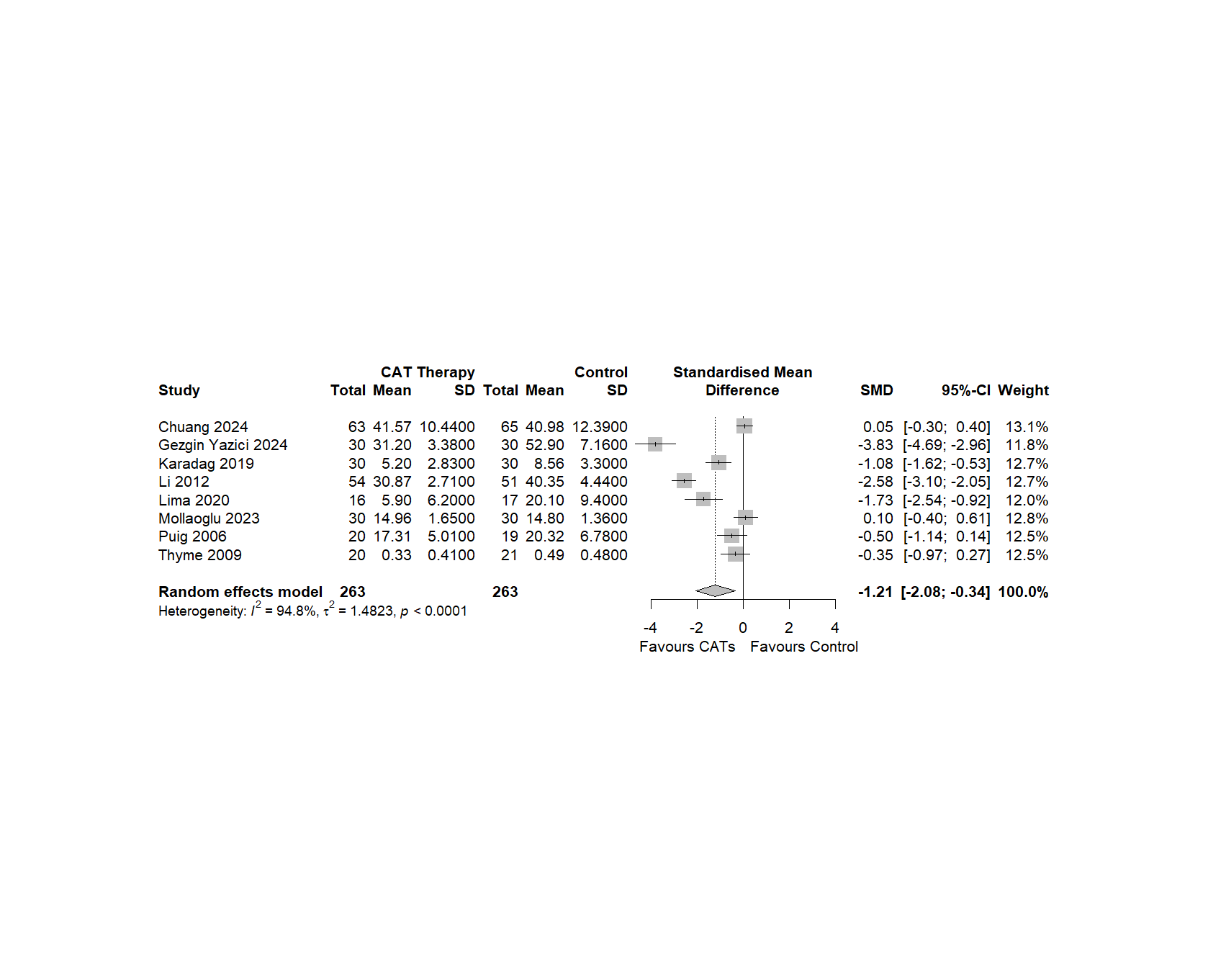
**Fig 4:** Forest plot for effect of CATs on anxiety between 4 to 6 weeks.


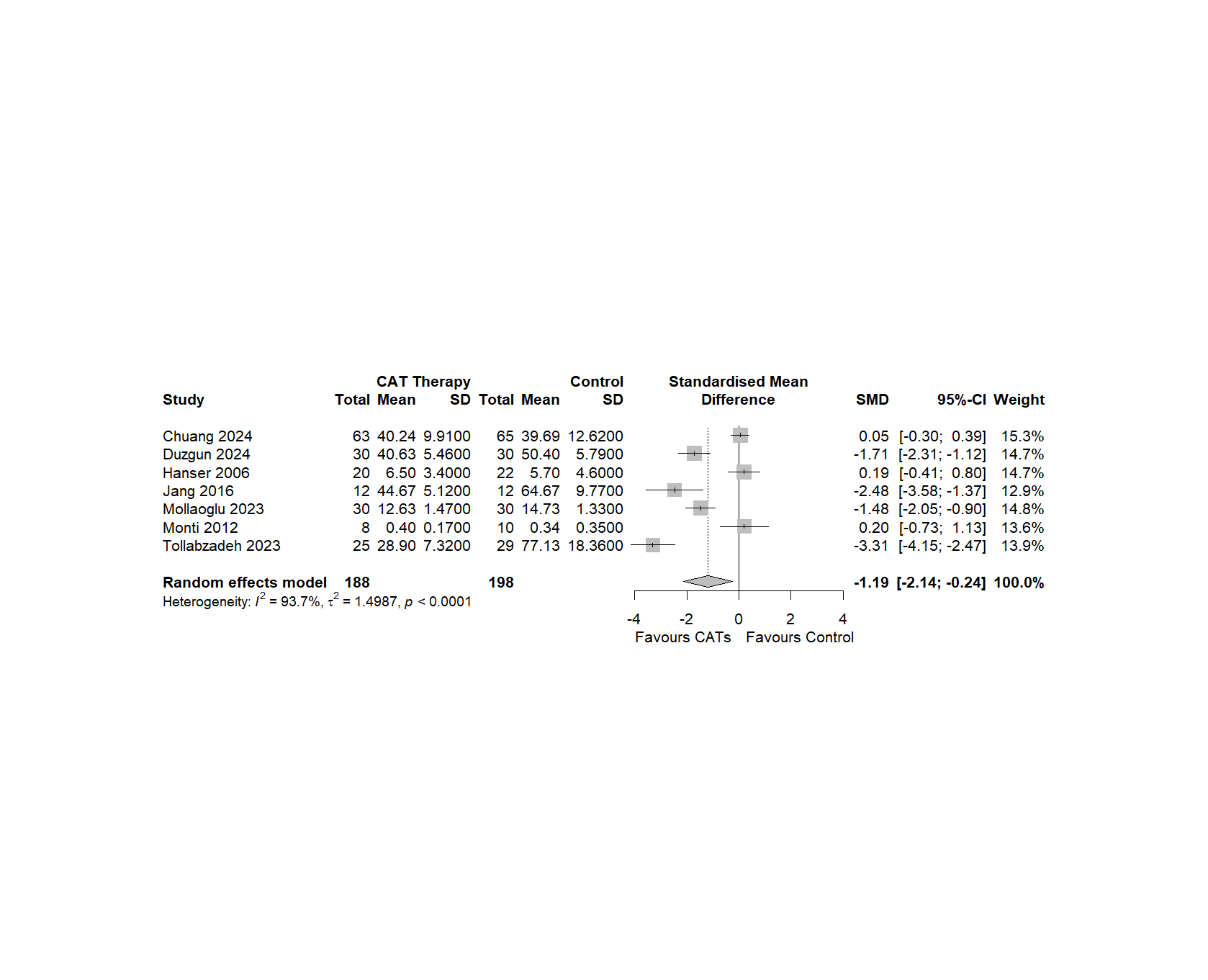
**Fig 5:** Forest plot for effect of CATs on anxiety between 2 to 3 months.


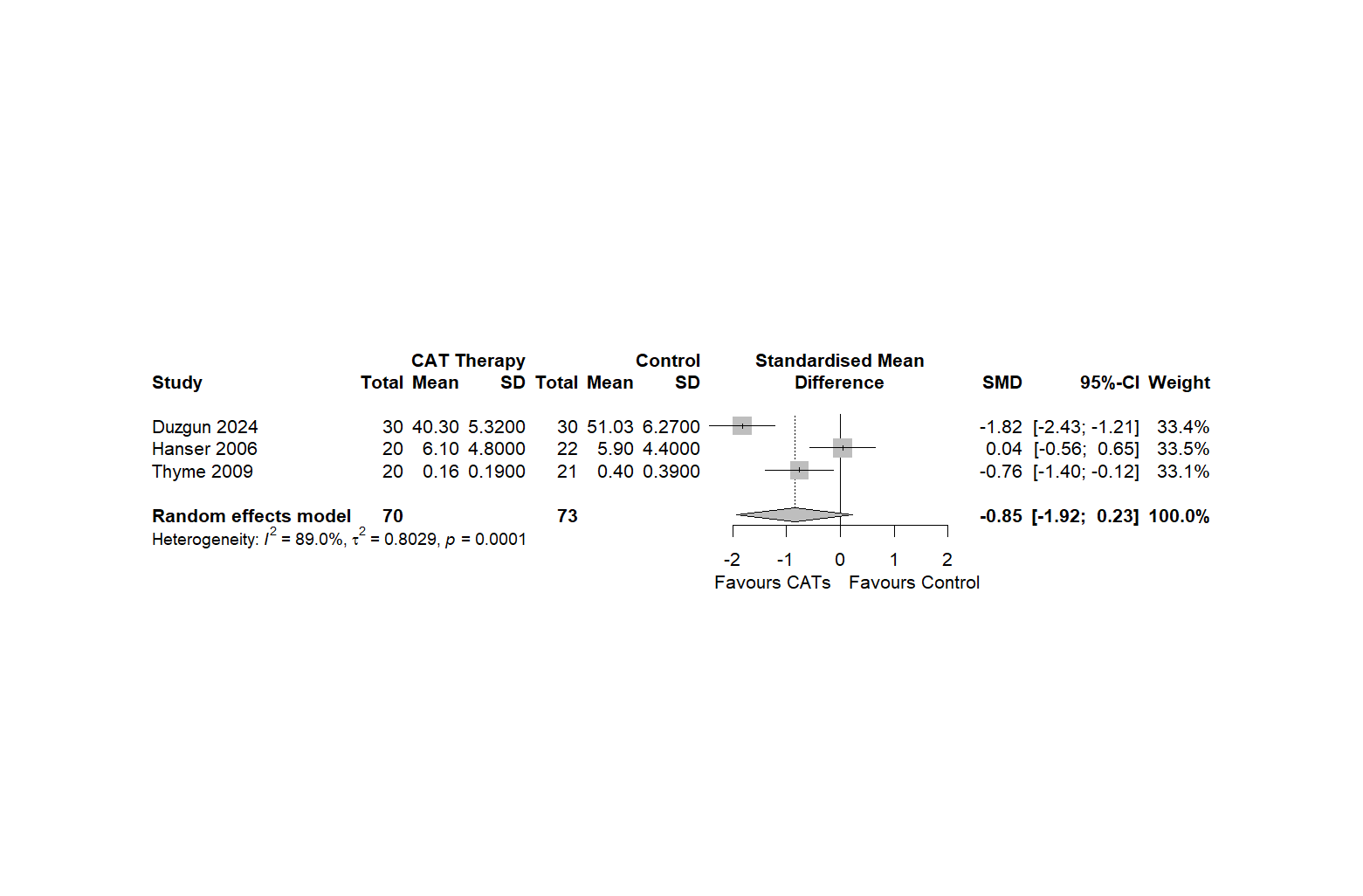
**Fig 6:** Forest plot for effect of CATs on anxiety between 4 to 6 months.

**PRIMARY ANALYSIS – DEPRESSION**

**
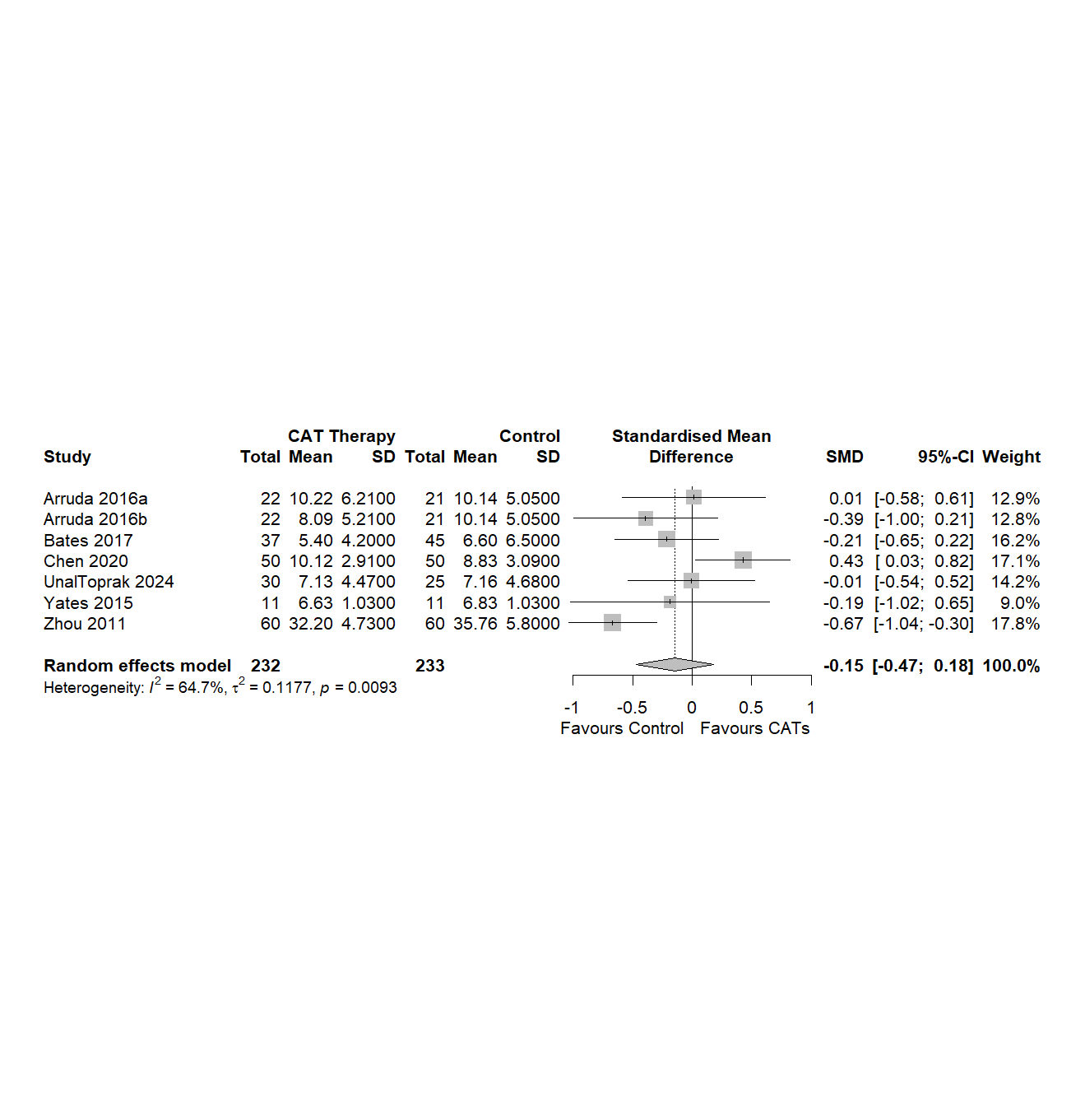
**

**Fig 7:** Forest plot for effect of CATs on depression within 1 week.


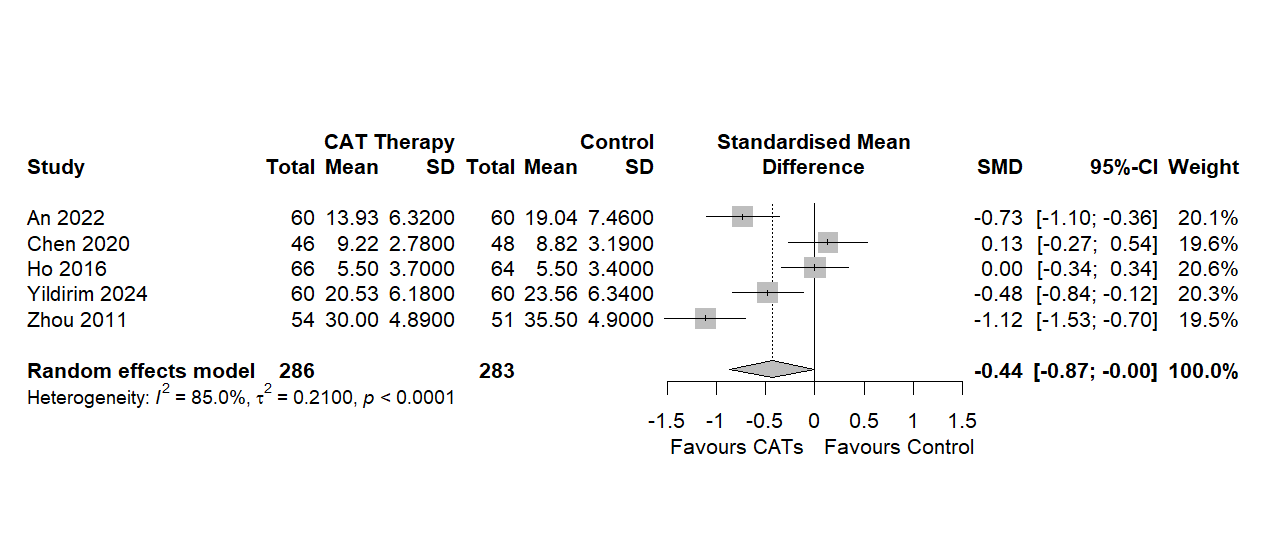


**Fig 8:** Forest plot for effect of CATs on depression between 1 to 3 weeks.


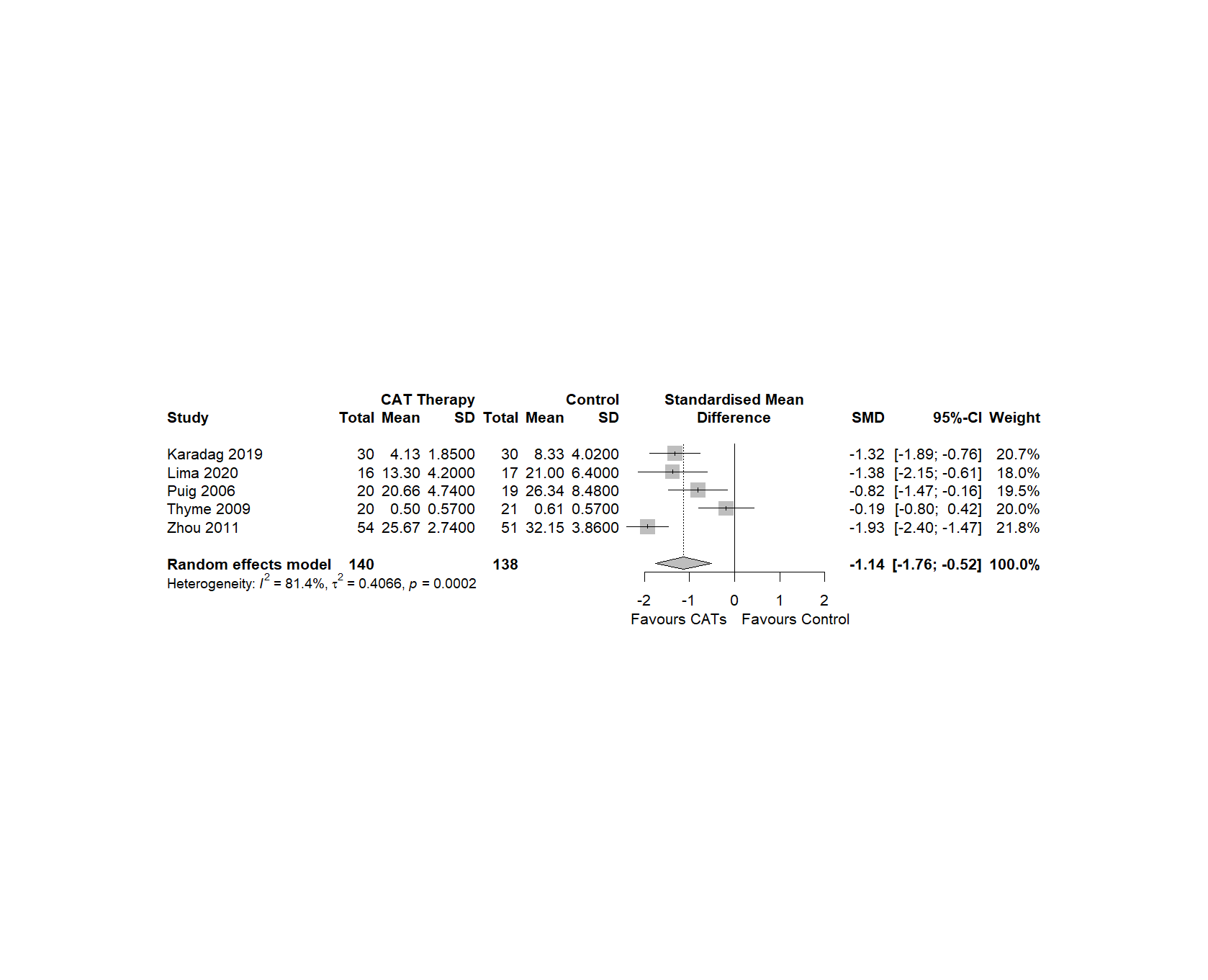


**Fig 9:** Forest plot for effect of CATs on depression between 4 to 6 weeks.


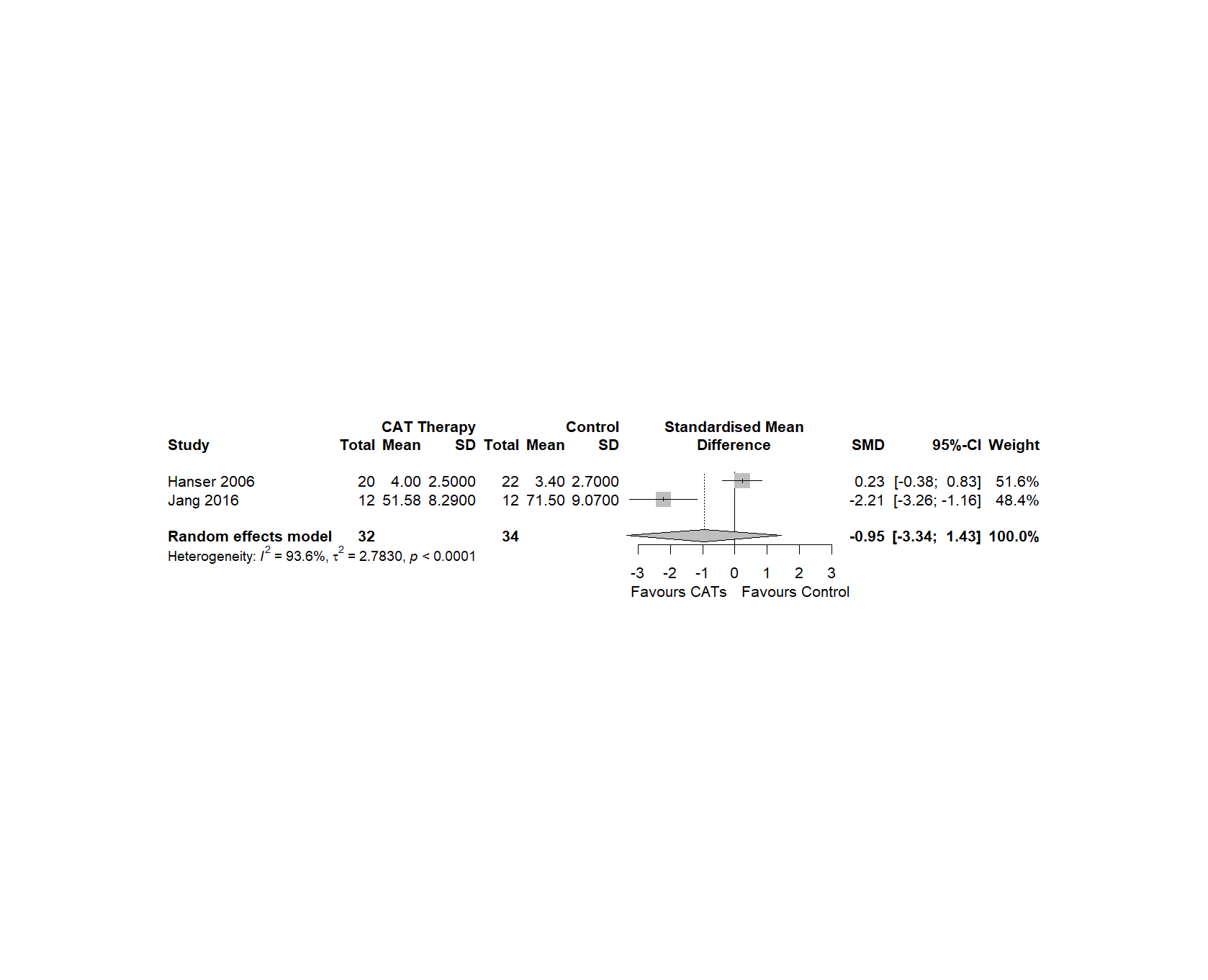


**Fig 10:** Forest plot for effect of CATs on depression between 2 to 3 months.


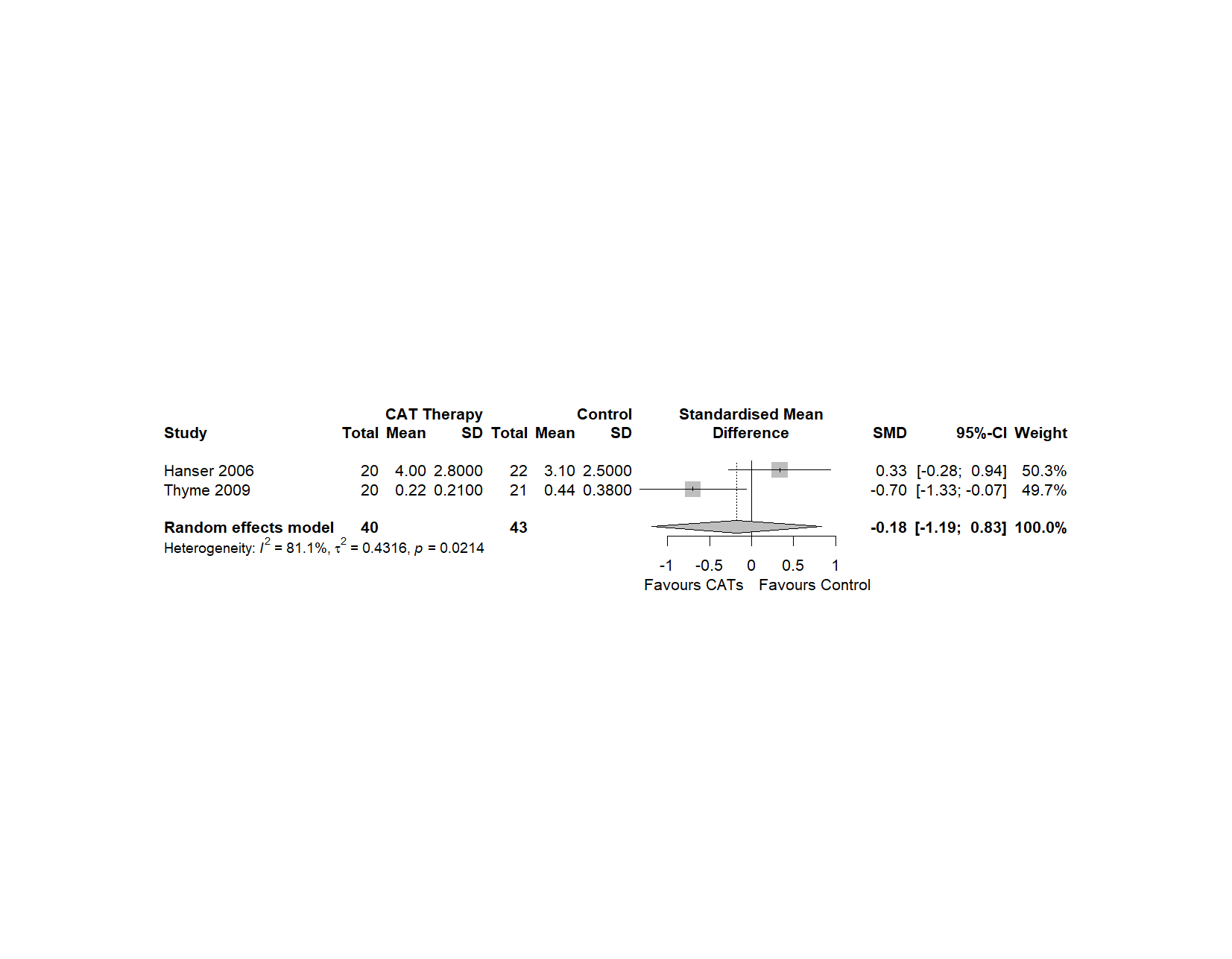


**Fig 11:** Forest plot for effect of CATs on depression between 4 to 6 months.

**PRIMARY ANALYSIS – QUALITY OF LIFE**

**
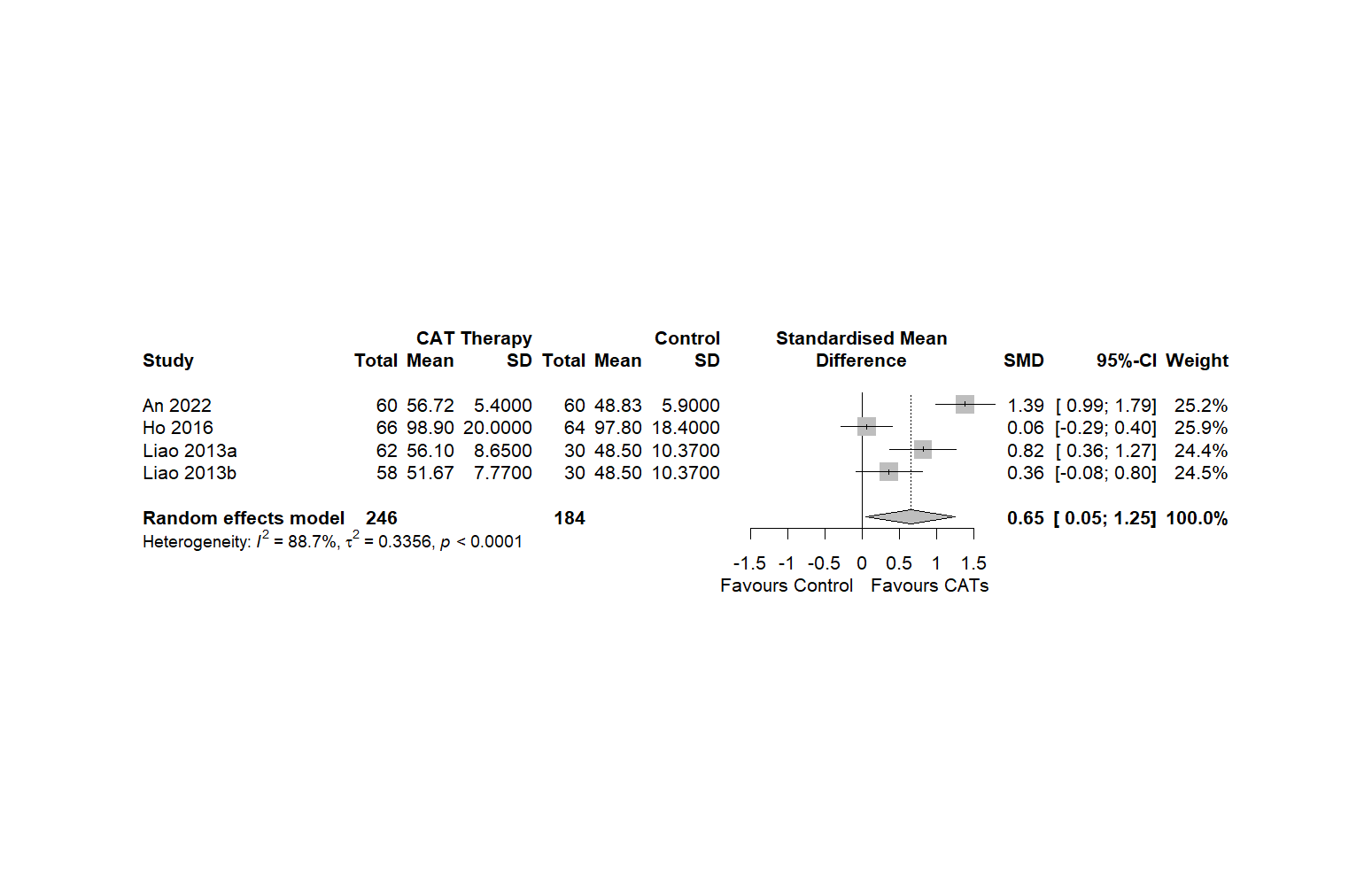
**

**Fig 12:** Forest plot for effect of CATs on quality of life between 1 to 3 weeks.


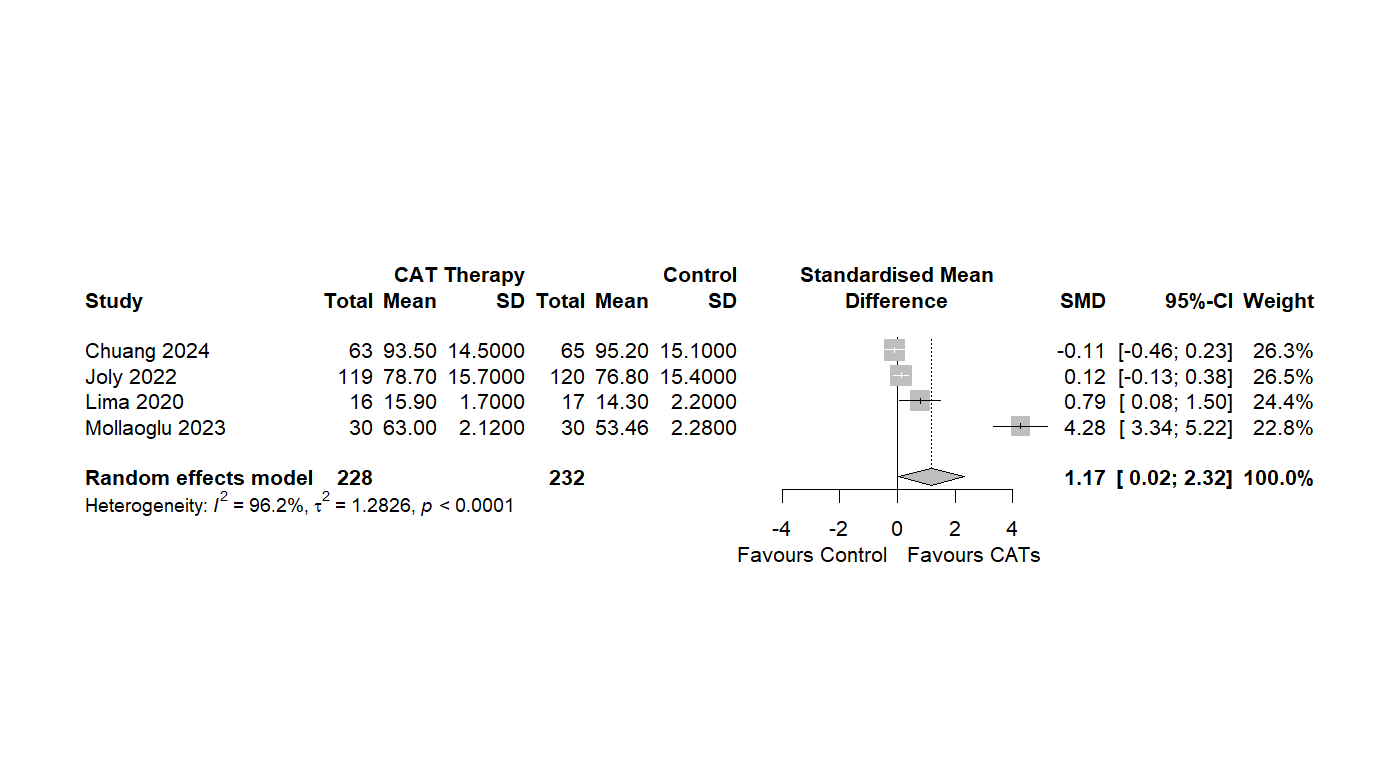


**Fig 13:** Forest plot for effect of CATs on quality of life between 4 to 6 weeks.


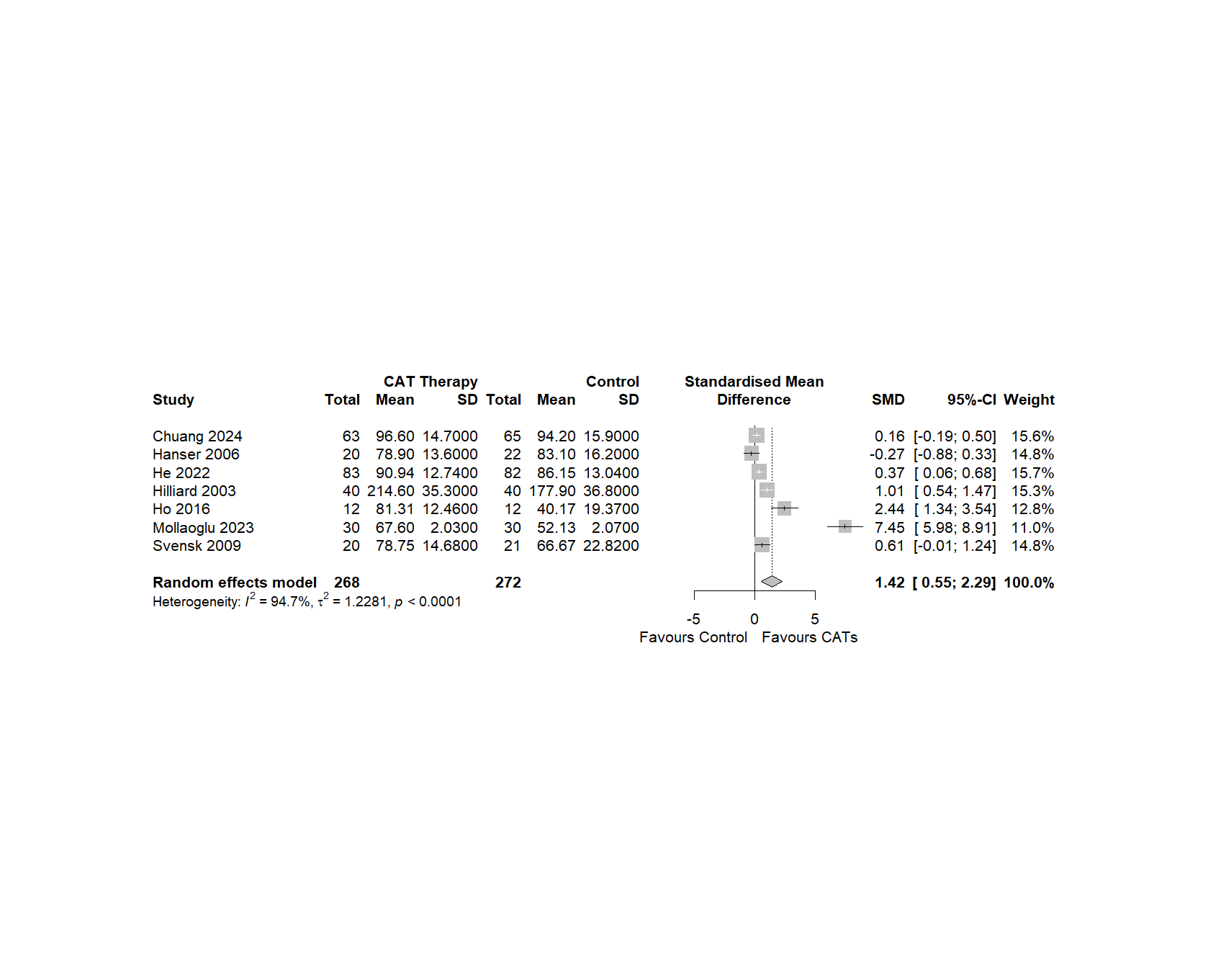


**Fig 14:** Forest plot for effect of CATs on quality of life between 2 to 3 months.


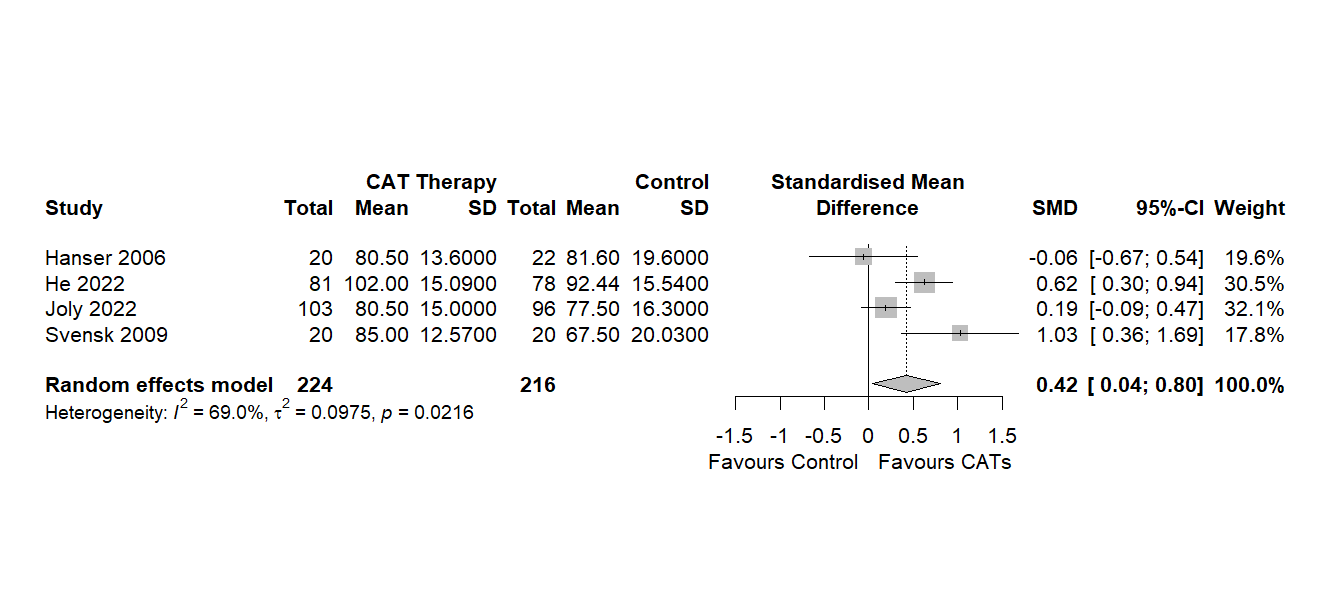


**Fig 15:** Forest plot for effect of CATs on quality of life between 4 to 6 months.

**SECONDARY ANALYSIS – ANXIETY**

*Session frequency*

**
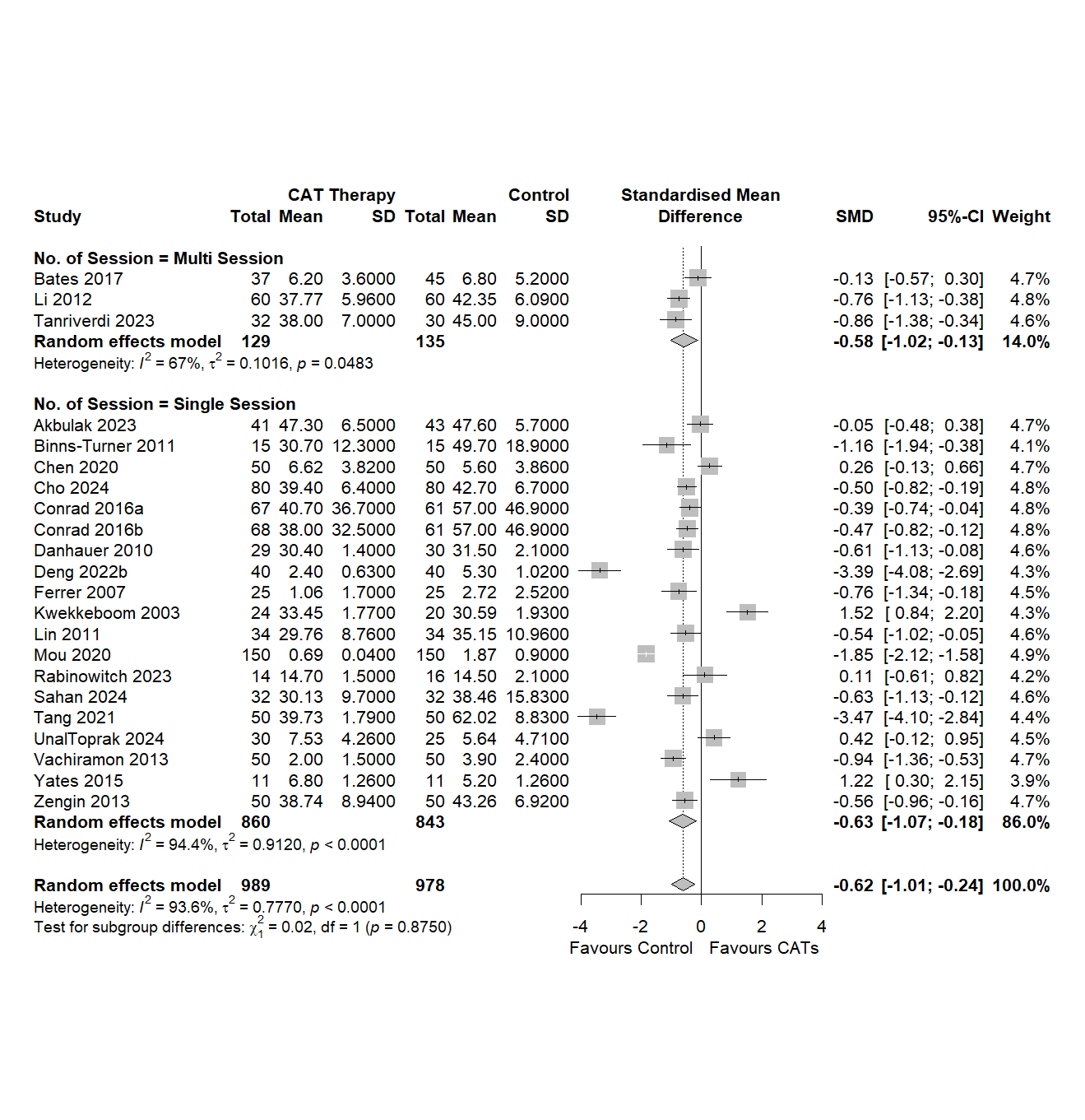
Fig 16:** Forest plot for effect of CATs on anxiety within 1 week, by session frequency.


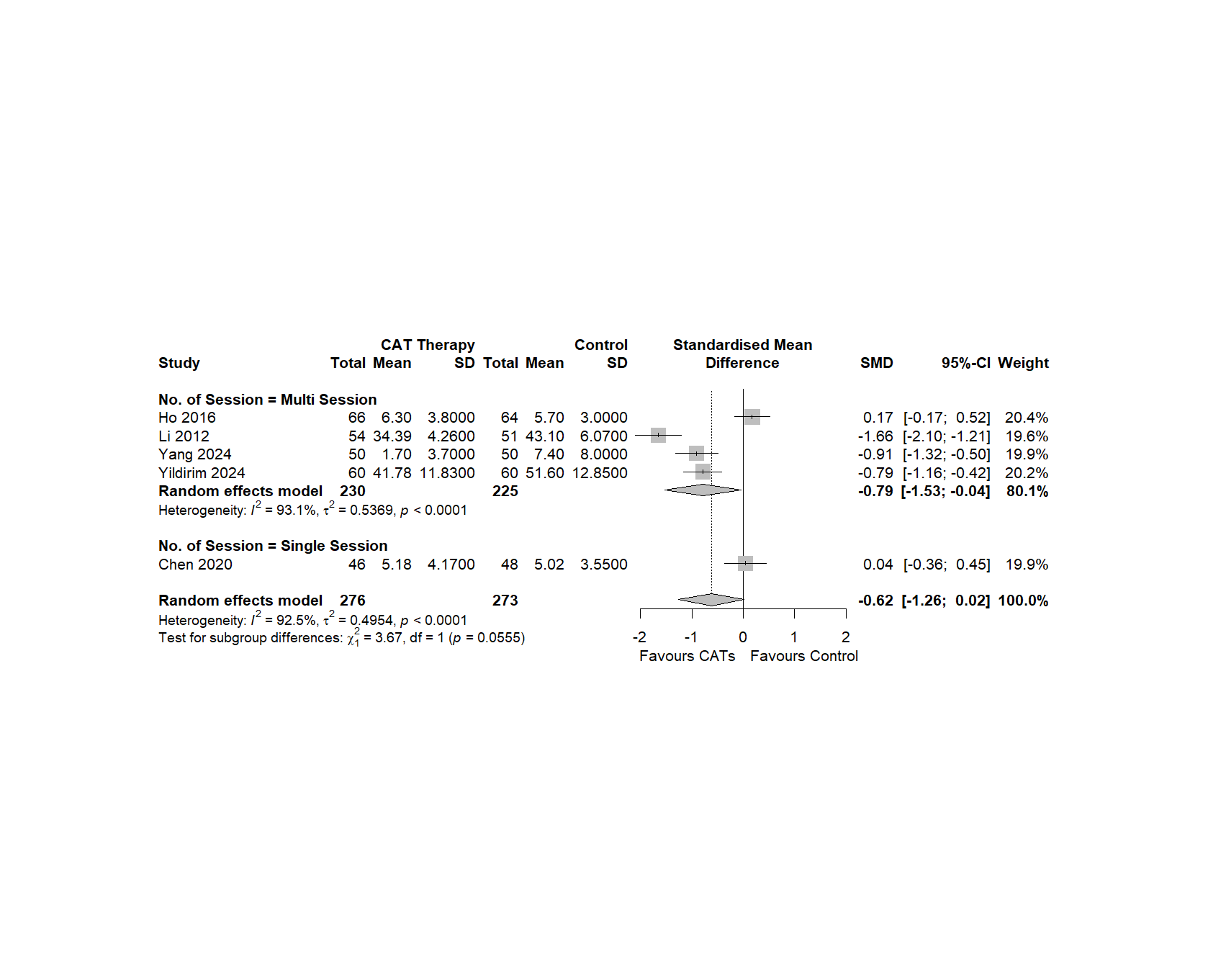
**Fig 17:** Forest plot for effect of CATs on anxiety between 1 to 3 weeks, by session frequency.

*Intervention type*


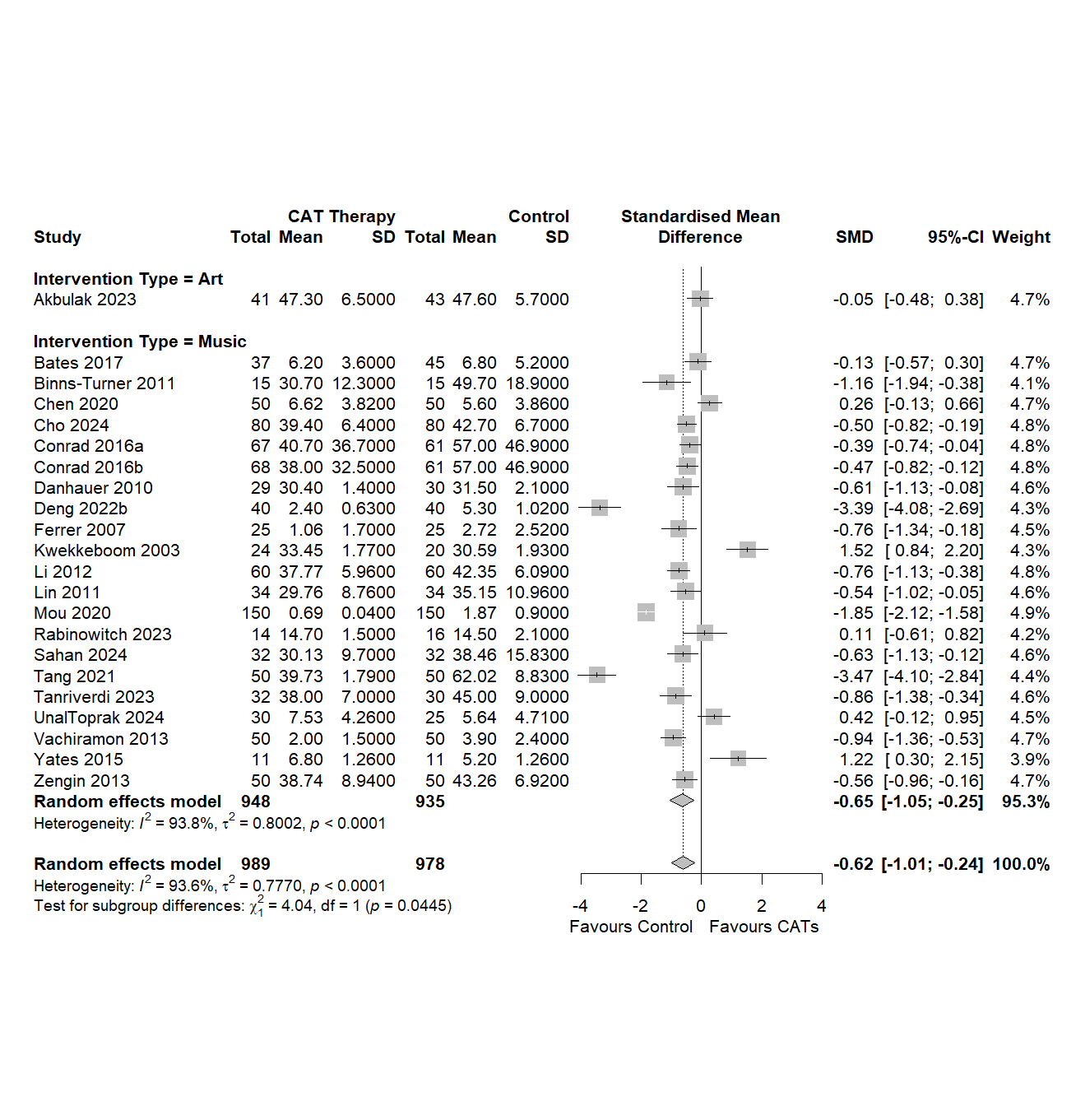
**Fig 18:** Forest plot for effect of CATs on anxiety within 1 week, by intervention type.


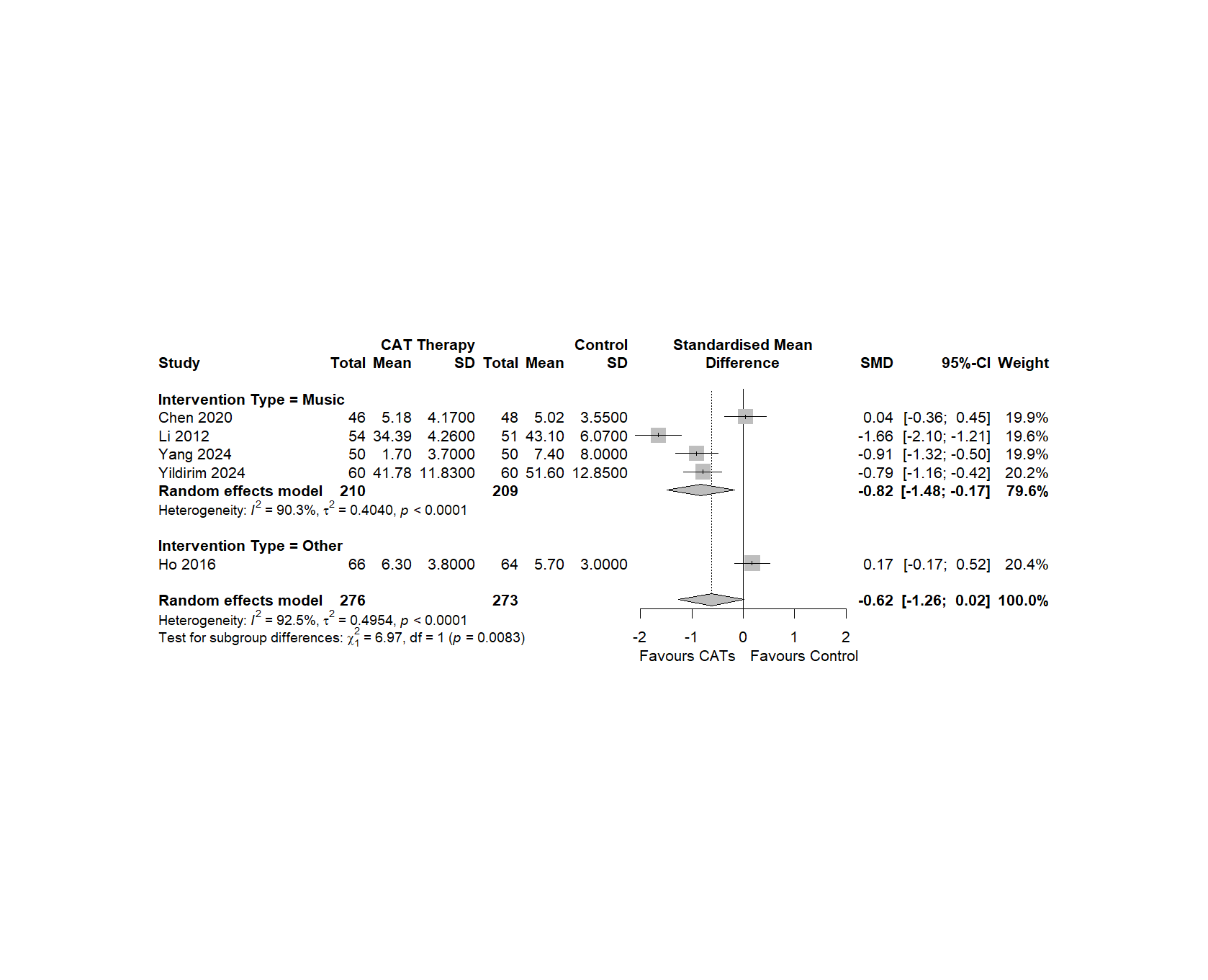
**Fig 19:** Forest plot for effect of CATs on anxiety between 1 to 3 weeks, by intervention type.


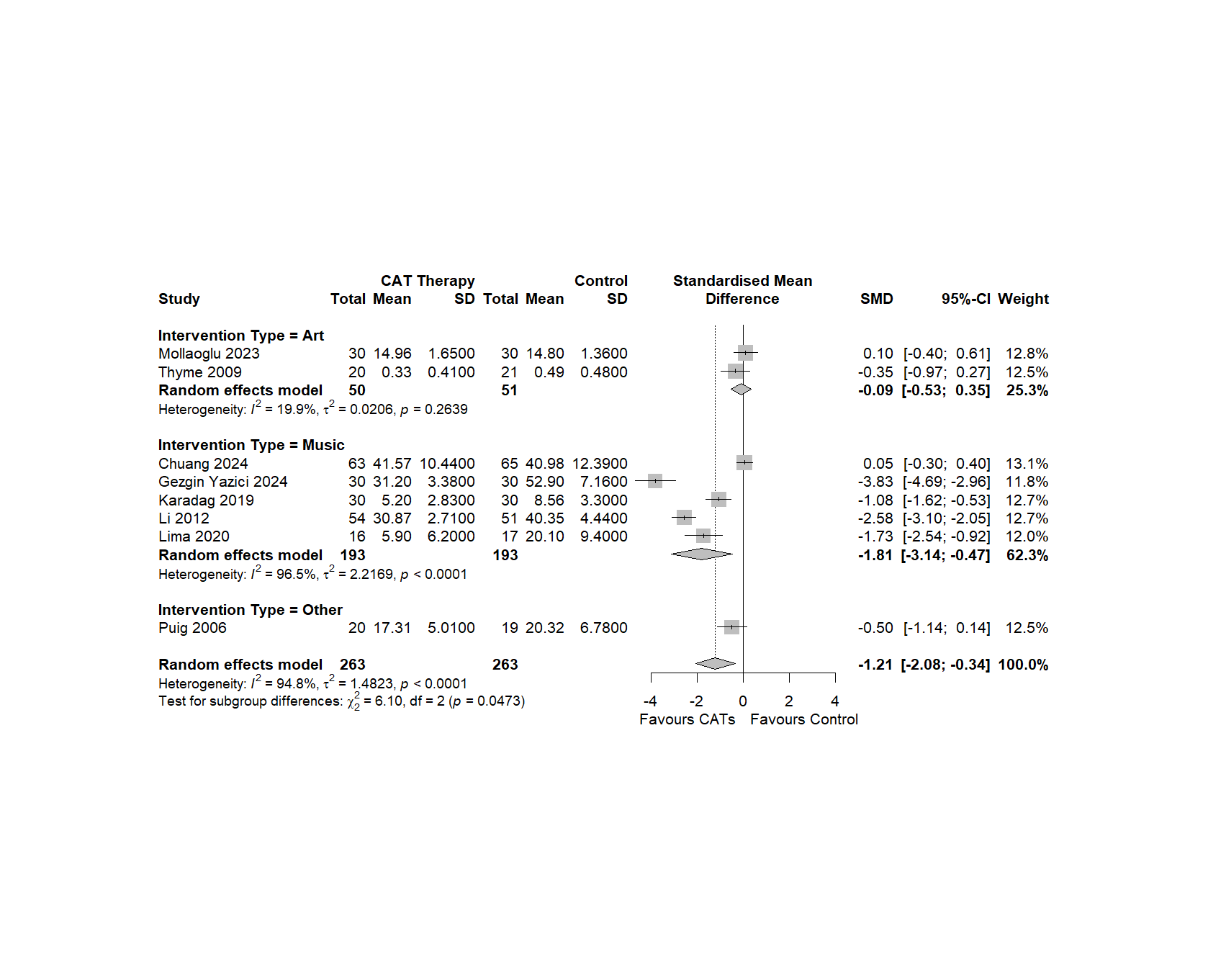


**Fig 20:** Forest plot for effect of CATs on anxiety between 4 to 6 weeks, by intervention type.


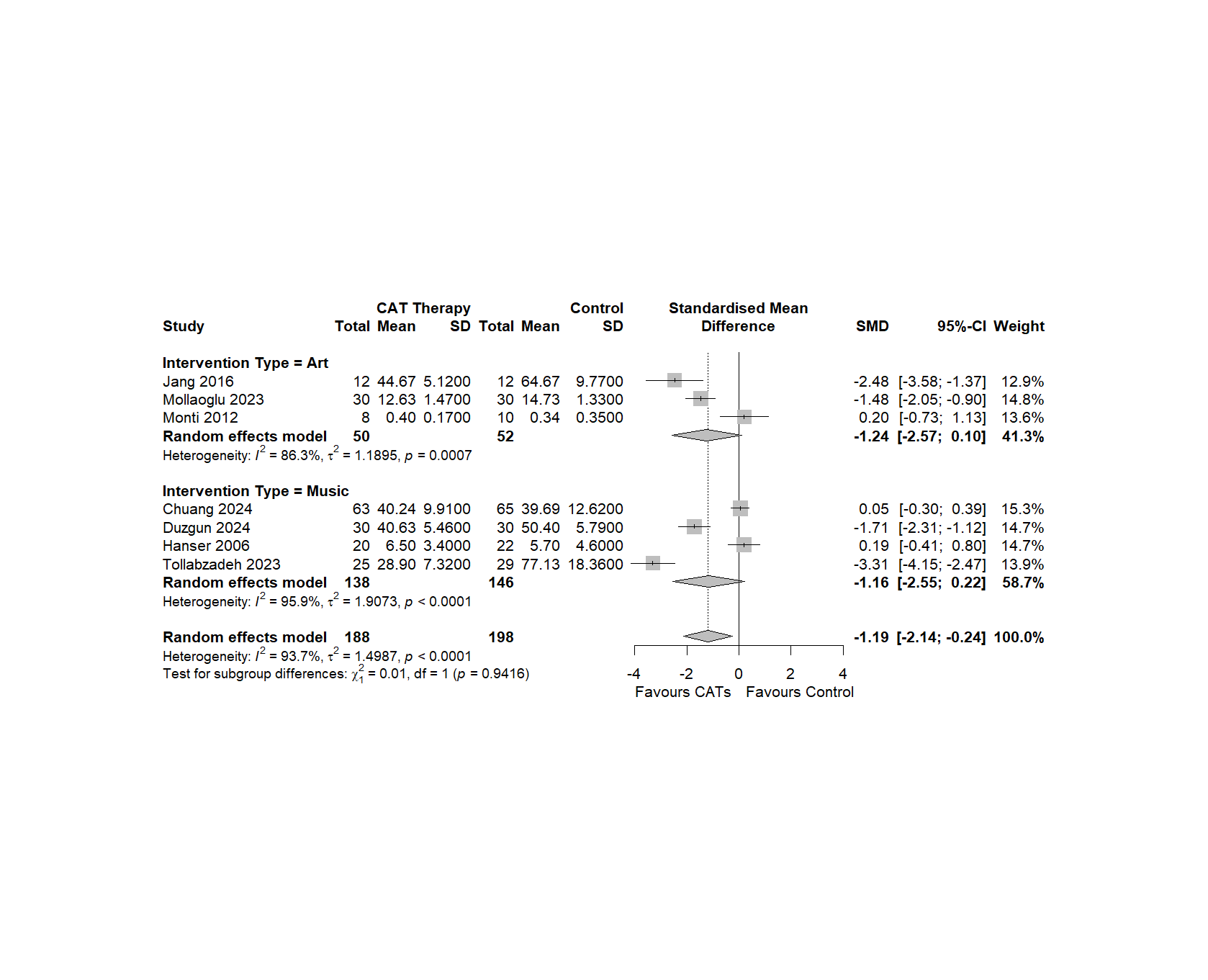


**Fig 21:** Forest plot for effect of CATs on anxiety between 2 to 3 months, by intervention type.


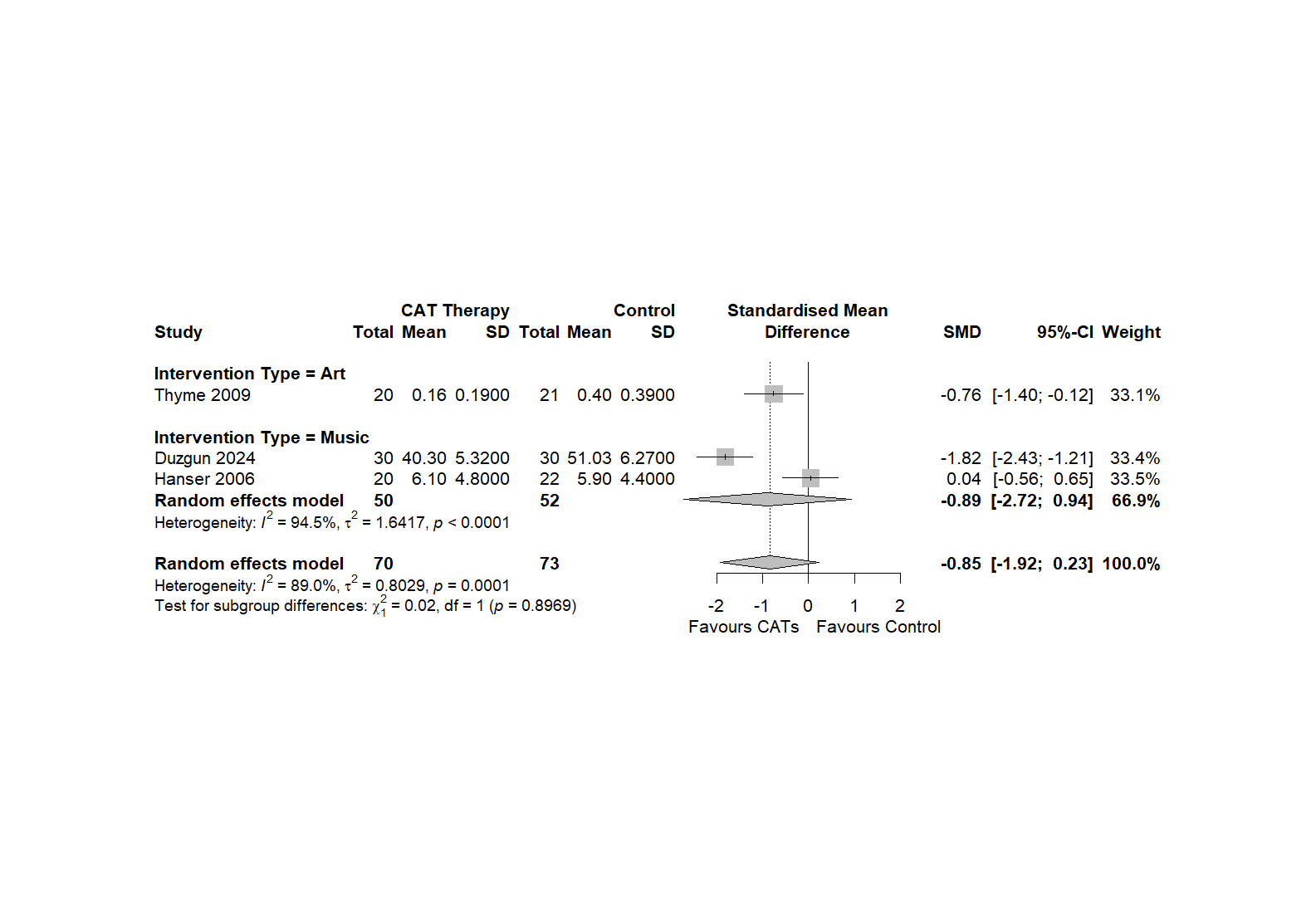


**Fig 22:** Forest plot for effect of CATs on anxiety between 4 to 6 months, by intervention type.

*Treatment setting*


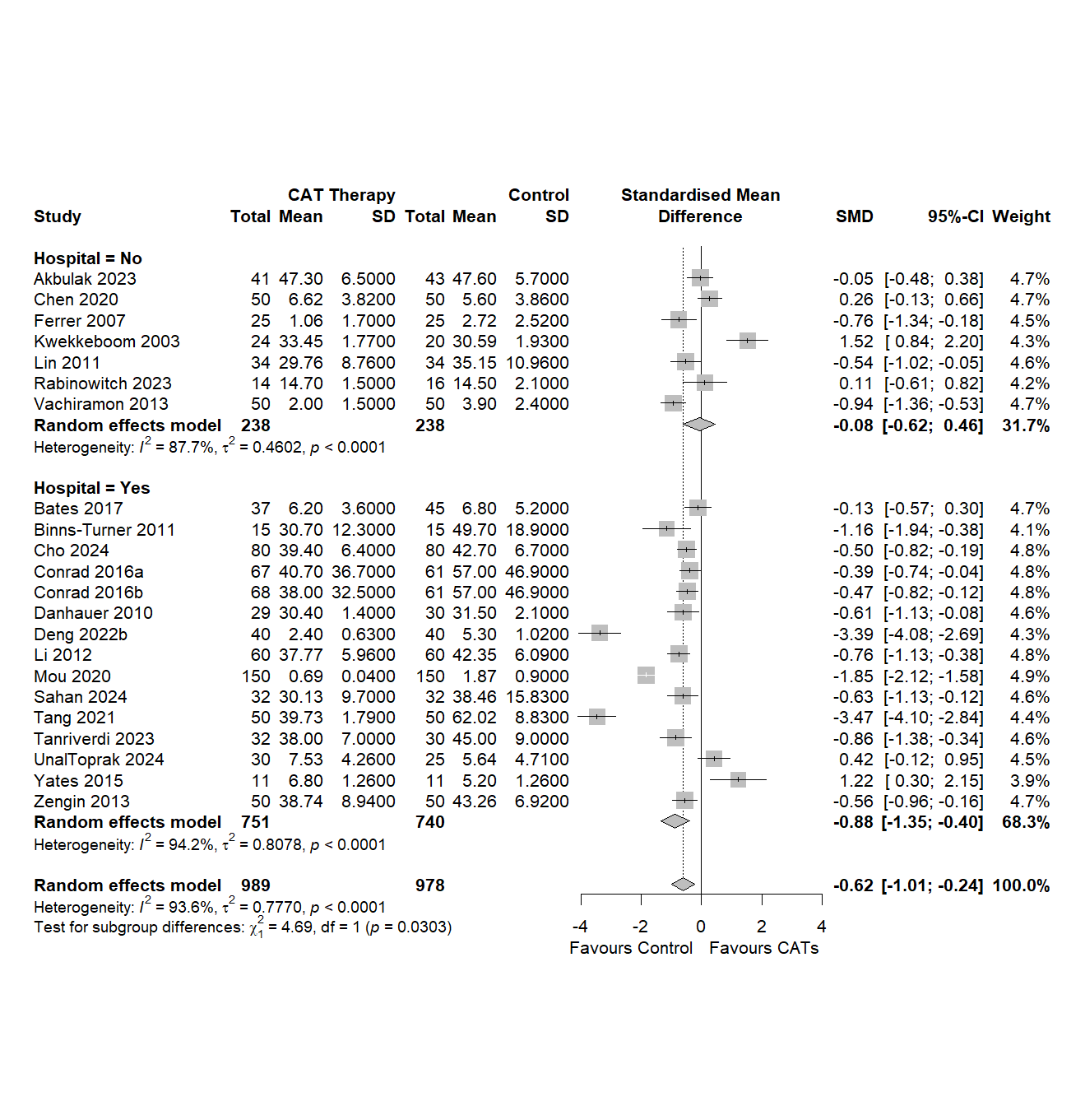
**Fig 23:** Forest plot for effect of CATs on anxiety within 1 week, by treatment setting.


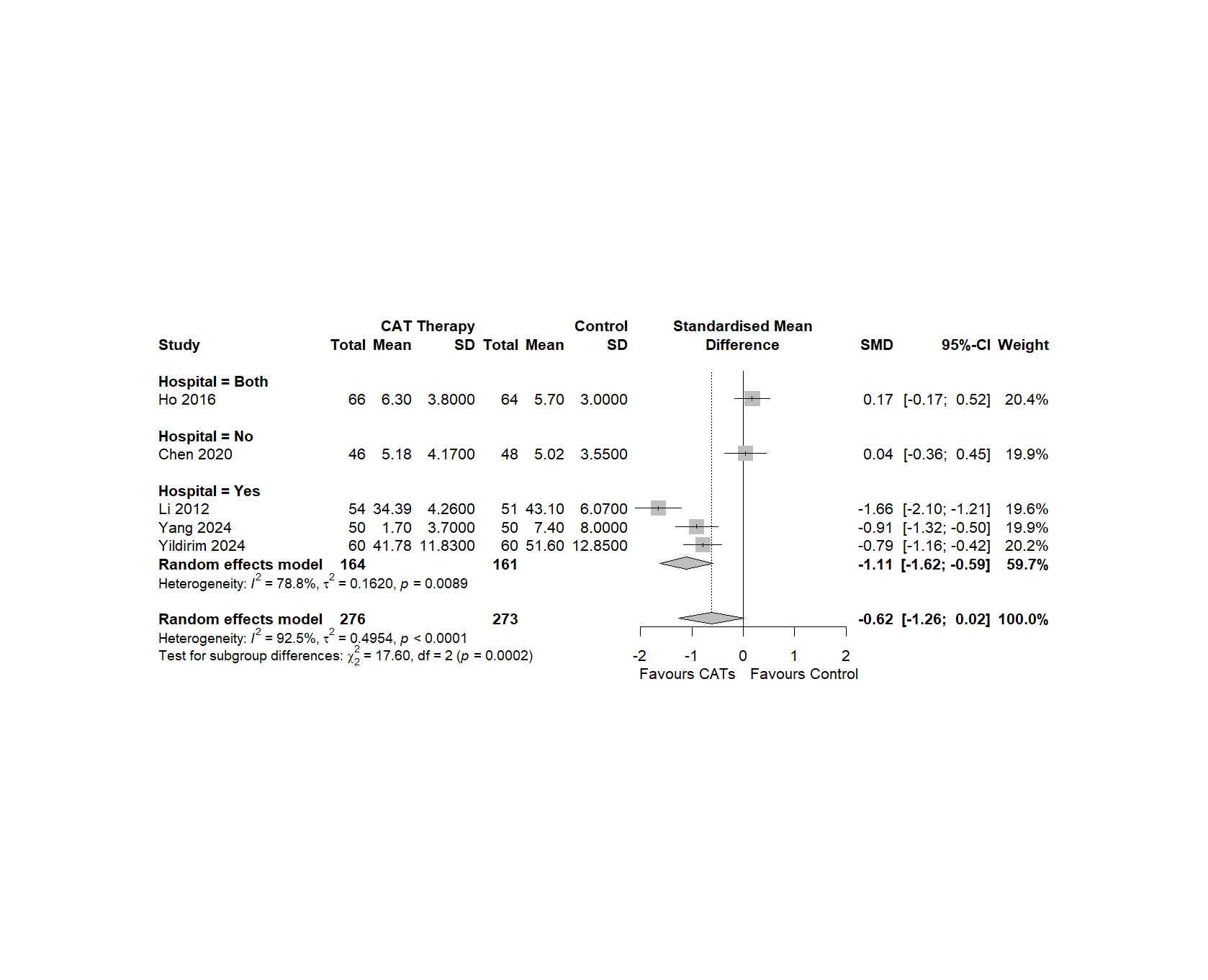
**Fig 24:** Forest plot for effect of CATs on anxiety between 1 to 3 weeks, by treatment setting.


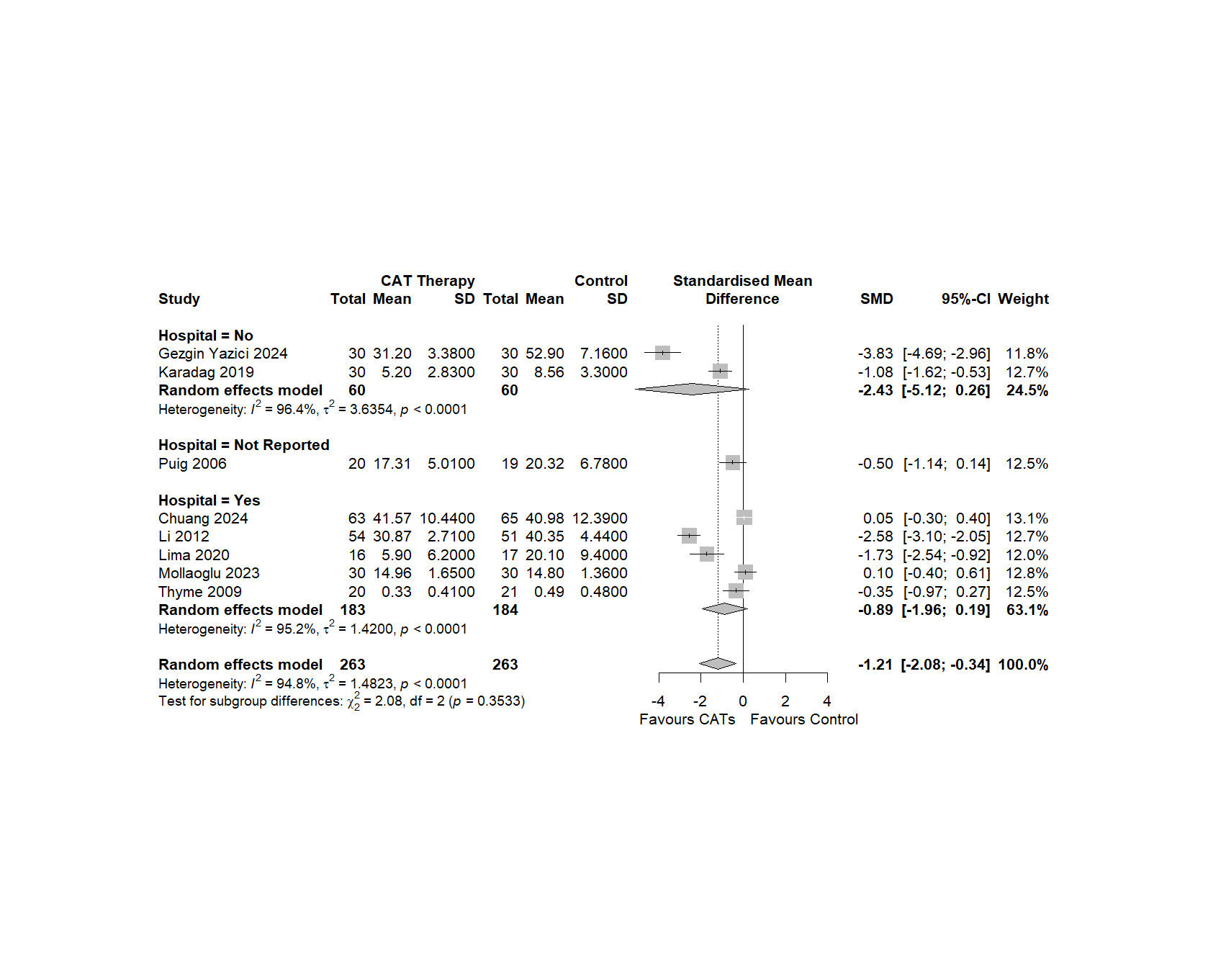
**Fig 25:** Forest plot for effect of CATs on anxiety between 4 to 6 weeks, by treatment setting.


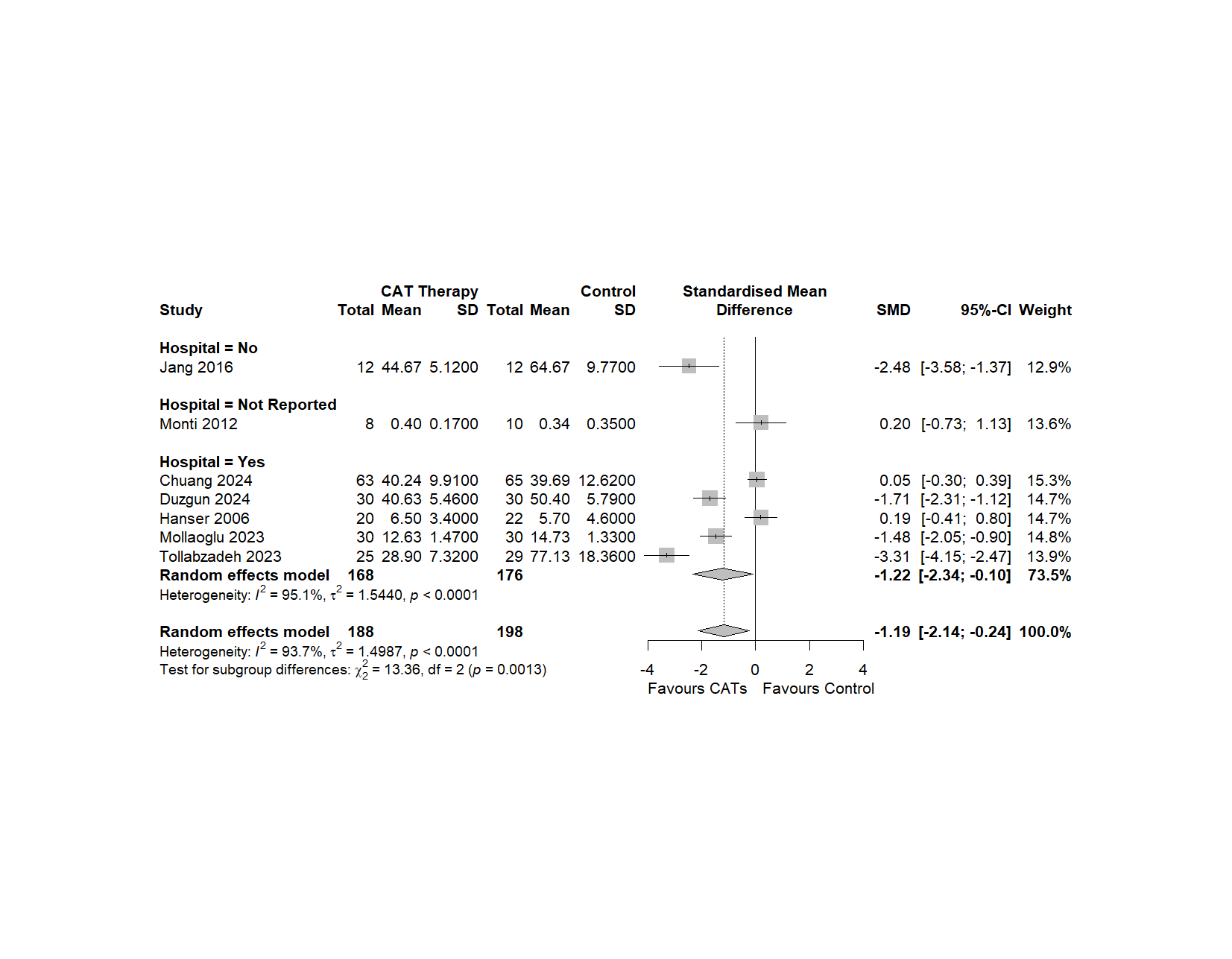
**Fig 26:** Forest plot for effect of CATs on anxiety between 2 to 3 months, by treatment setting.

*Region*

**
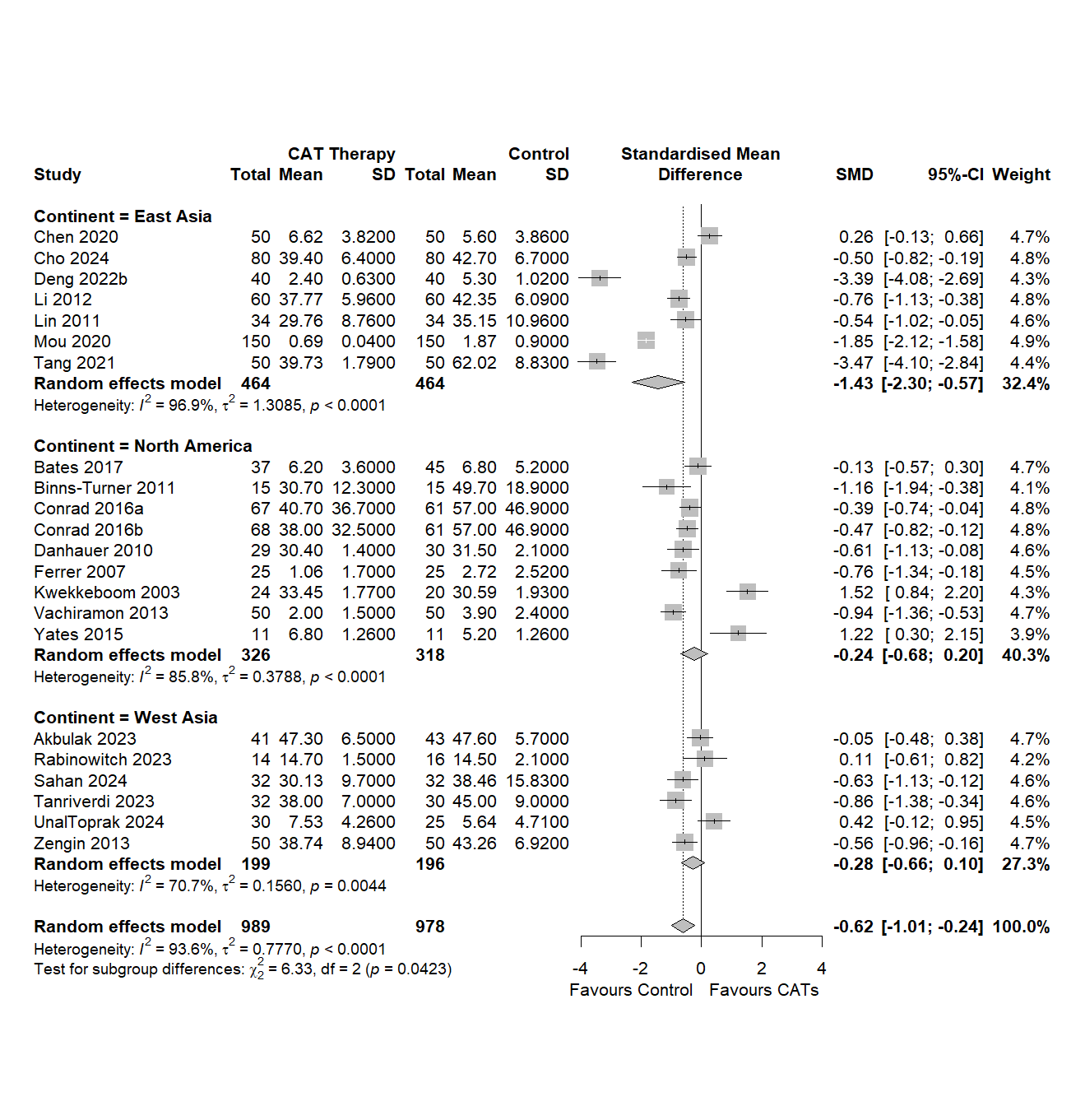
Fig 27:** Forest plot for effect of CATs on anxiety within 1 week, by region.


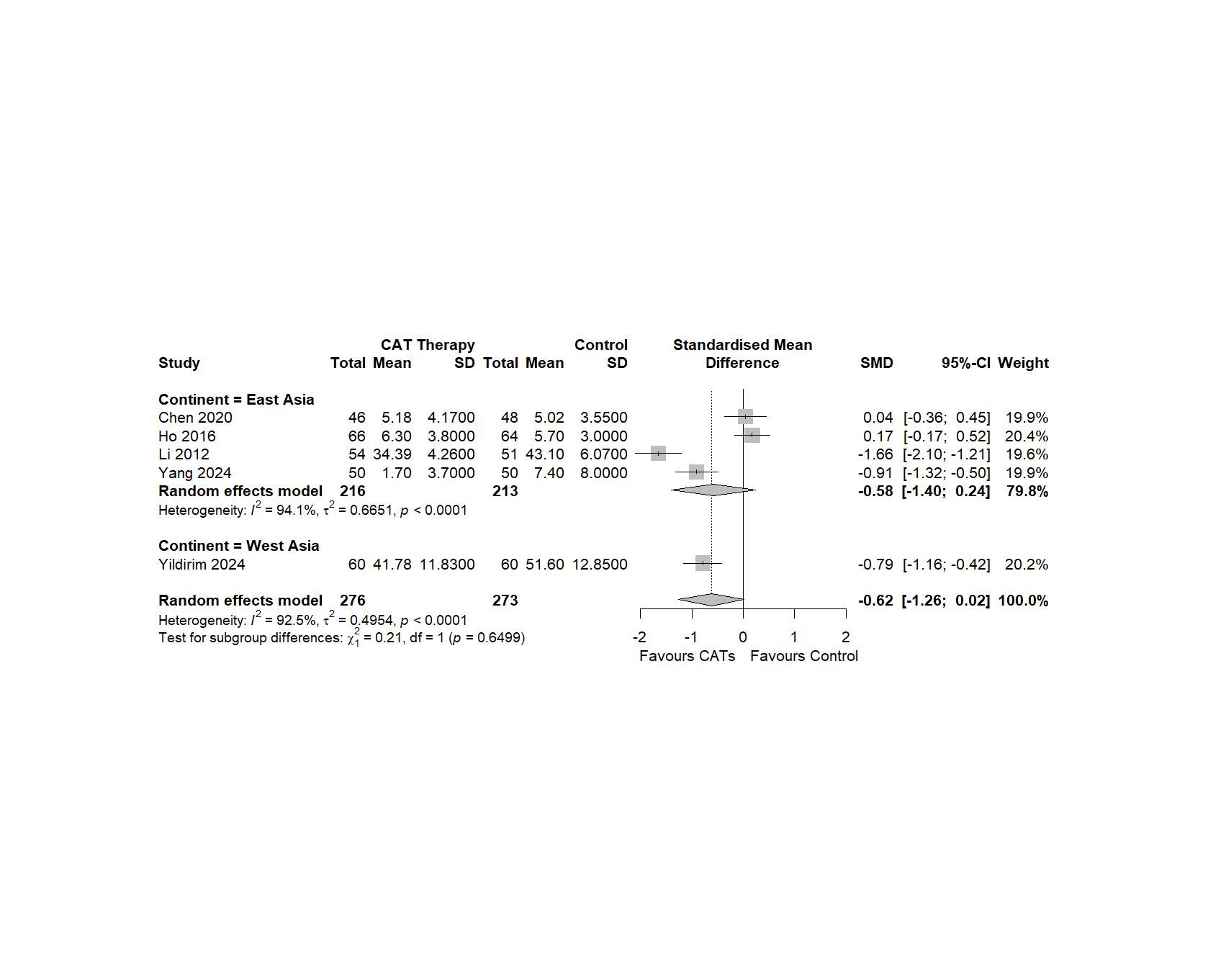


**Fig 28:** Forest plot for effect of CATs on anxiety between 1 to 3 weeks, by region.


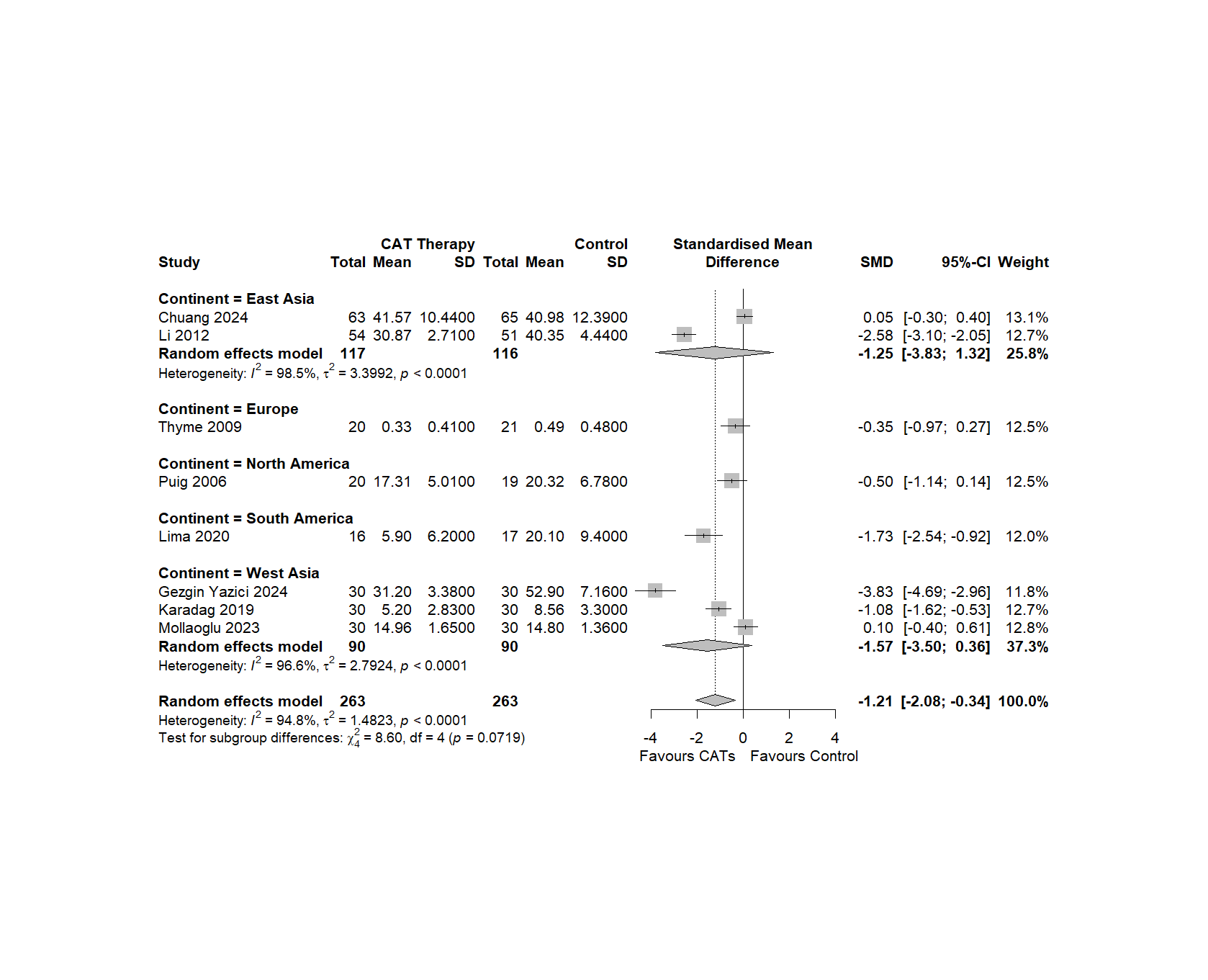
**Fig 29:** Forest plot for effect of CATs on anxiety between 4 to 6 weeks, by region.


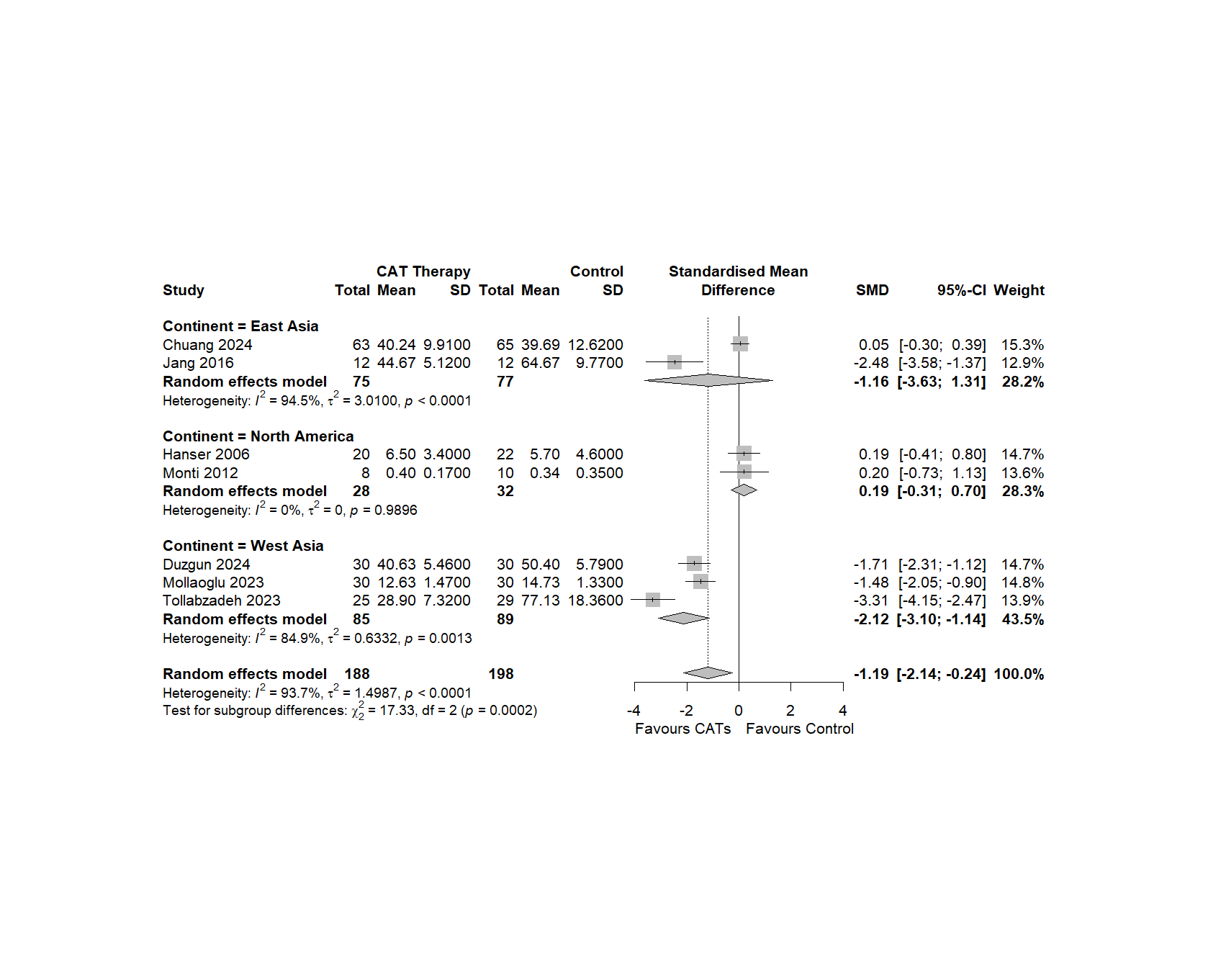
**Fig 30:** Forest plot for effect of CATs on anxiety between 2 to 3 months, by region.


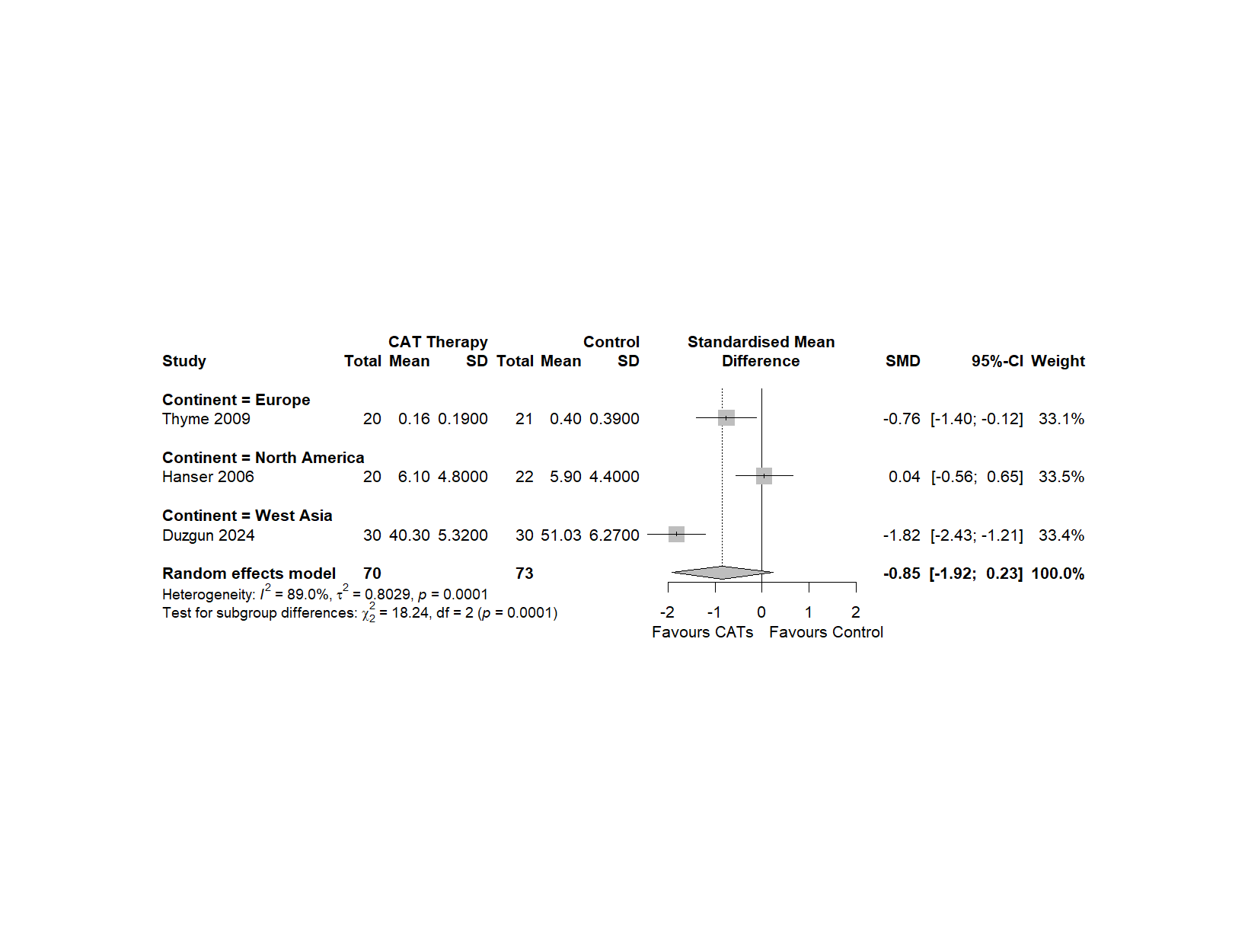
**Fig 31:** Forest plot for effect of CATs on anxiety between 4 to 6 months, by region.

**SECONDARY ANALYSIS – DEPRESSION**

*Session frequency*

**
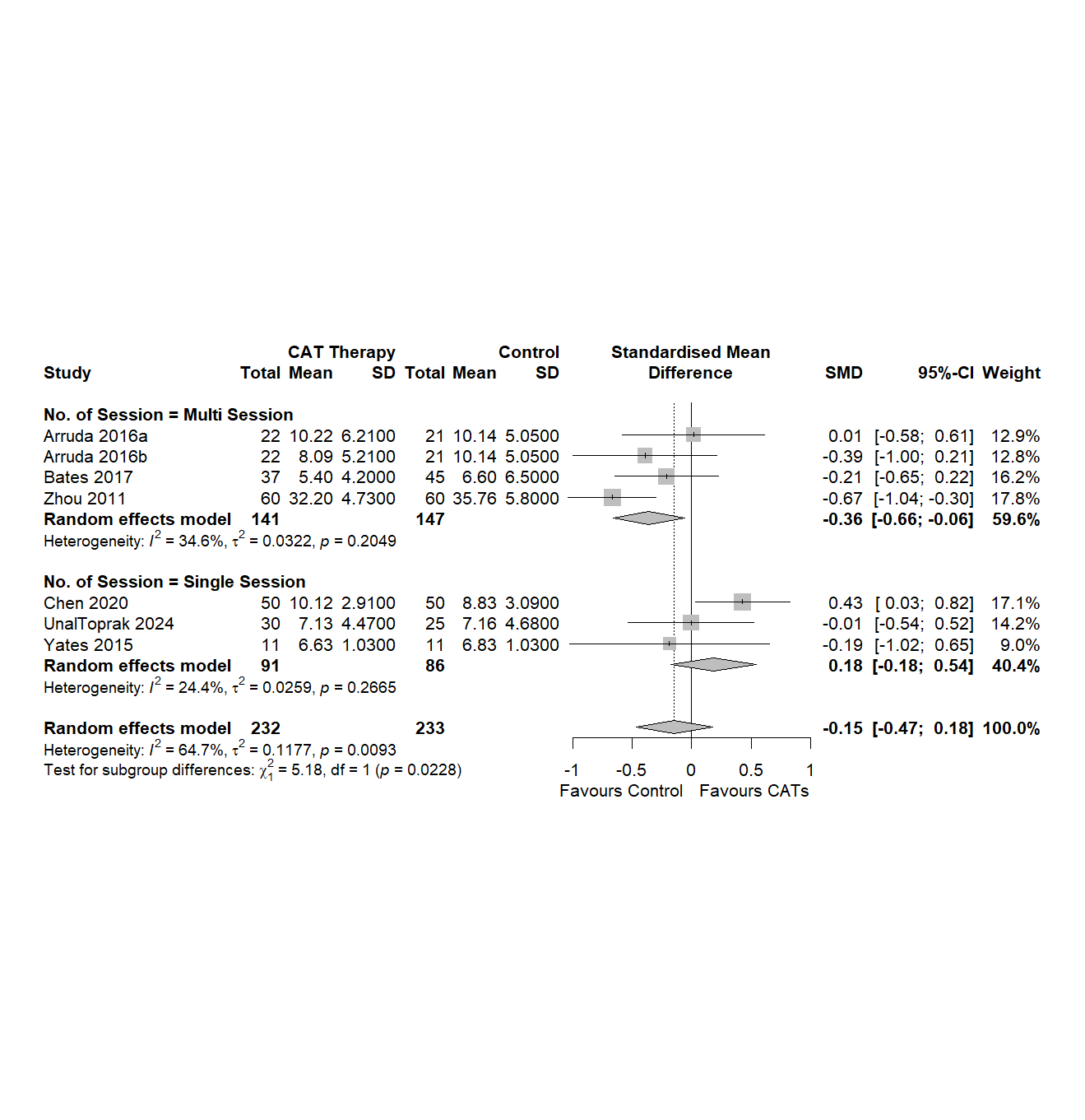
Fig 32:** Forest plot for effect of CATs on depression within 1 week, by session frequency.


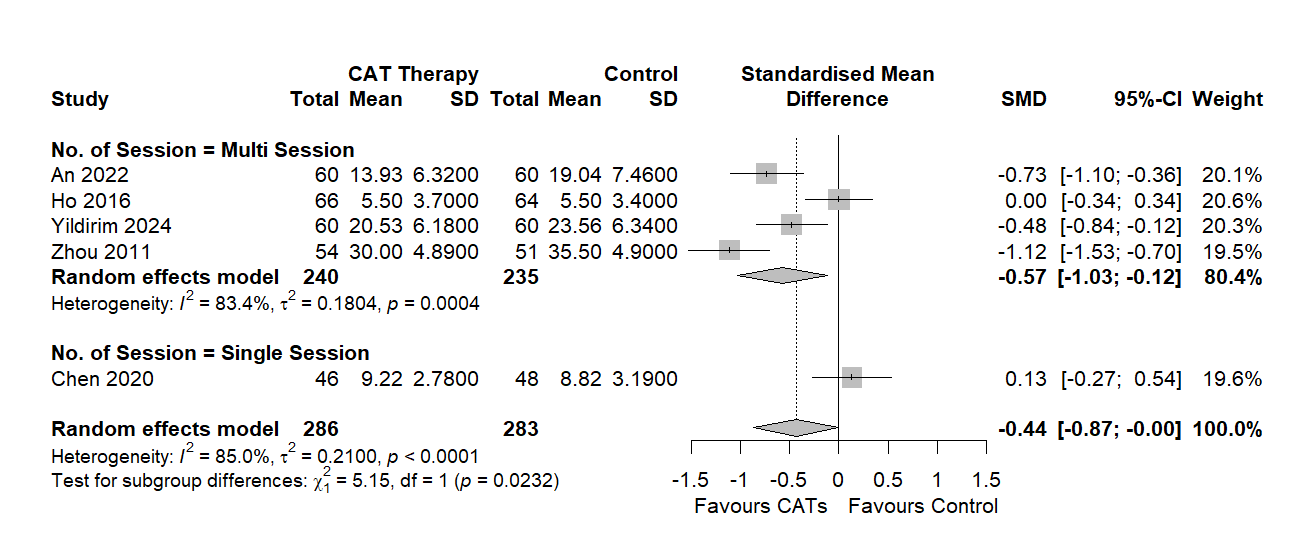
**Fig 33:** Forest plot for effect of CATs on depression between 1 to 3 weeks, by session frequency.

*Intervention type*

*
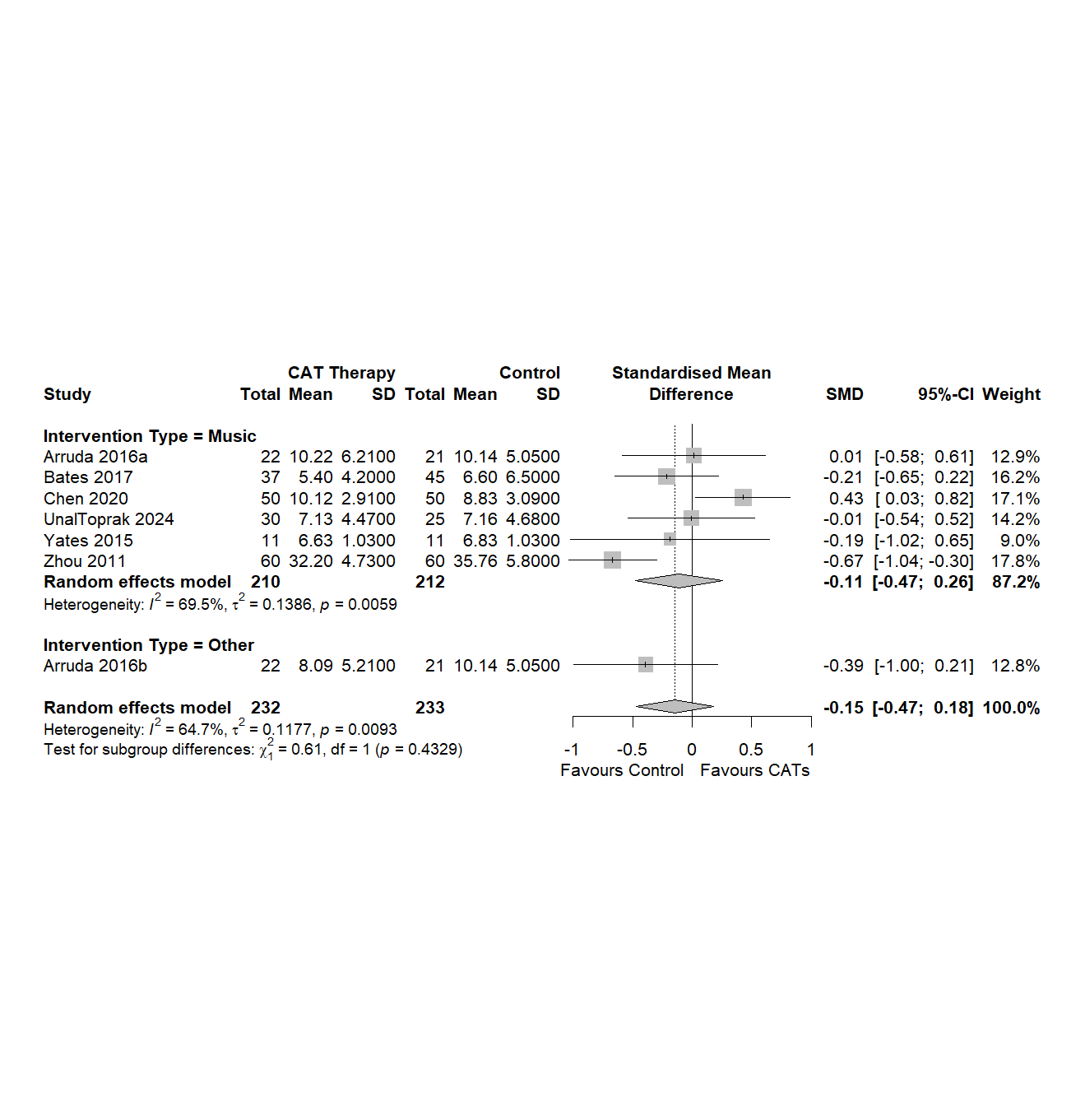
***Fig 34:** Forest plot for effect of CATs on depression within 1 week, by intervention type.


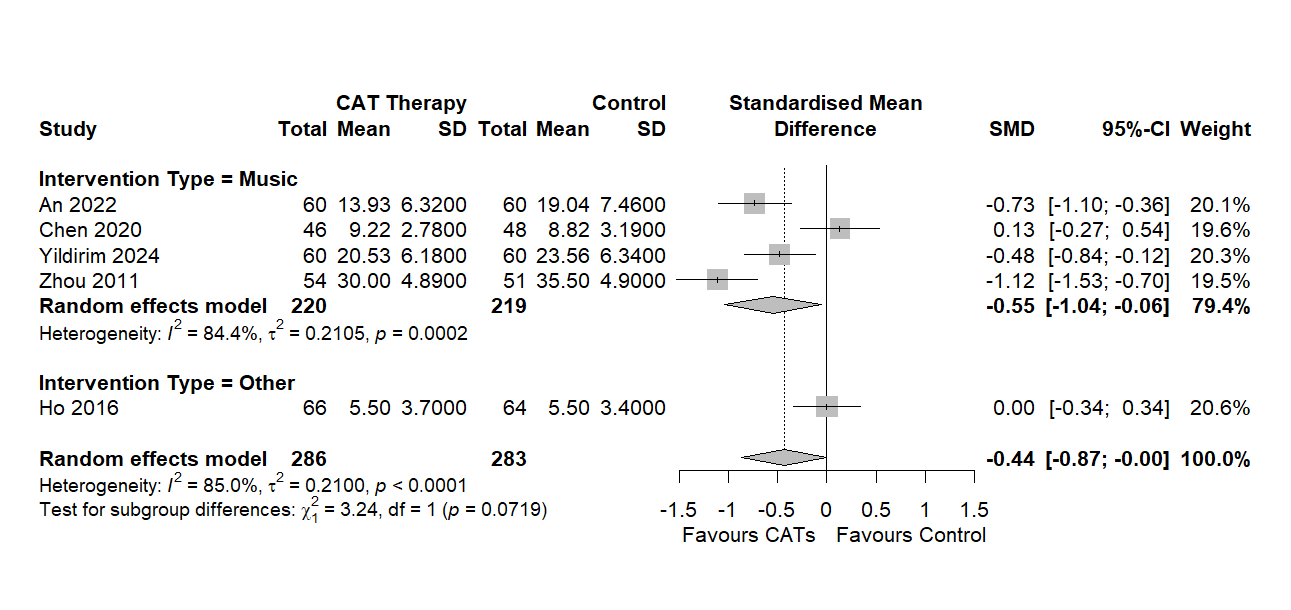
**Fig 35:** Forest plot for effect of CATs on depression between 1 to 3 weeks, by intervention type.


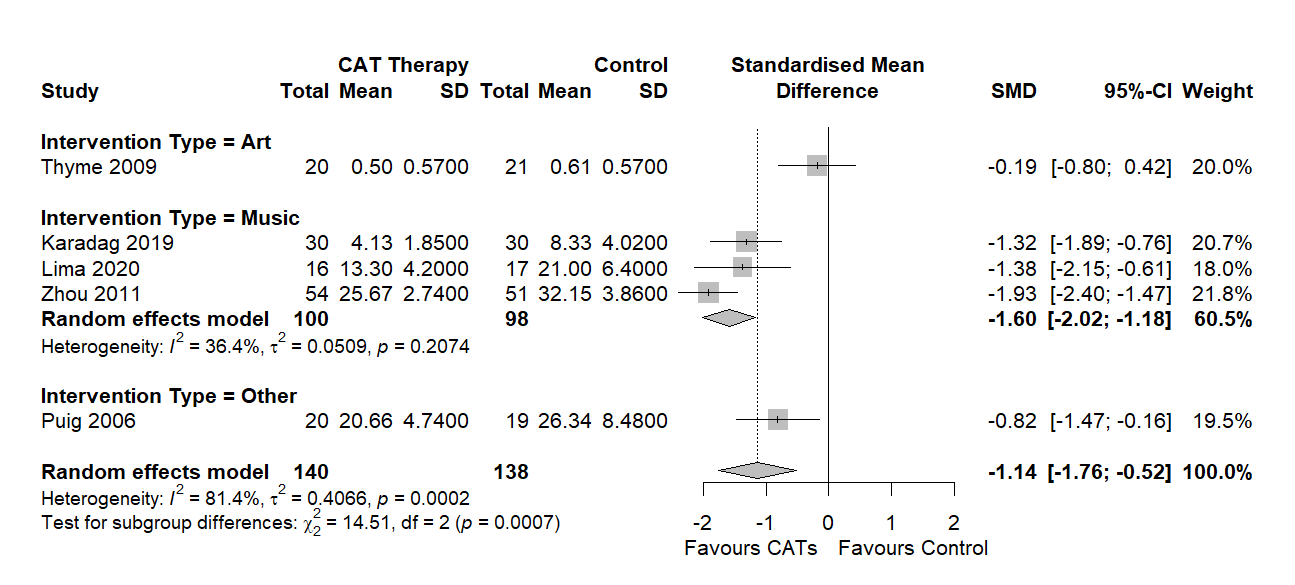
**Fig 36:** Forest plot for effect of CATs on depression between 4 to 6 weeks, by intervention type.

*Treatment setting*

*
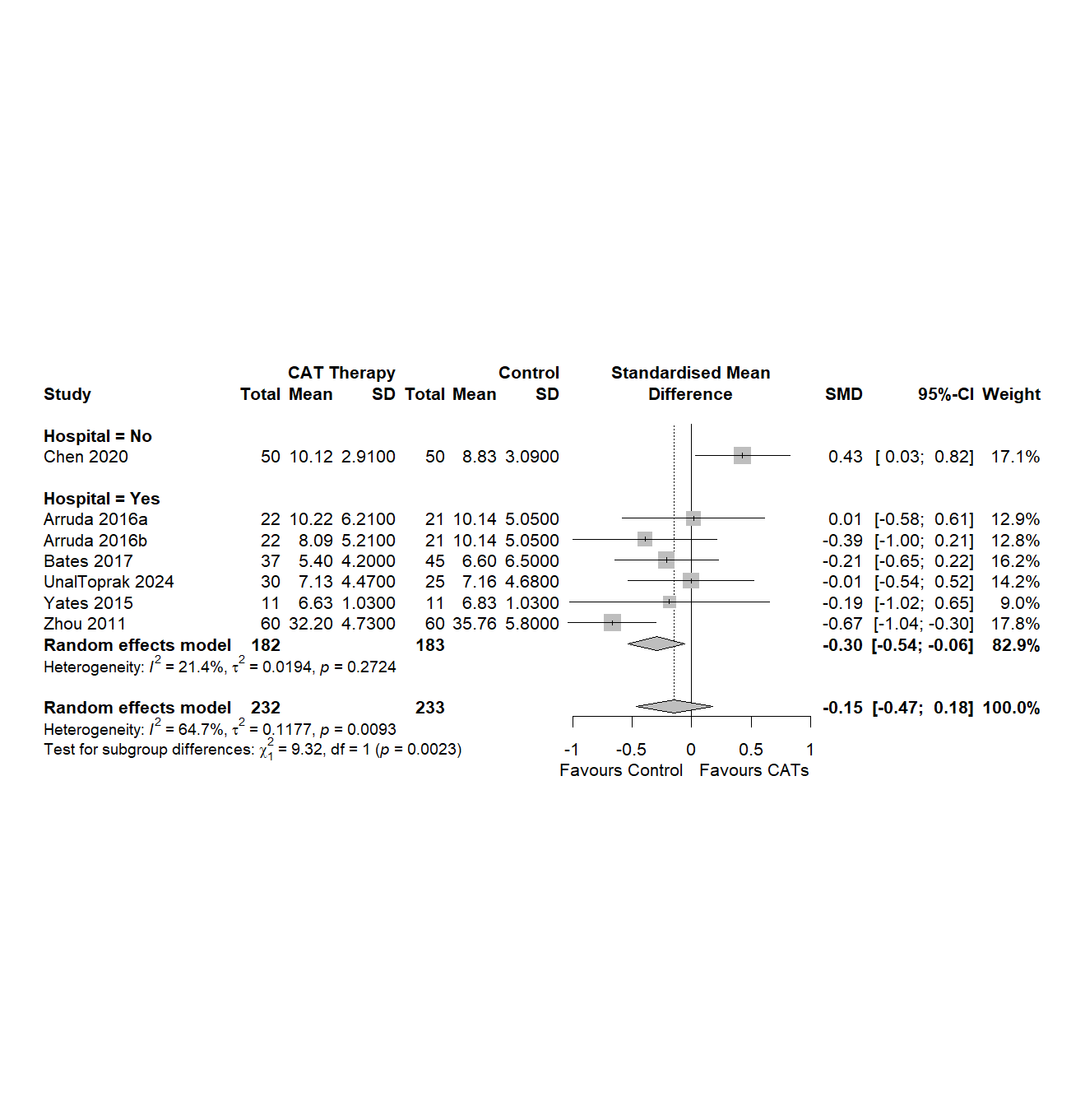
***Fig 37:** Forest plot for effect of CATs on depression within 1 week, by treatment setting.


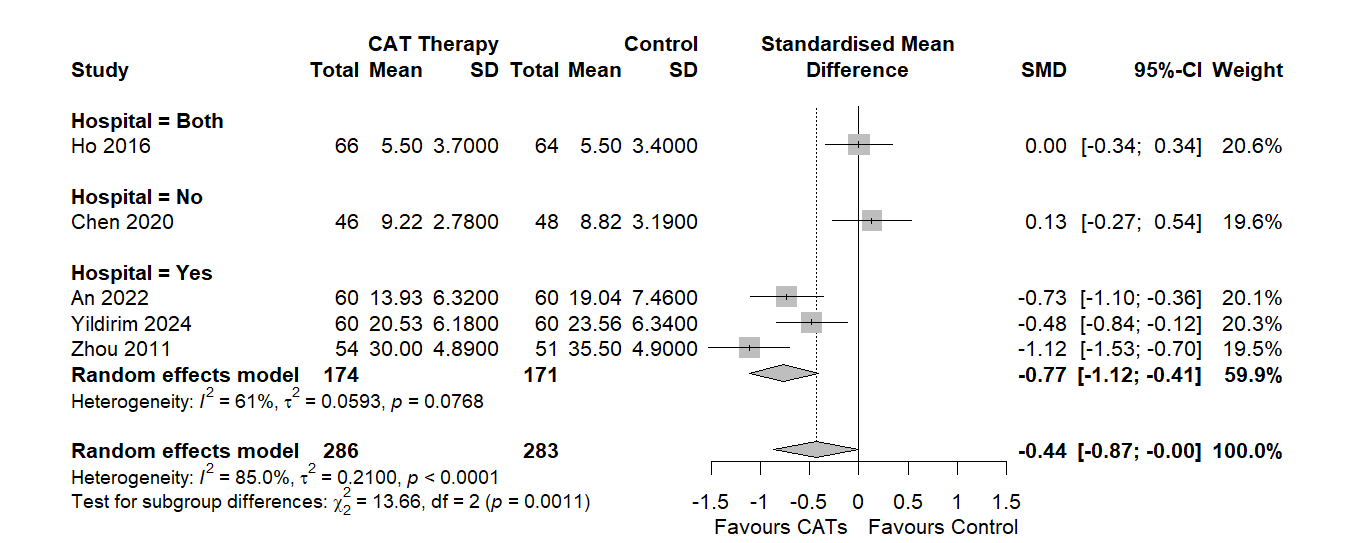
**Fig 38:** Forest plot for effect of CATs on depression between 1 to 3 weeks, by treatment setting.


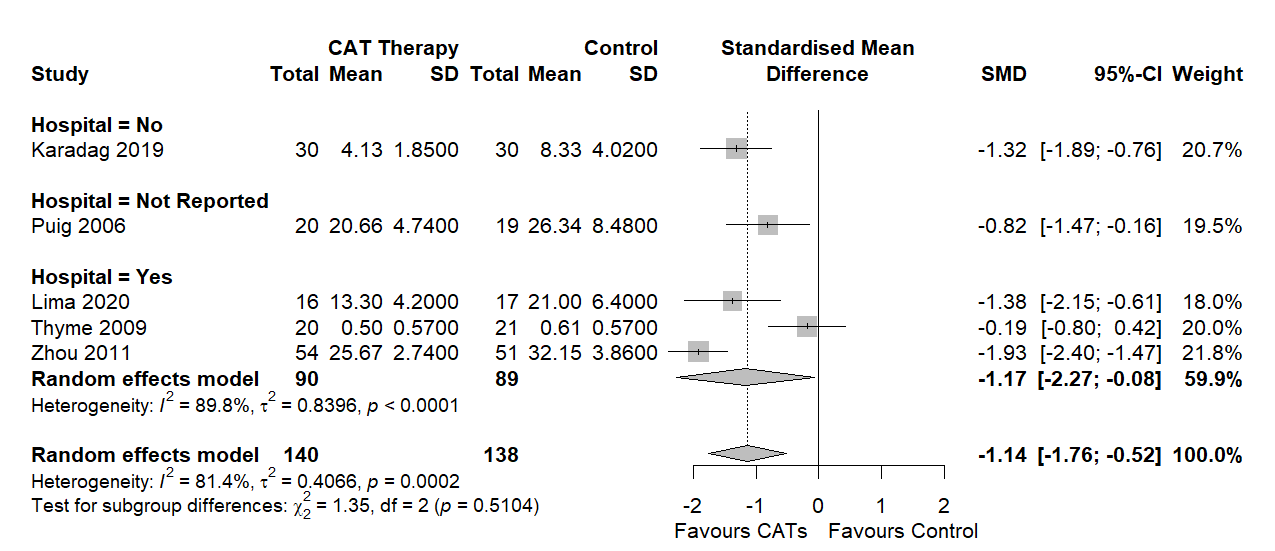
**Fig 39:** Forest plot for effect of CATs on depression between 4 to 6 weeks, by treatment setting.

*Region*

*
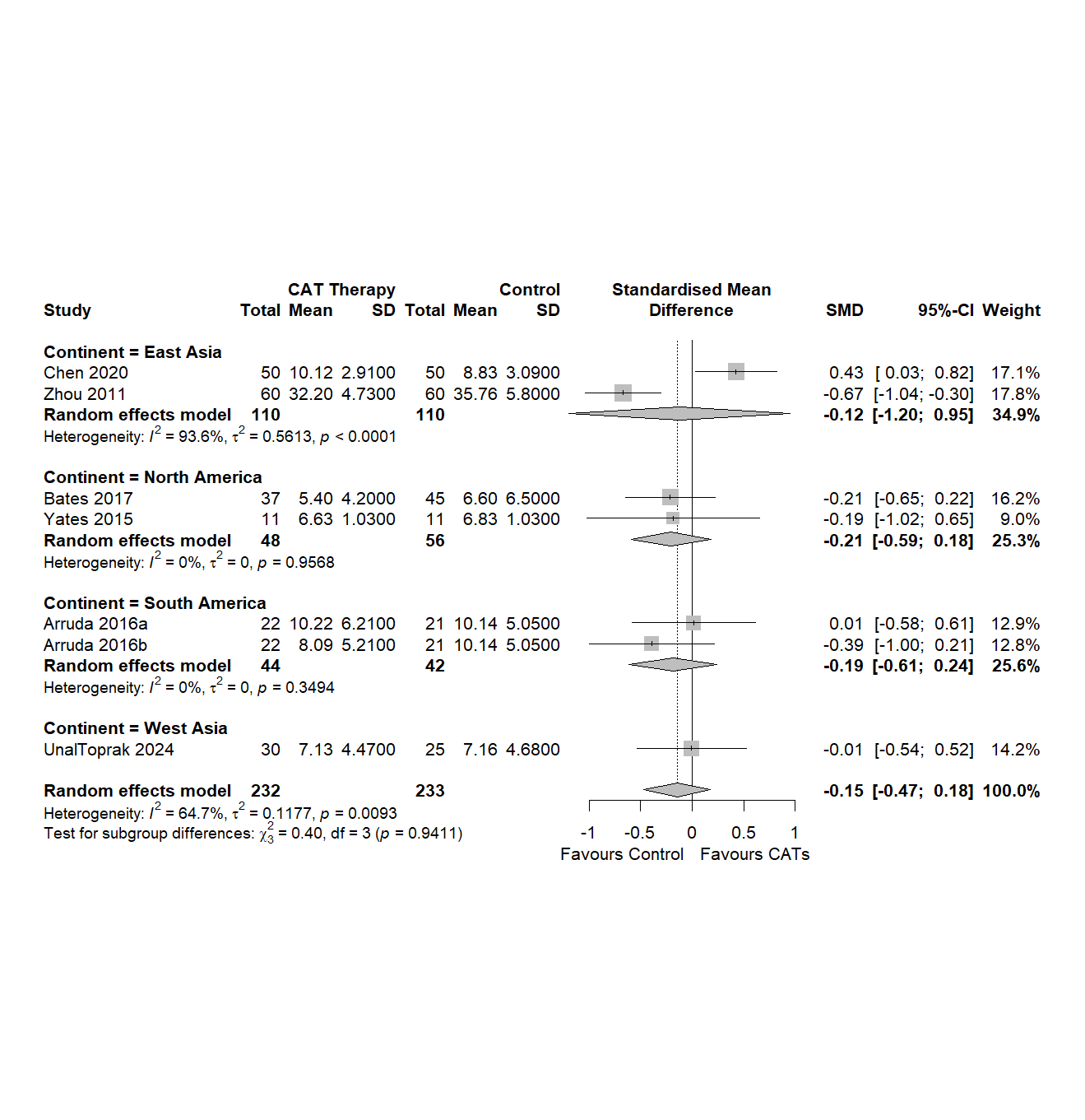
***Fig 40:** Forest plot for effect of CATs on depression within 1 week, by region.


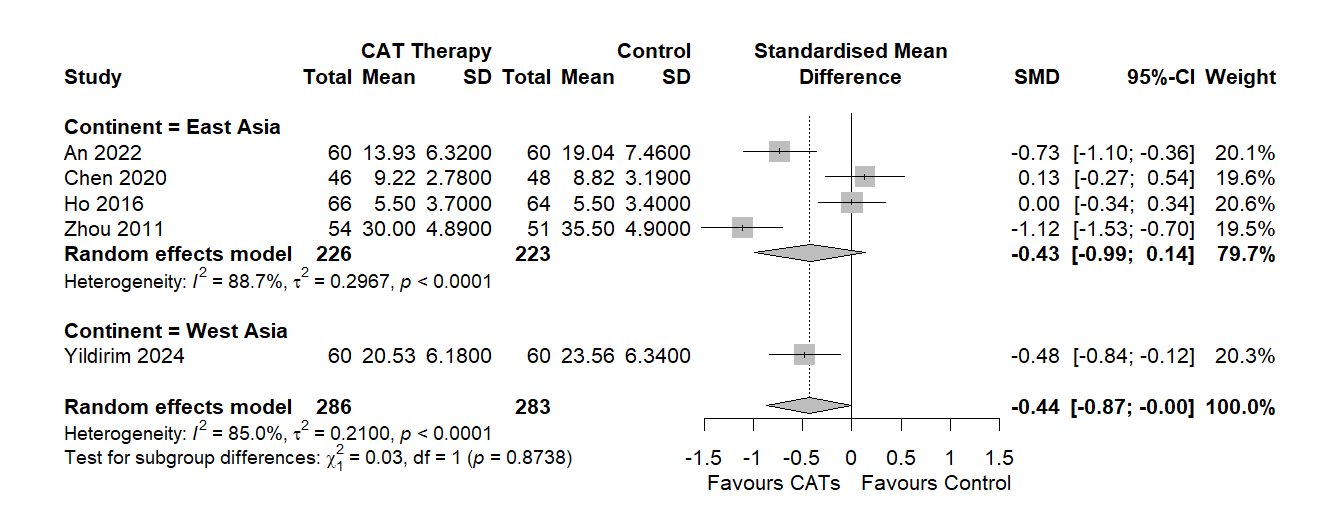
**Fig 41:** Forest plot for effect of CATs on depression between 1 to 3 weeks, by region.


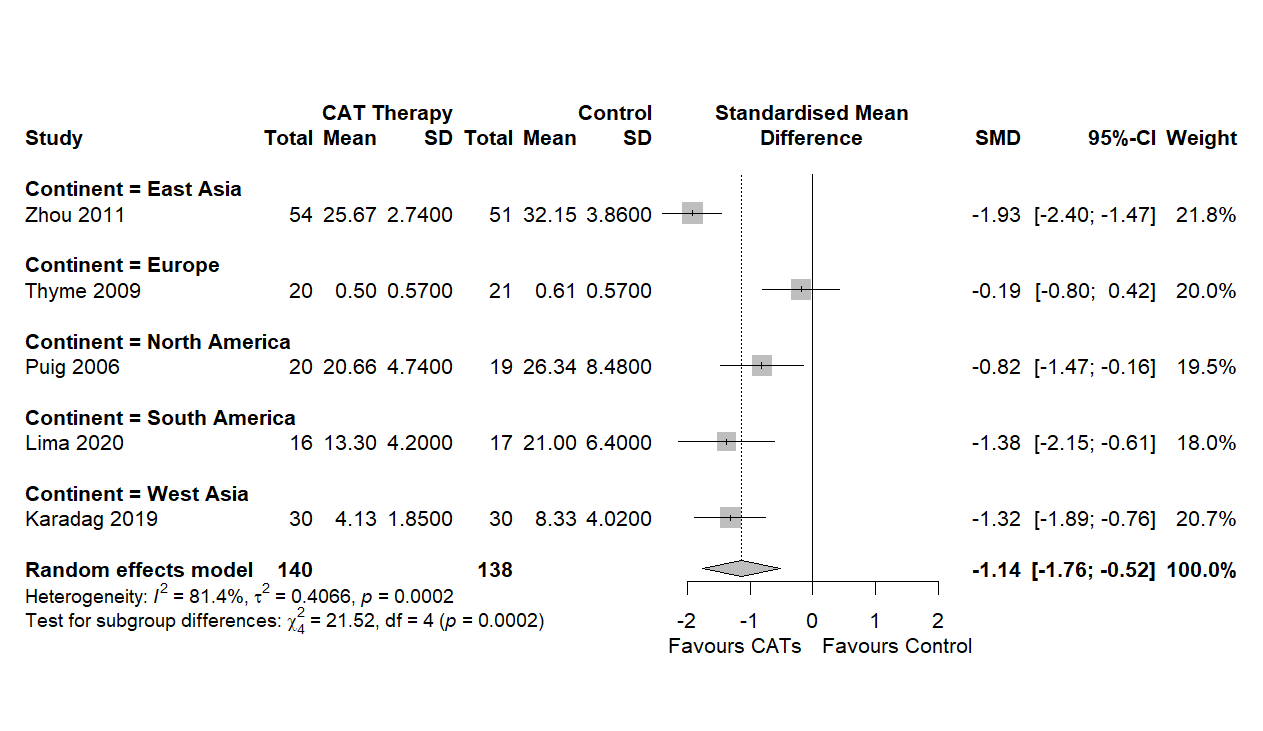
**Fig 42:** Forest plot for effect of CATs on depression between 4 to 6 weeks, by region.

**SECONDARY ANALYSIS – QUALITY OF LIFE**

*Intervention type*


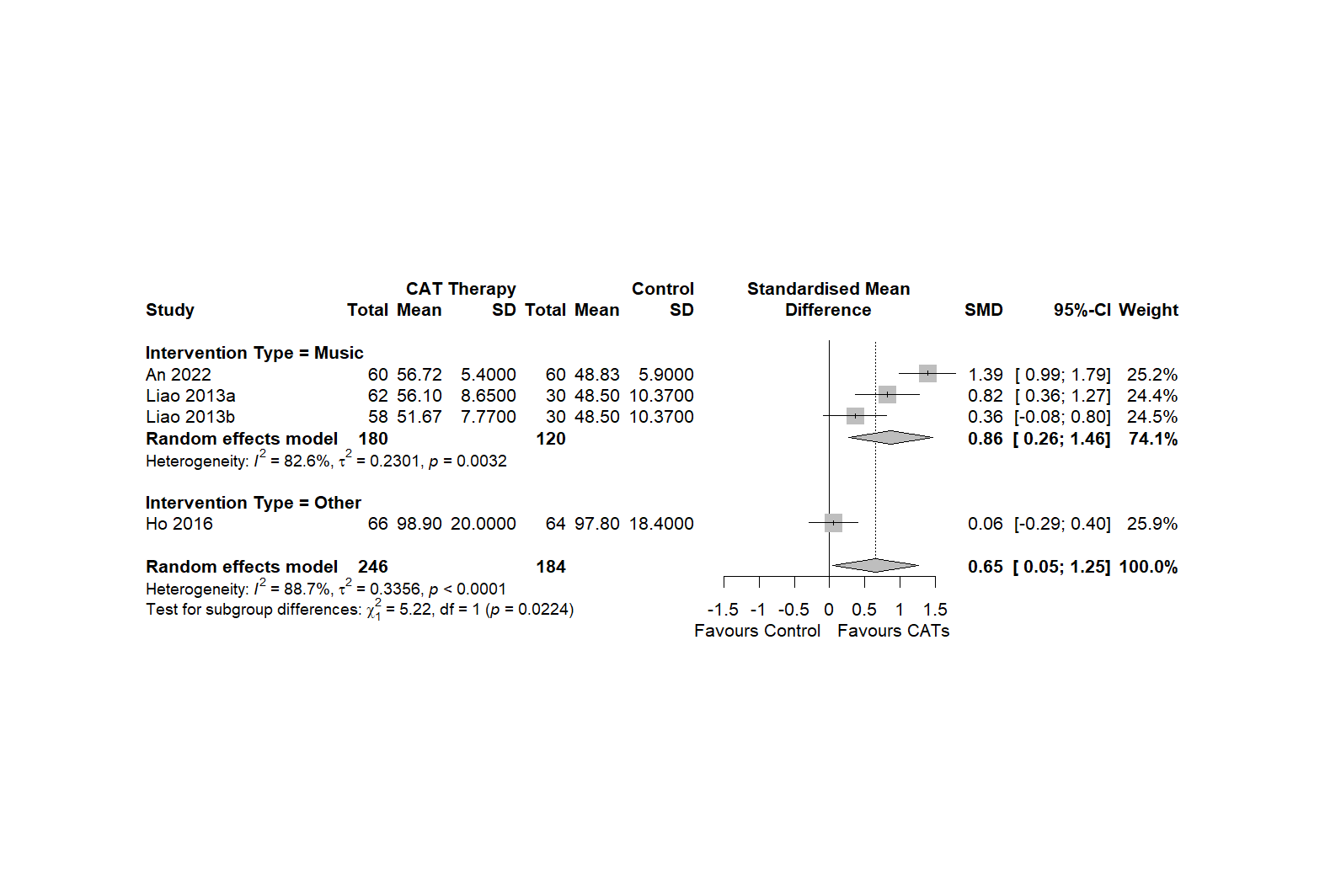


**Fig 43:** Forest plot for effect of CATs on quality of life between 1 to 3 weeks, by intervention type.

**
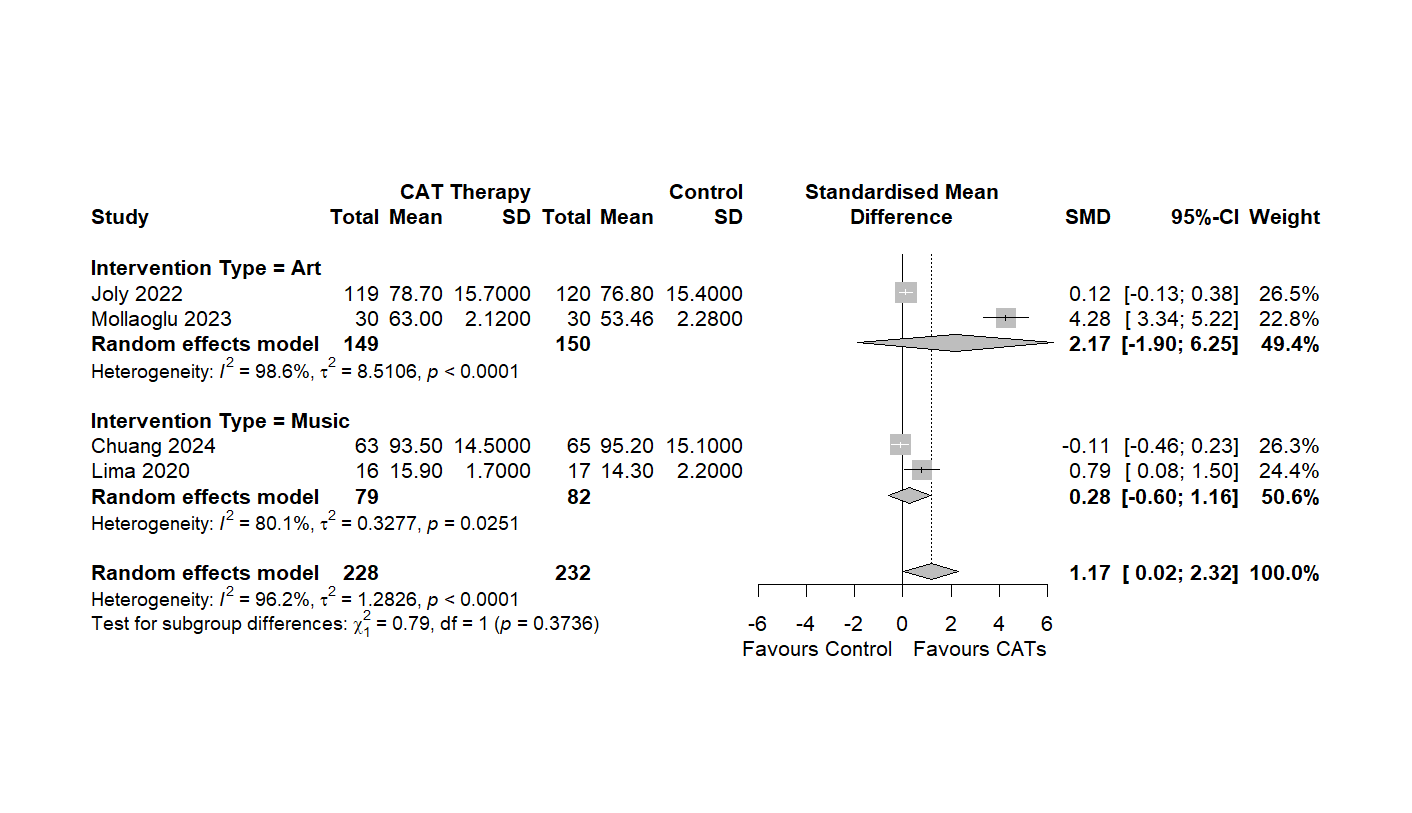
Fig 44:** Forest plot for effect of CATs on quality of life between 4 to 6 weeks, by intervention type.


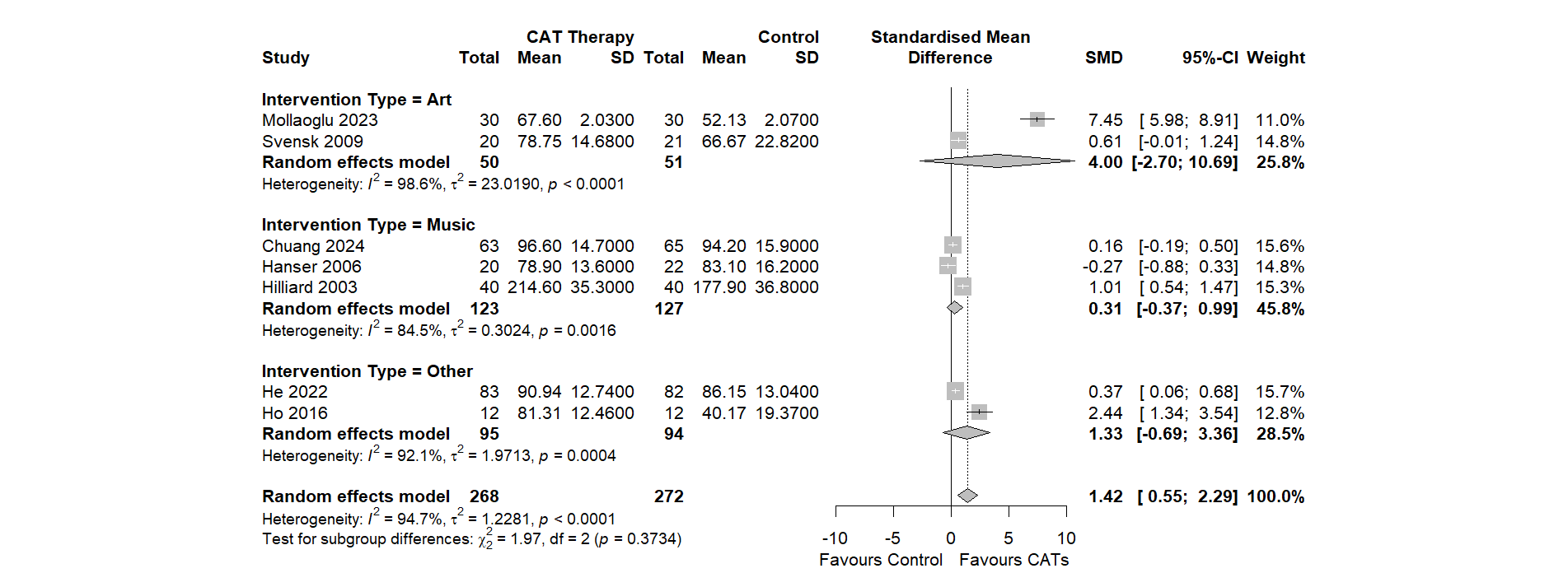
**Fig 45:** Forest plot for effect of CATs on quality of life between 2 to 3 months, by intervention type.

**
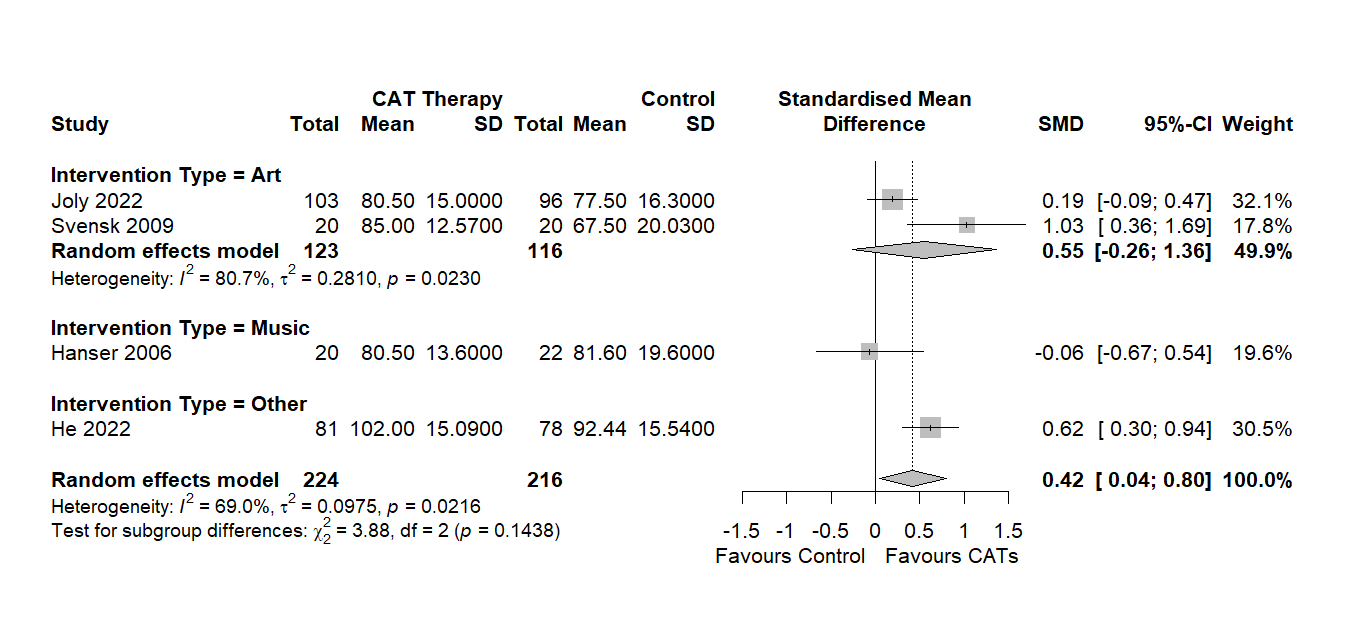
Fig 46:** Forest plot for effect of CATs on quality of life between 4 to 6 months, by intervention type.

*Treatment setting*


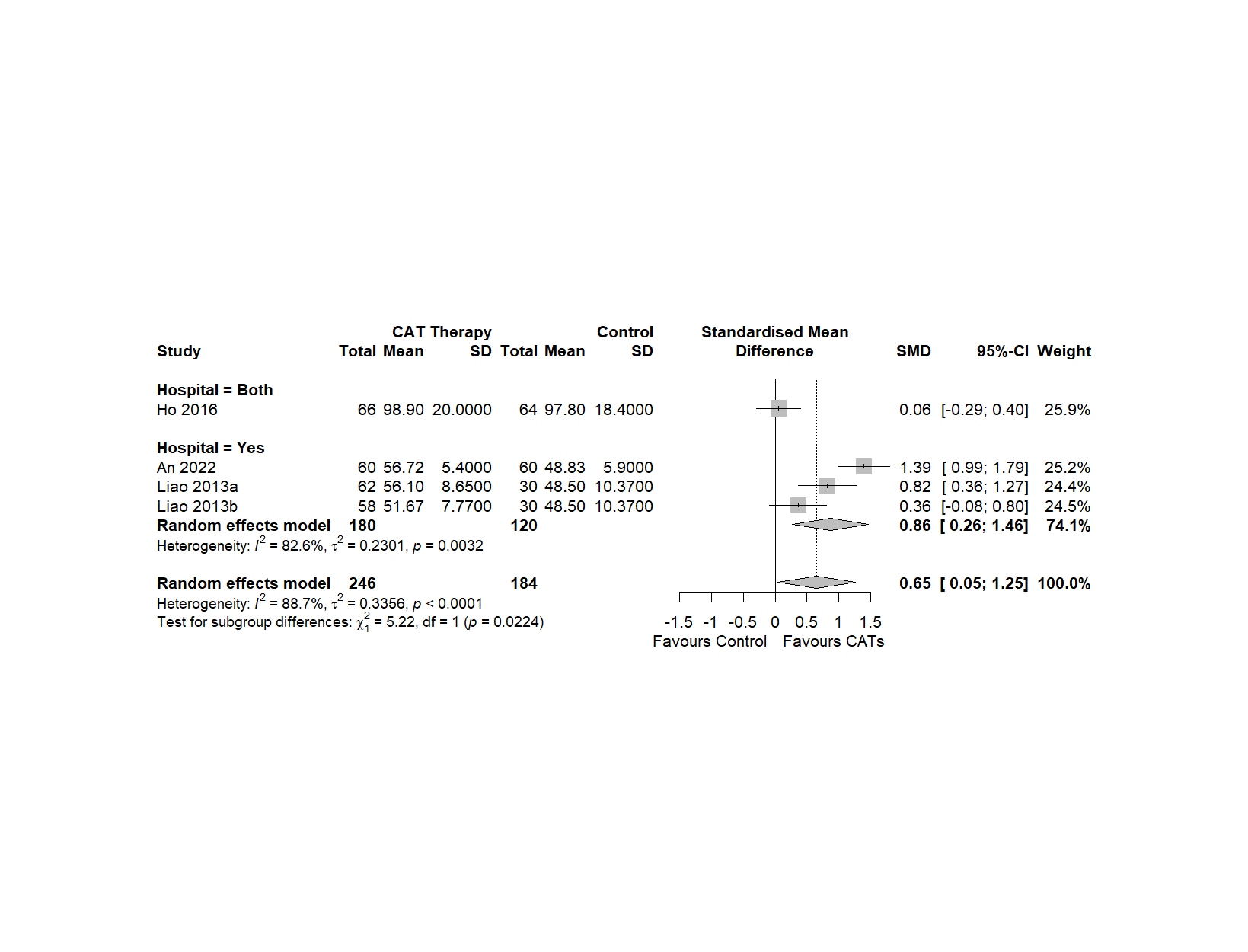
**Fig 47:** Forest plot for effect of CATs on quality of life between 1 to 3 weeks, by treatment setting.


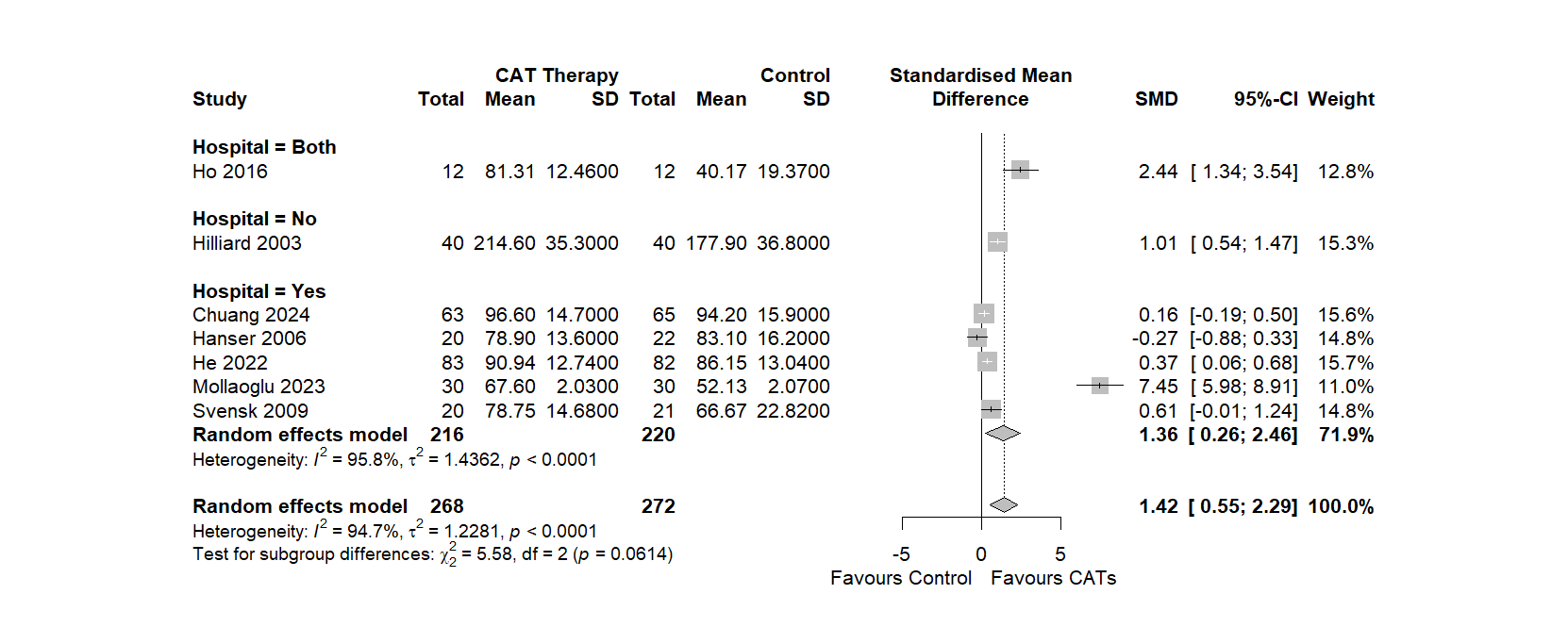
**Fig 48:** Forest plot for effect of CATs on quality of life between 2 to 3 months, by treatment setting.

*Region*

**
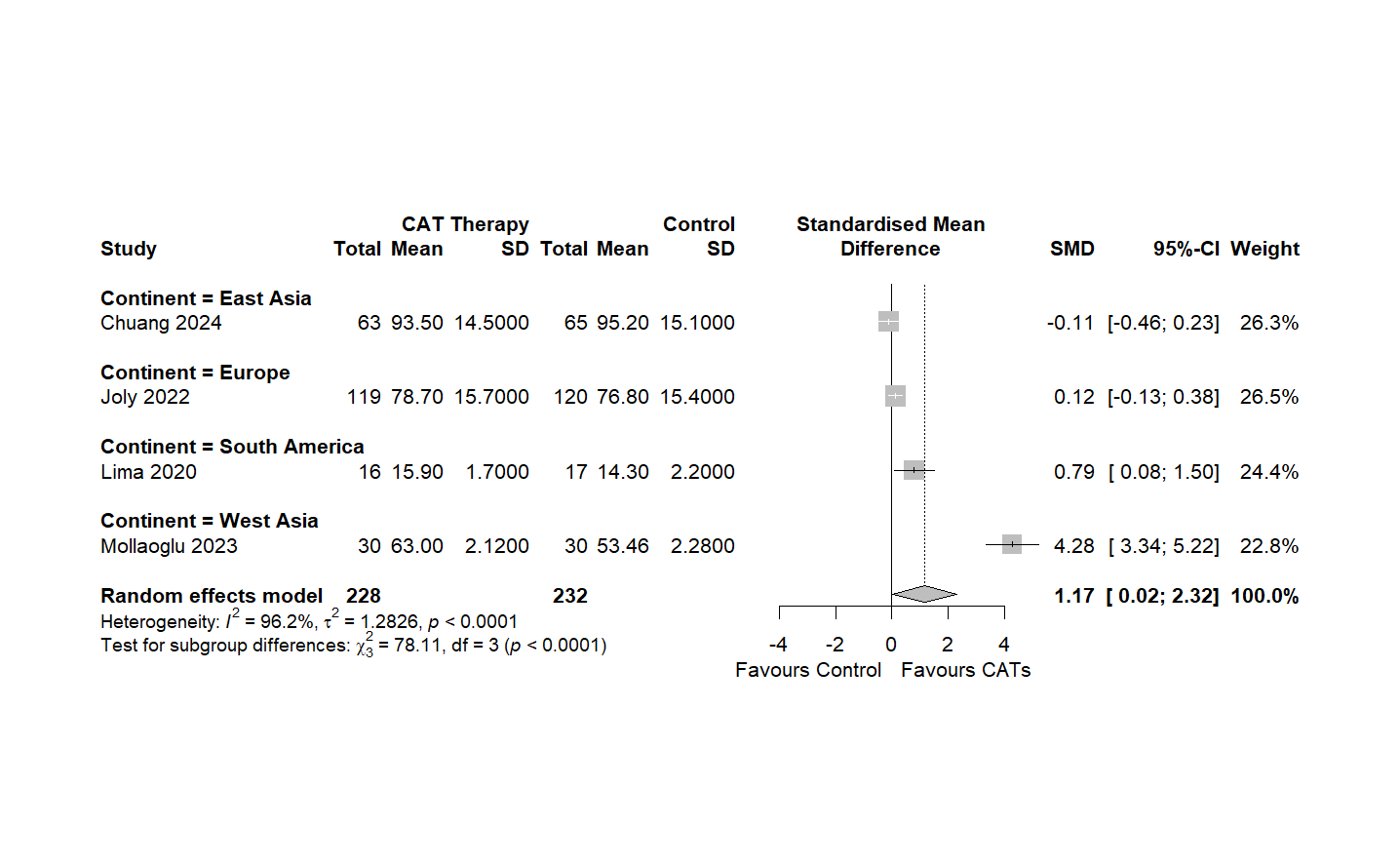
Fig 49:** Forest plot for effect of CATs on quality of life between 4 to 6 weeks, by region.


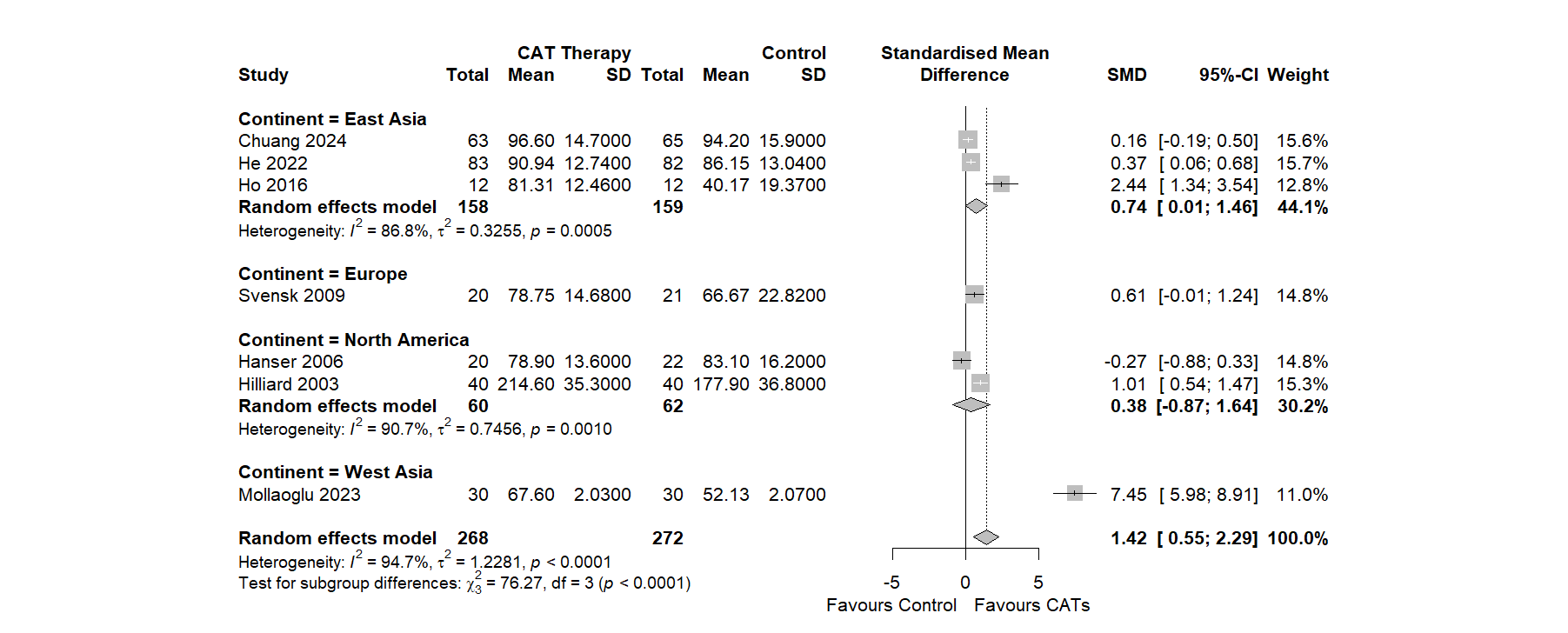
**Fig 50:** Forest plot for effect of CATs on quality of life between 2 to 3 months, by region.

**
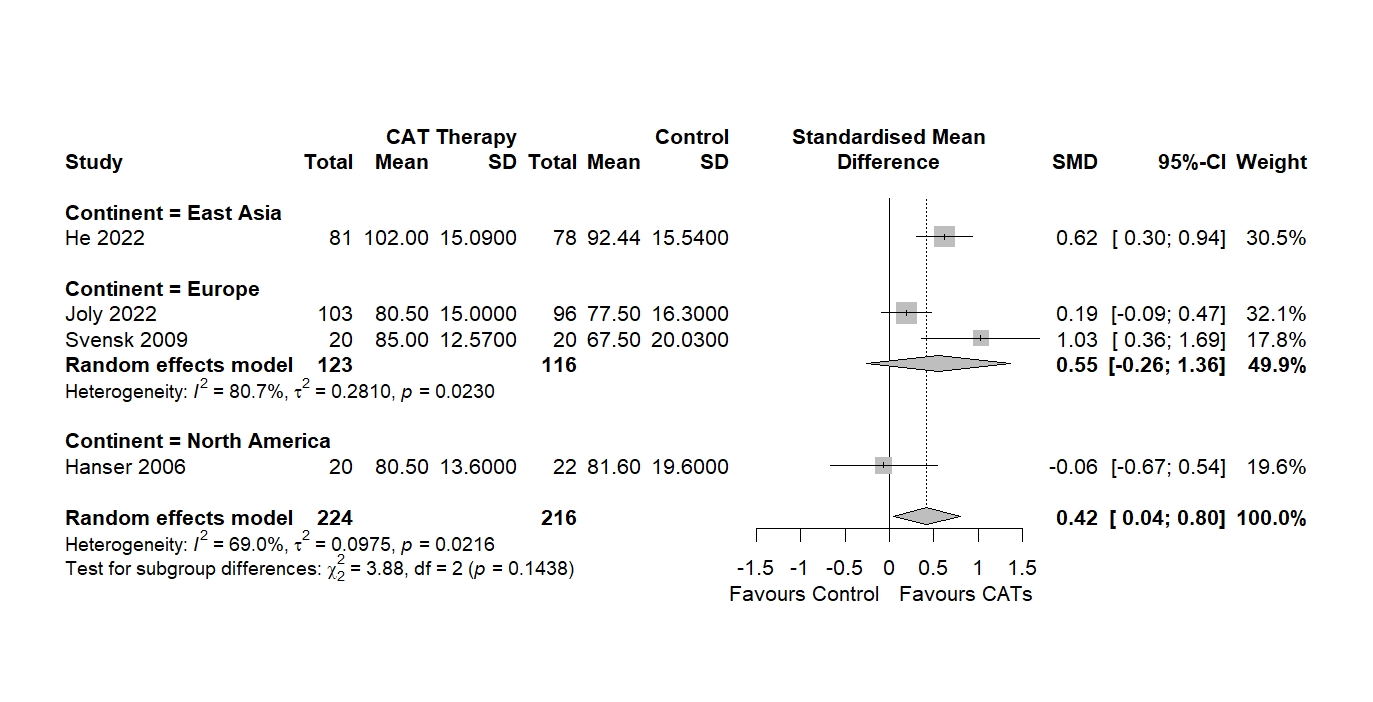
Fig 51:** Forest plot for effect of CATs on quality of life between 4 to 6 months, by region.
